# Supplementary material for: MicroRNAs miR-584-5p and miR-425-3p Are Up-Regulated in Plasma of Colorectal Cancer (CRC) Patients: Targeting with Inhibitor Peptide Nucleic Acids Is Associated with Induction of Apoptosis in Colon Cancer Cell Lines
Source: Cancers (Basel). 2022 Dec 25;15(1):128. doi: 10.3390/cancers15010128 (PMC9817681; doi:10.3390/cancers15010128)
Supplement: Supplementary file 1 [file cancers-15-00128-s001.zip › cancers-2052941 Supplementary.pdf]

cancers

MDPI

Article

MicroRNAs miR-584-5p and miR-425-3p Are Up-Regulated in Plasma of Colorectal Cancer (CRC) Patients: Targeting with Inhibitor Peptide Nucleic Acids Is Associated with Induction of Apoptosis in Colon Cancer Cell Lines

Jessica Gasparello [1], Chiara Papi [1], Matteo Zurlo [1], Laura Gambari [2], Alex Manicardi [3], Andrea Rozzi [3], Matteo Ferrarini [3], Roberto Corradini [3], Roberto Gambari [1,*] and Alessia Finotti [1,*]

Supplementary Materials

Supplementary Methods

SM1. *Synthesis and characterization of PNAs*

All reactants and solvents for PNA synthesis were analytical grade. Rink amide ChemMatrix®, Fmoc-glycine, acetic anhydride and m-cresol were obtained from Sigma (St Louis, MO, USA). Piperidine, N,N-Diisopropylethylamine (DIPEA) and trifluoroacetic acid (TFA) were from Alfa Aesar (Haverhill, Massachusetts, USA). N,N,N′,N′-Tetramethyl-O-(1H-benzotriazol-1-yl)uronium hexafluorophosphate (HBTU) was purchased from TCI Europe (Eschborn, Germany). N,N-Dimethylformamide (DMF) was from Scharlab (Barcelona, Spain). Fmoc-protected PNA monomers were purchased from LGC Link (Bellshill, Scotland).

Synthesis and characterization of R8-PNA-a15b (H-R8-TGTAAACCATGATGTGCT-Gly-NH2) have been reported elsewhere [35,36] and fully described in the Supplementary Materials section (SM1). Synthesis of R8-PNA-a584 and R8-PNA-a425 was performed as previously reported for other anti-miRNA PNAs [36]. Briefly, PNAs were synthesized with an automatic synthesizer Syro I following a Fmoc protocol on a glycine pre-loaded resin (Fmoc-Gly-Rink amide ChemMatrix® resin). Each cycle of synthesis was composed by three steps: deprotection of the N-terminal protective group Fmoc (piperidine 20% in DMF, 8 minutes, twice), coupling of the next commercial monomer (3 equivalents of the monomer and the activator HBTU, 6 equivalents of the non-nucleophilic base DIPEA, dry DMF, 40 minutes, twice) and capping of possible unreacted free amine (dry DMF, acetic anhydride, DIPEA, 89:5:6, 1 minute, twice). After the completion of the sequence, the ending Fmoc group was deprotected and the PNA cleaved from the resin using an appropriate cocktail (TFA, m-cresol 9:1, 1 hour, twice). The PNA was precipitated in diethyl ether and purified in reverse phase HPLC using the following conditions: column Phenomenex Jupiter RPC18, 250- 4.6mm,1.7 μM;T = 40°C Eluents: A (0.1% TFA in water), b (0.1% TFA in acetonitrile. Solvent program: flow rate: 4 ml/min; 100% A for 5 then gradient to 0-40% B in 23 min and 23-100B in 3 min.

The purified PNA samples were furher analysed using a Waters Acquity ultra-performance LC HO6UPS-823M, with Waters SQ detector equipped with Waters UPLC BEH C18, 50×2.1 mm, 1.7 m) at 35°C. A flow rate of 0.25 ml/min was used with the following solvent systems: (A): 0.2% FA in H_2O and (B): 0.2% FA in MeCN (FA = formic acid). Program: 0.9 min with solvent A, then a gradient from 0 to 50% B in 5.7 min, then isocratic at 50% B for 0.4 min and rapid change in 0.2 min to 100% B, a final wash in 100% B for 0.8 min. The chromatograms obtained and the relative MS spectra are reported in Supplementary Figures S1-3.

SM2. *Cell culture conditions*

The HT-29 and LoVo cell lines [35-36] were cultured in a humidified atmosphere of 5% CO2/air in RPMI 1640 medium (EuroClone, Pero, Milano, Italy) supplemented with 10% fetal bovine serum (FBS, Biowest, Nuaillé, France), 100 units/ml penicillin and 100 μg/ml streptomycin (Pen-Strep, Sigma-Aldrich). To verify the effect on proliferation, cell growth was monitored by determining the cell number/ml using a Z2 Coulter Counter (Coulter Electronics, Hialeah, FL, USA).

SM3. *Analysis of apoptosis: Annexin V Assay*

Apoptosis assays on treated or untreated cells were performed with Muse Cell Analyzer instrument (Millipore Corporation, Billerica, MA, USA), and its relative assays according to the instructions supplied by the manufacturer. Muse Annexin V & Dead Cell Kit utilizes Annexin V to detect Phosphatidyl Serine (PS) on the external membrane of apoptotic cells. A fluorescent DNA intercalator (7-ADD: 7-aminoactinomycin D) is used as indicator of cell membrane integrity, indeed, 7-ADD is excluded from live, healthy cells, as well as, early apoptotic cells, while is able to bind DNA in late apoptosis and dead cells. Four populations of cells can be distinguished in this assay: cells negative to both

reagents (live cells), cells positive to Annexin V, but negative to 7-AAD (early apoptotic cells), cells negative to Annexin V and positive to 7-ADD (cellular debris) and cells positive to both reagents (late apoptotic cells). Cells were washed with sterile PBS 1X, detached by trypsinization, suspended and diluted (1:2) with the Muse Annexin V & Dead Cell reagent. Samples were incubated at room temperature, protected from the light for 15 minutes and at the end of incubation, analyzed using Muse Cell Analyzer and Annexin V and Dead Cell Software Module (Millipore) for data elaboration [39].

SM4. *Analysis of apoptosis: Caspase 3/7 Assay*

The apoptotic status based on Caspase-3/7 activation was studied using Muse Caspase-3/7 Kit. The assay is based on the use of a DNA-binding dye, linked to a DEVD peptide substrate. When bound to DEVD the dye is unable to bind DNA, while the cleavage by active Caspase-3/7 in the cell results in release of the dye that moves to the nucleus and binds the DNA giving a fluorescent signal. 7-ADD is used as indicator of cell membrane integrity. Briefly, cells were detached, washed with DPBS 1X and 50 µL of cell suspension cells were incubated with 5 µL of Caspase-3/7 working solution (obtained from 1:8 dilution of Muse Caspase-3/7 Reagent with 1X PBS). After an incubation of 30 minutes at 37°C, 150 µl of 7-AAD working solution (obtained from 1:75 dilution of 7-ADD in 1X Assay Buffer BA) were added, and the mixture was incubated for 5 minutes at room temperature, protected from the light. Samples were analyzed using Muse Cell Analyzer instrument and Caspase-3/7 software.

Supplementary Figures

Figure S1 UPLC-ESI/MS analysis of R8-PNA-a425. a) UPLC chromatogram, middle: ESI-MS spectrum of peaks at b) 2.95 and c) 2.59 min; d) mathematical deconvolution of the multicharged signals. Conditions are as indicated above.

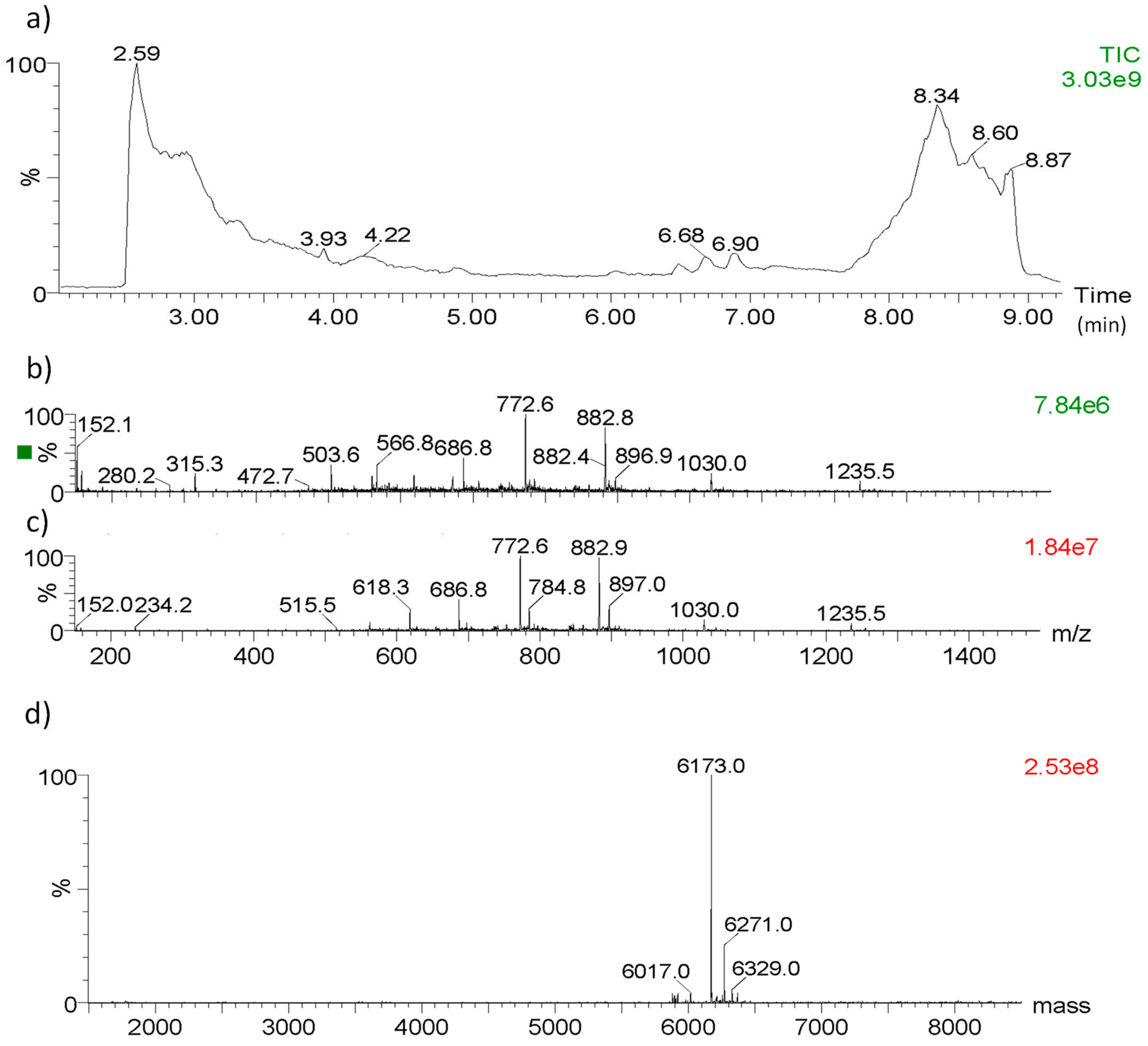

Figure S2 UPLC-ESI/MS analysis of R8-PNA-a584. a) UPLC chromatogram, b) ESI-MS spectrum (positive mode) of peak at 2.57 min; c) mathematical deconvolution of the multicharged signals. Conditions are as indicated above.

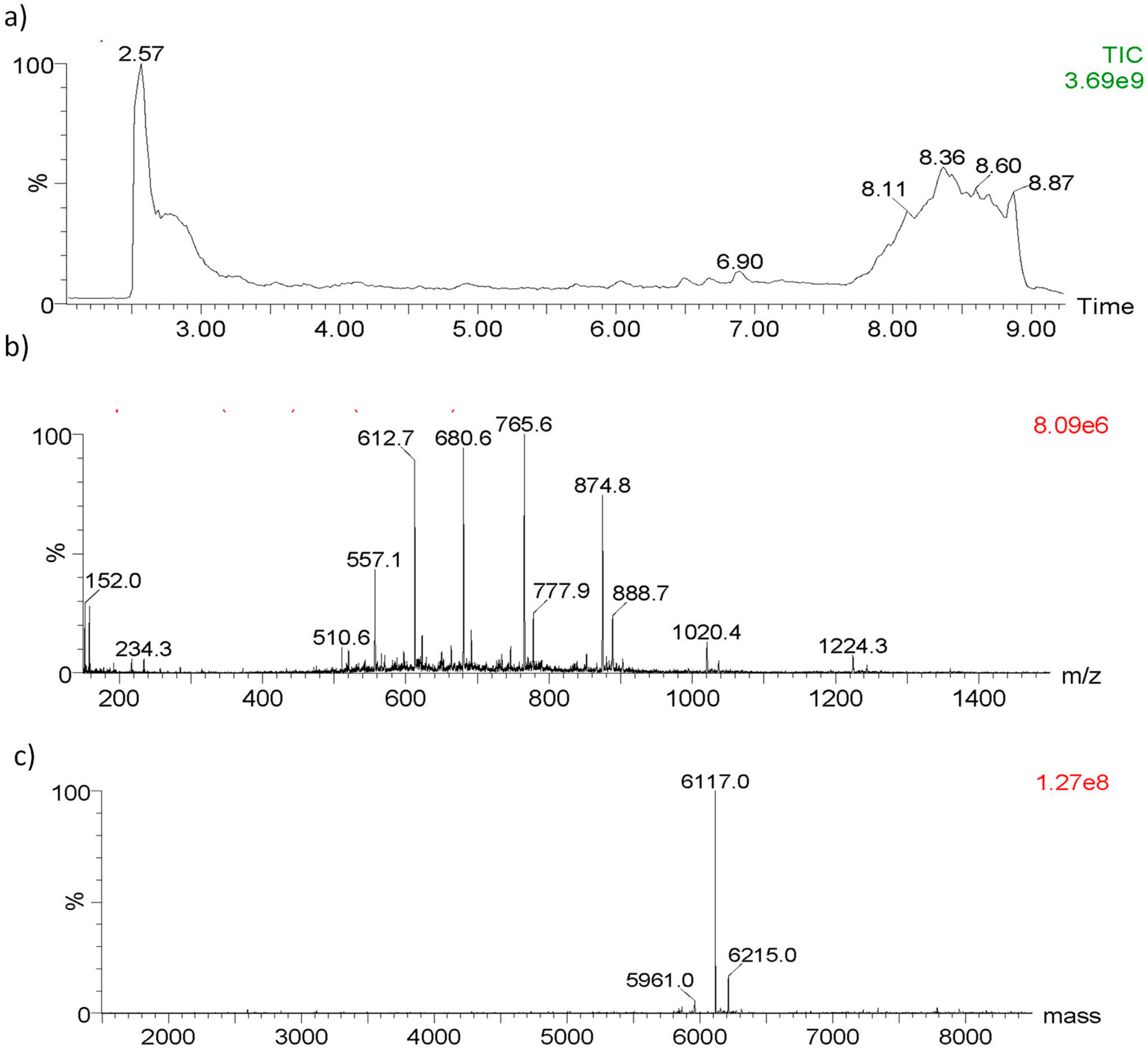

Figure S3 UPLC-ESI/MS analysis of R8-PNA-control (unrelated sequence). Above UPLC chromatogram, middle: ESI-MS spectrum of peak at 2.50 min; below: mathematical deconvolution of the multicharged signals. Conditions are as indicated above.

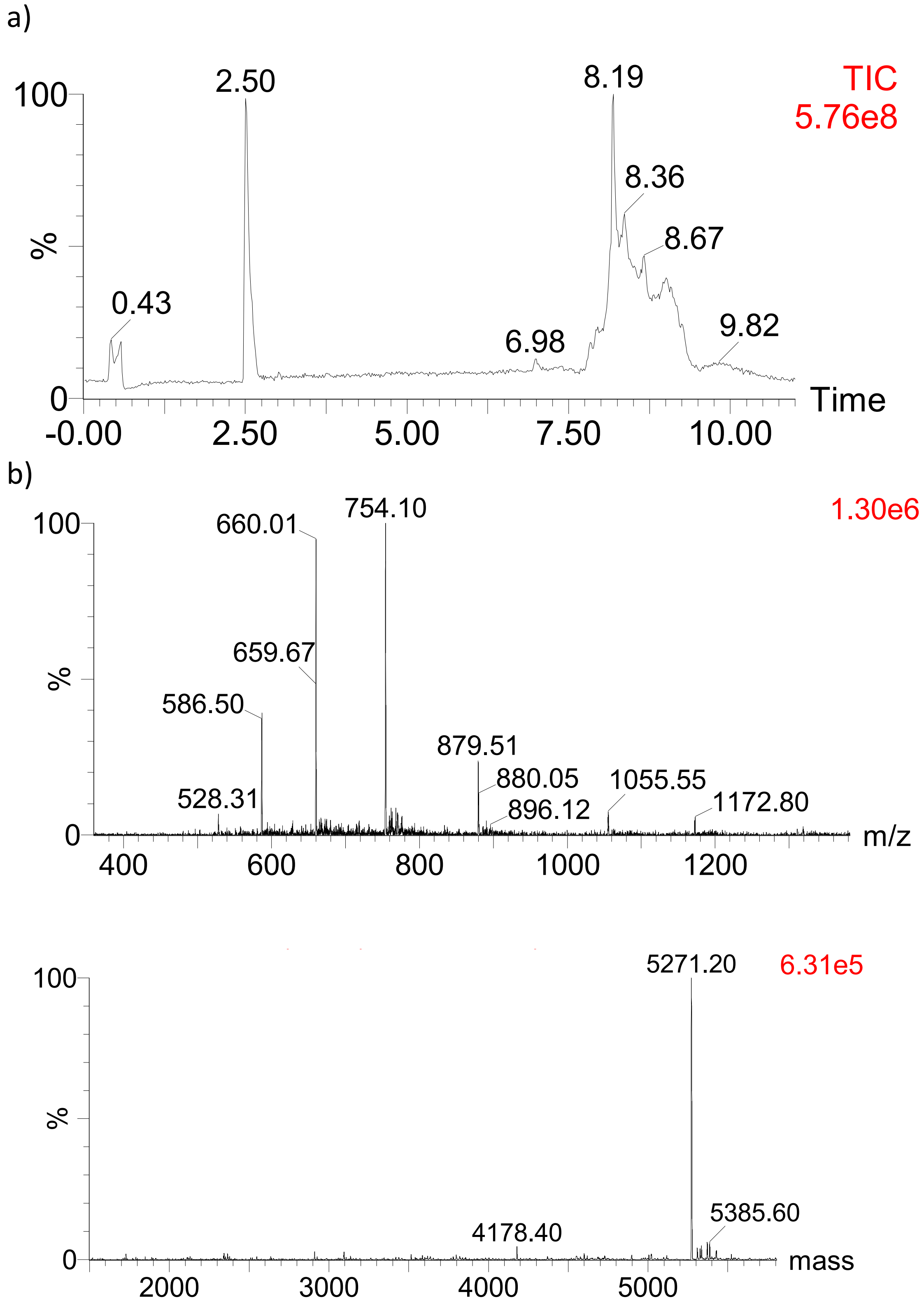

Figure S4: Quantification of miR-584-5p and miR-425-3p CRC cellular models. (**A**) Quantification by RT-ddPCR of hsa-miR-584-5p in three different CRC cellular models: LoVo, Ls174T and HT-29. (**B**) has-miR-425-3p was quantified by RT-ddPCR in CRC cellular models. 2D RT-ddPCR plots are reported. (**C**) The three cellular models are compared for their miR-584-5p and miR-425-3p content.

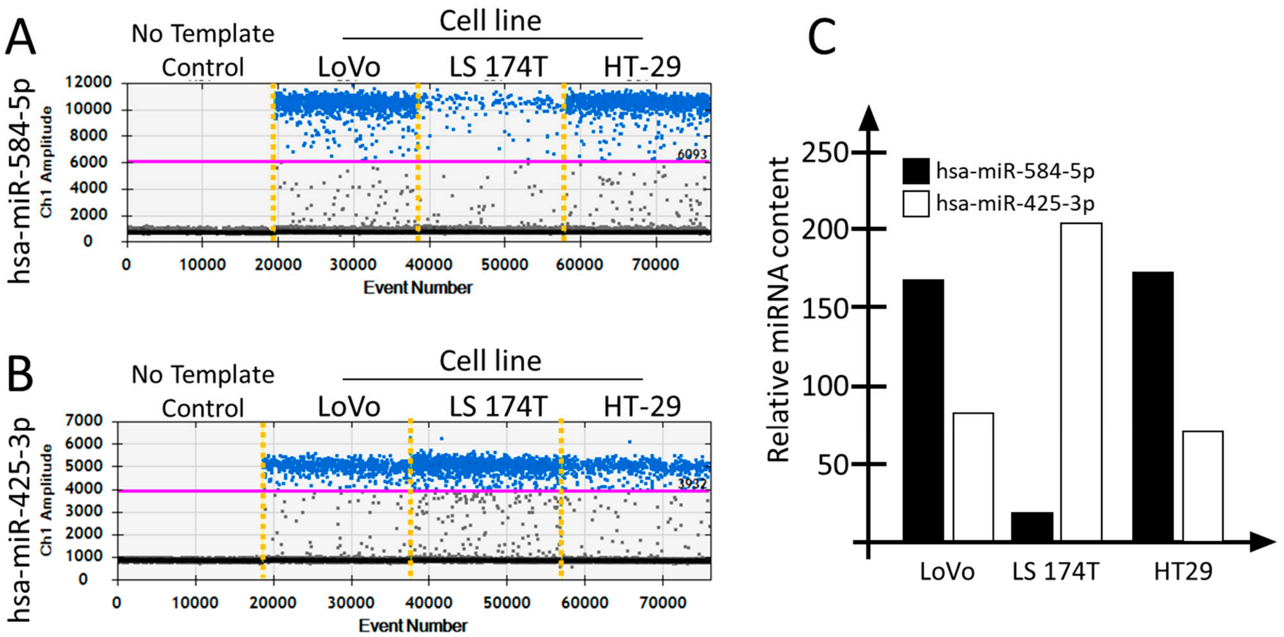

Figure S5: Effects of unrelated-seq PNA on apoptosis. (**A**) Effects of un-related seq PNA on intracellular levels of the three studied miRNAs (miR-15b-5p, miR-425-3p and miR-584-5p) were detected by RT-qPCR. (**B**) Apoptosis profile was investigated in HT-29 cells treated with increasing concentrations (2-12 μM) of un-related seq PNA.

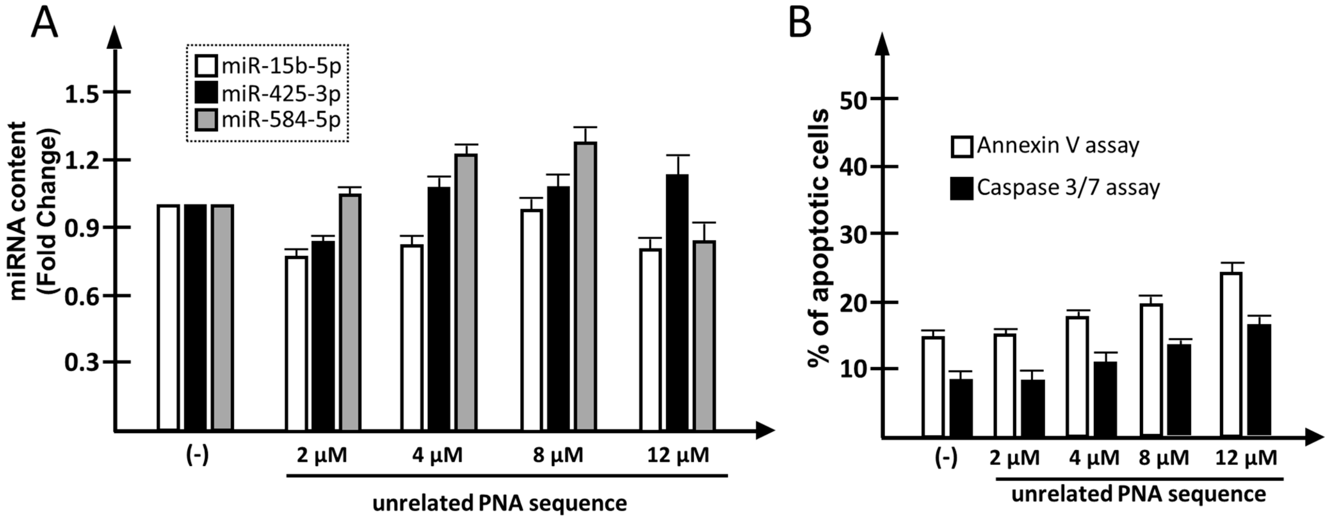

Figure S6: miRNAs levels in multiple miRNAs-targeting treatments. microRNAs levels were detected after 72 hours of treatment with singular or multiple anti-miRNA PNAs in (**A**) HT-29 cell line and (**B**) LoVo cells. Intracellular levels of miR-15b-5p (white boxes), miR-425-3p (light grey boxes), miR-584-5p (dark grey boxes) or the unrelated miR-210-3p (black boxes), were detected by RT-qPCR.

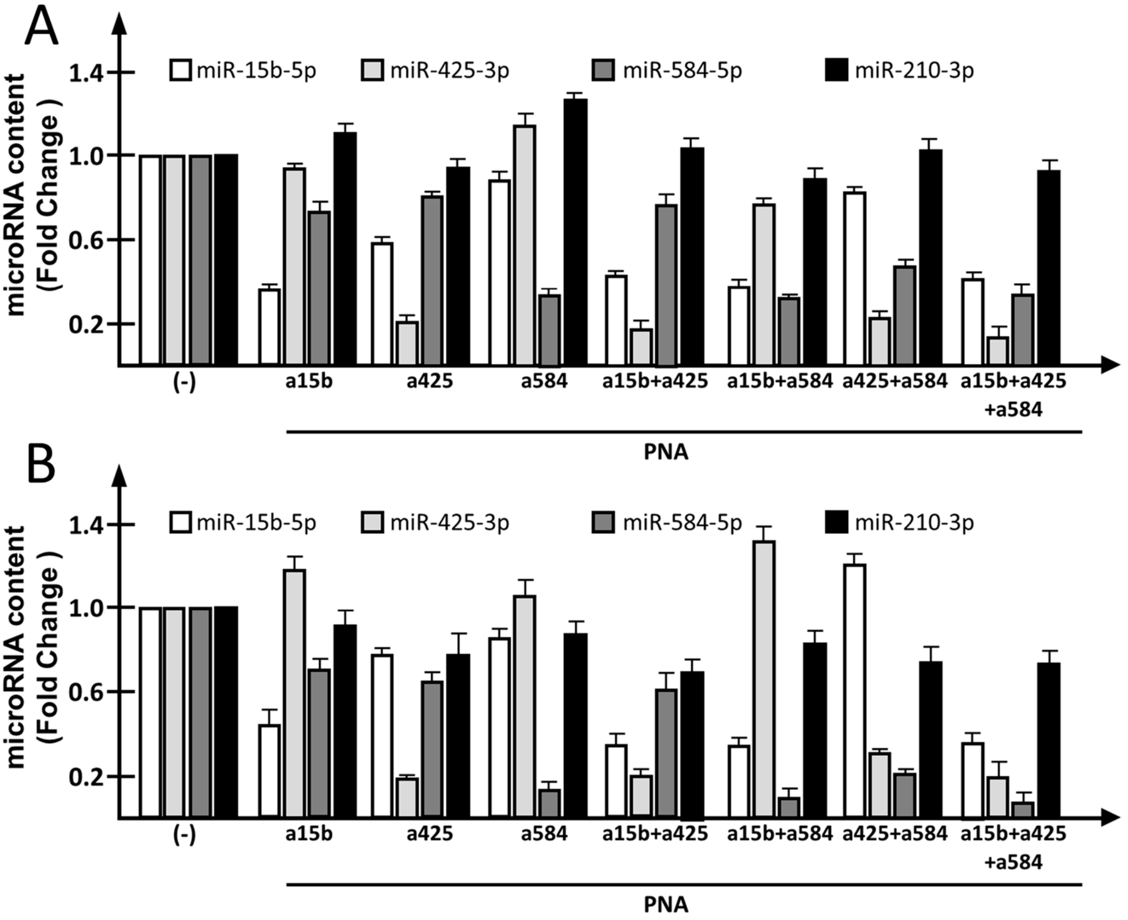

Figure S7: Exemplificative apoptosis plots of simultaneous targeting of miR-584-5p, miR-425-3p, and miR-15b-5p. (**A**) Plots of Annexin V assay for HT-29 cell line (**B**) Plots of Caspase 3/7 assay for HT-29 cell line (**C**) Plots of Annexin V assay for LoVo cell line (**D**) Plots of Caspase 3/7 assay for LoVo cell line

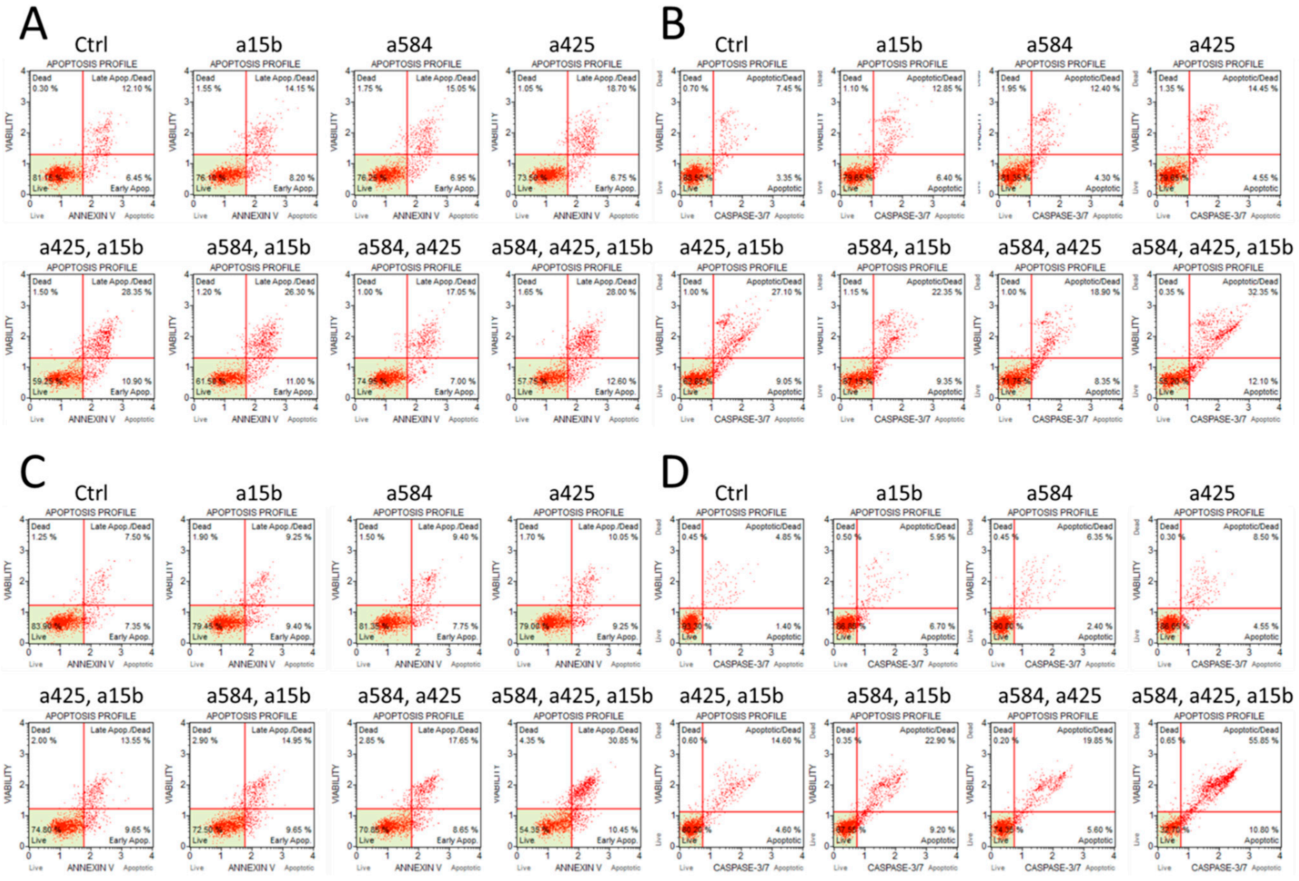

Figure S8: Evaluation of pro-apoptotic effects of SFN in the CRC cellular models: HT-29 and LoVo. (**A,B**) Representative plots of Annexin V assay in HT-29 (**A**) and LoVo (**B**) cell lines. Comparison of apoptotic profile in HT-29 and LoVo cells after 72 hours of contact with sulforaphane.

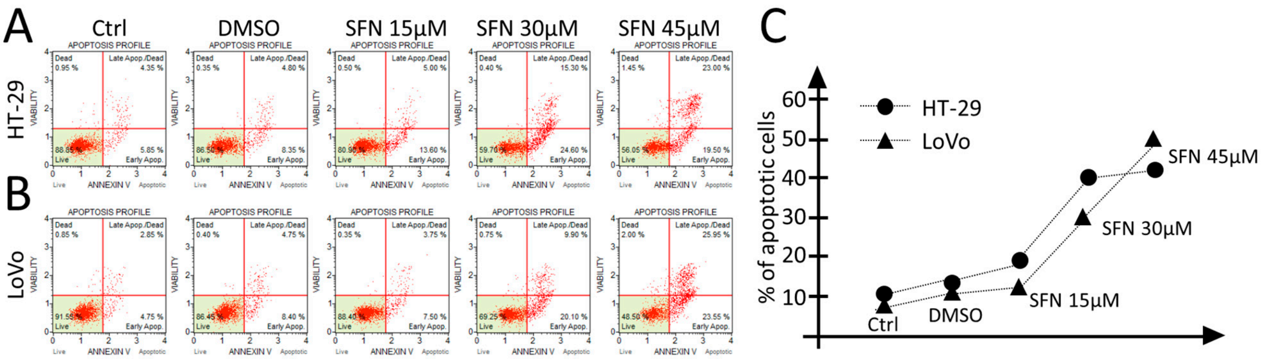

Figure S9: Exemplificative apoptosis plots of simultaneous treatments with PNA targeting miR-584-5p or miR-425-3p and SFN. Representative plots of Annexin V assay for the combinations SFN plus R8-PNA-a584 (**A**) or R8-PNA-a425 (**B**) for HT-29 cells. Representative plots of Annexin V assay for the combinations SFN plus R8-PNA-a584 (**C**) or R8-PNA-a425 (**D**) for LoVo treated cells.

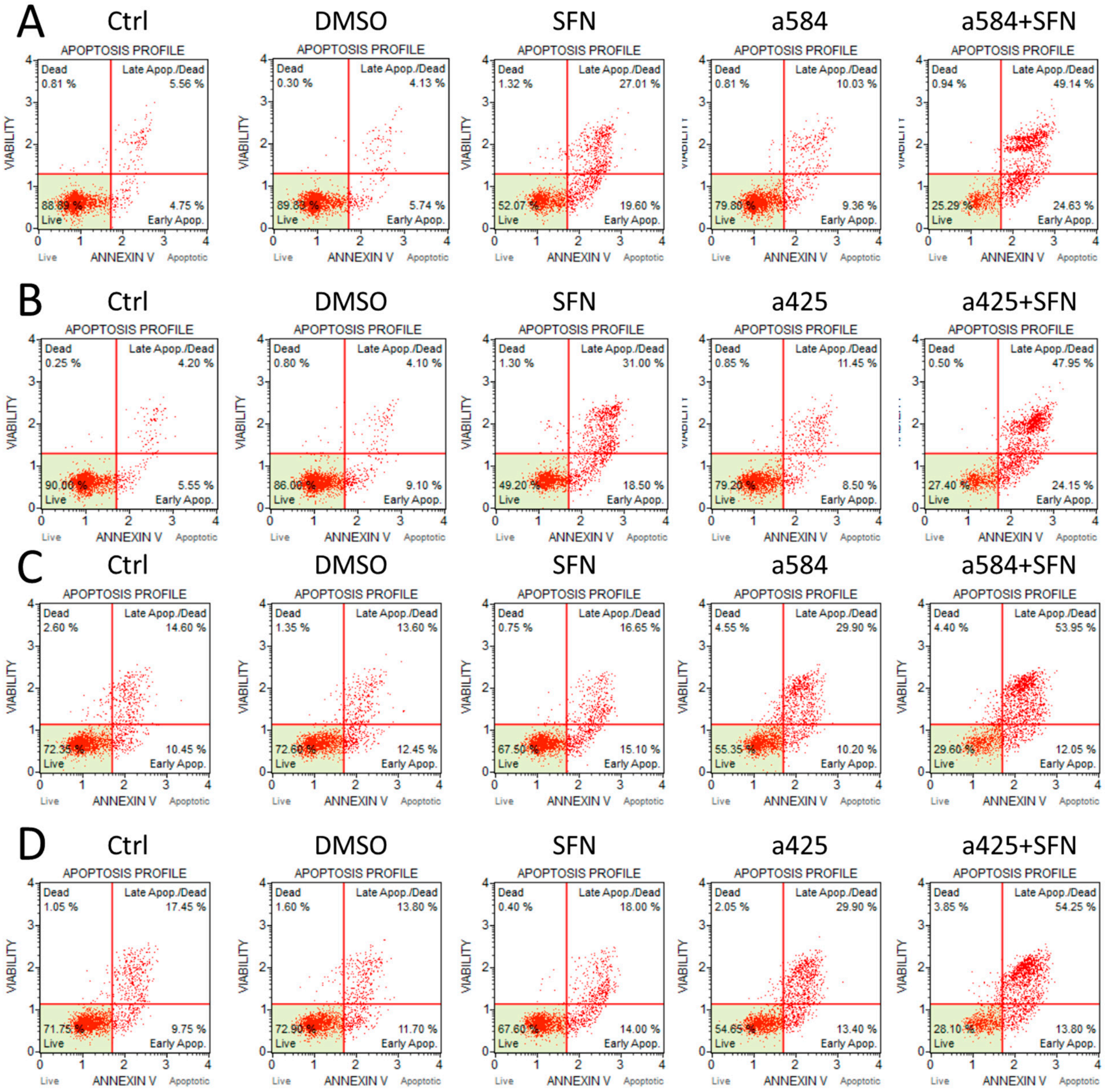

Figure S10. Combination of unrelated-sequence PNA and sulforaphane. (**A**) Apoptosis induction in HT-29 cells treated for 72 hours with the unrelated sequence PNA additioned with sulforaphane or DMSO (used as SFN diluent) (**B**) Representative Annexin V assay plots. The data included in panel A were obtained by subtracting the SFN values to that of control DMSO-treated cells.

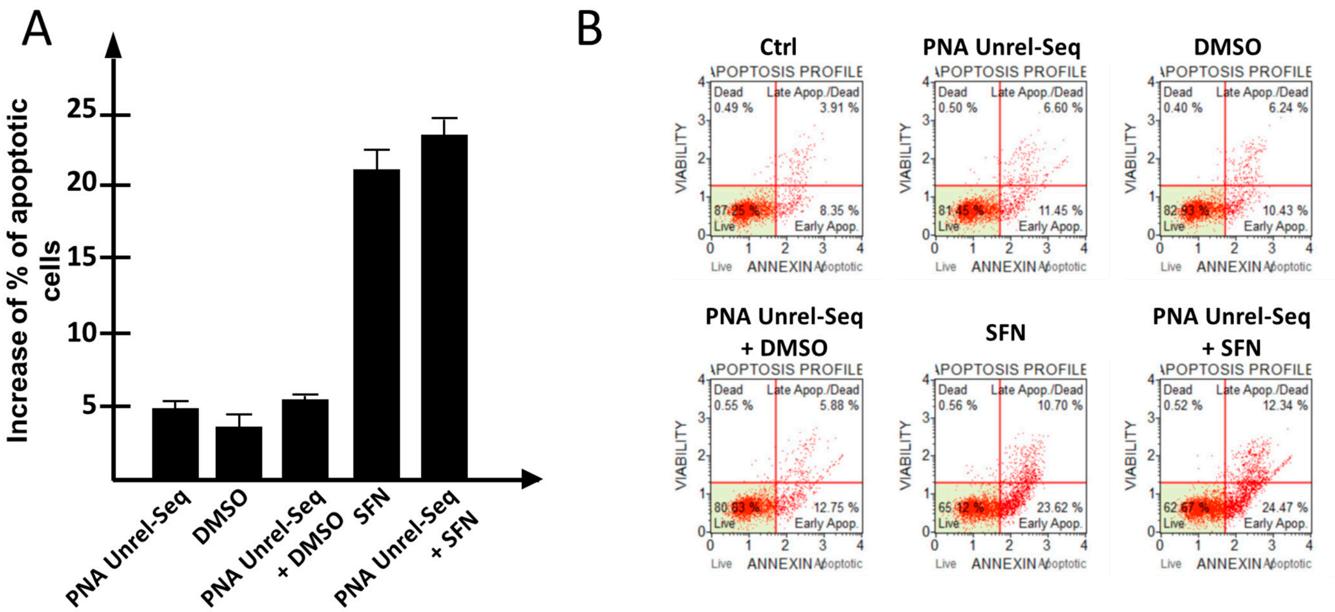

Supplementary Tables

Table S1. Predicted and validated mRNAs targeted by miR-425-3p. A total of 928 transcripts were found to potentially be targeted by miR-425-3p, with a total of 995 sites, between them 45 were experimentally validated (indicated in table by a check mark). Data were obtained by a combined bioinformatical approach using TargetScan Human v8.0 to identify mRNAs predicted to be bounded by miR-425-3p and miRTarBase release 9 to identify validated miRNA-mRNA interactions. Both databases were accessed on December 6th.

Target gene	Gene name	Validated	Target gene	Gene name	Validated
**ORAOV1**	*oral cancer overexpressed 1*		**KIAA1239**	*KIAA1239*	
**B3GALT5**	*UDP-Gal:betaGlcNAc beta 1,3-galactosyltransferase, polypeptide 5*		**PACS1**	*phosphofurin acidic cluster sorting protein 1*	
**AOC3**	*amine oxidase, copper containing 3*		**TPPP**	*tubulin polymerization promoting protein*	
**PER2**	*period circadian clock 2*	✓	**SPG7**	*spastic paraplegia 7 (pure and complicated autosomal recessive)*	
**OTX1**	*orthodenticle homeobox 1*		**BRD3**	*bromodomain containing 3*	
**UBALD2**	*UBA-like domain containing 2*		**DNAJC28**	*DnaJ (Hsp40) homolog, subfamily C, member 28*	
**FOXL2**	*forkhead box L2*		**C5orf22**	*chromosome 5 open reading frame 22*	
**PRX**	*periaxin*		**INPP5D**	*inositol polyphosphate-5-phosphatase, 145kDa*	
**OBP2B**	*odorant binding protein 2B*		**FA2H**	*fatty acid 2-hydroxylase*	
**ANGEL2**	*angel homolog 2 (Drosophila)*		**BBS5**	*Bardet-Biedl syndrome 5*	
**KIFC2**	*kinesin family member C2*		**CBLN1**	*cerebellin 1 precursor*	
**TSEN2**	*TSEN2 tRNA splicing endonuclease subunit*		**WNT2B**	*wingless-type MMTV integration site family, member 2B*	
**AGPAT9**	*1-acylglycerol-3-phosphate O-acyltransferase 9*		**ZNF841**	*zinc finger protein 841*	
**SYPL2**	*synaptophysin-like 2*		**ADAMTS20**	*ADAM metallopeptidase with thrombospondin type 1 motif, 20*	
**LCNL1**	*lipocalin-like 1*		**DUSP7**	*dual specificity phosphatase 7*	
**CST9**	*cystatin 9 (testatin)*		**ATF7IP2**	*activating transcription factor 7 interacting protein 2*	
**CD300LG**	*CD300 molecule-like family member g*		**CEBPA**	*CCAAT/enhancer binding protein (C/EBP), alpha*	

**ZCCHC24**	*zinc finger, CCHC domain containing 24*		**HEG1**	*heart development protein with EGF-like domains 1*	
**GATA2**	*GATA binding protein 2*		**UCP2**	*uncoupling protein 2 (mitochondrial, proton carrier)*	
**ATG9B**	*autophagy related 9B*		**AP1G1**	*adaptor-related protein complex 1, gamma 1 subunit*	
**RTBDN**	*retbindin*		**IFNAR2**	*interferon (alpha, beta and omega) receptor 2*	
**IGF2**	*insulin-like growth factor 2 (somatomedin A)*		**TMEM254**	*transmembrane protein 254*	
**RAC1**	*ras-related C3 botulinum toxin substrate 1 (rho family, small GTP binding protein Rac1)*	✓	**CACNA1G**	*calcium channel, voltage-dependent, T type, alpha 1G subunit*	
**CXCL9**	*chemokine (C-X-C motif) ligand 9*		**ZBTB37**	*zinc finger and BTB domain containing 37*	
**AL627309.1**	*Uncharacterized protein*		**RPL37**	*ribosomal protein L37*	
**GPR143**	*G protein-coupled receptor 143*		**KLHL3**	*kelch-like family member 3*	
**EPB41L5**	*erythrocyte membrane protein band 4.1 like 5*		**TTPAL**	*tocopherol (alpha) transfer protein-like*	
**C11orf49**	*chromosome 11 open reading frame 49*		**HDLBP**	*high density lipoprotein binding protein*	✓
**AL078585.1**	*Uncharacterized protein; cDNA FLJ58069*		**DCAF16**	*DDB1 and CUL4 associated factor 16*	
**ZNF705D**	*zinc finger protein 705D*		**PLCB1**	*phospholipase C, beta 1 (phosphoinositide-specific)*	✓
**ZNF705B**	*zinc finger protein 705B*		**SPAG9**	*sperm associated antigen 9*	
**CIITA**	*class II, major histocompatibility complex, transactivator*		**PITPNM3**	*PITPNM family member 3*	
**KLHL5**	*kelch-like family member 5*		**LRRC14**	*leucine rich repeat containing 14*	
**C2orf50**	*chromosome 2 open reading frame 50*		**SLC12A3**	*solute carrier family 12 (sodium/chloride transporter), member 3*	
**CRISPLD2**	*cysteine-rich secretory protein LCCL domain containing 2*		**TMEM200B**	*transmembrane protein 200B*	
**NOL12**	*nucleolar protein 12*		**PDE3A**	*phosphodiesterase 3A, cGMP-inhibited*	
**DUSP3**	*dual specificity phosphatase 3*		**PPARA**	*peroxisome proliferator-activated receptor alpha*	
**ZNF705A**	*zinc finger protein 705A*		**ST8SIA1**	*ST8 alpha-N-acetyl-neuraminide alpha-2,8-sialyltransferase 1*	
**CPA5**	*carboxypeptidase A5*		**C17orf103**	*chromosome 17 open reading frame 103*	

**USP40**	*ubiquitin specific peptidase 40*		**KSR2**	*kinase suppressor of ras 2*	
**CDHR5**	*cadherin-related family member 5*		**ZADH2**	*zinc binding alcohol dehydrogenase domain containing 2*	
**BTBD19**	*BTB (POZ) domain containing 19*		**SOGA3**	*SOGA family member 3*	
**C17orf80**	*chromosome 17 open reading frame 80*		**FLYWCH1**	*FLYWCH-type zinc finger 1*	
**LMOD3**	*leiomodin 3 (fetal)*		**PAICS**	*phosphoribosylaminoimidazole carboxylase, phosphoribosylaminoimidazole succinocarboxamide synthetase*	
**RMI2**	*RecQ mediated genome instability 2*		**GABRE**	*gamma-aminobutyric acid (GABA) A receptor, epsilon*	
**AKT1S1**	*AKT1 substrate 1 (proline-rich)*		**ONECUT3**	*one cut homeobox 3*	
**IKZF1**	*IKAROS family zinc finger 1 (Ikaros)*		**ZFAND3**	*zinc finger, AN1-type domain 3*	
**ART3**	*ADP-ribosyltransferase 3*		**KIF26A**	*kinesin family member 26A*	
**REM2**	*RAS (RAD and GEM)-like GTP binding 2*		**WNT7A**	*wingless-type MMTV integration site family, member 7A*	
**CD300LB**	*CD300 molecule-like family member b*		**RRM2B**	*ribonucleotide reductase M2 B (TP53 inducible)*	
**FLJ00104**	*HCG1980662; Uncharacterized protein*		**GPR75**	*G protein-coupled receptor 75*	
**RTN3**	*reticulon 3*		**COX19**	*cytochrome c oxidase assembly homolog 19 (S. cerevisiae)*	
**NRARP**	*NOTCH-regulated ankyrin repeat protein*		**NT5C1A**	*5'-nucleotidase, cytosolic IA*	
**CCDC106**	*coiled-coil domain containing 106*		**MUC13**	*mucin 13, cell surface associated*	
**TMEM219**	*transmembrane protein 219*		**IDS**	*Iduronate 2-sulfatase (Hunter syndrome), isoform CRA_e; Iduronate 2-sulfatase 14 kDa chain; cDNA FLJ42669 fis, clone BRAMY2022168, highly similar to IDURONATE 2-SULFATASE*	
**MAPT**	*microtubule-associated protein tau*		**KLF12**	*Kruppel-like factor 12*	
**MED1**	*mediator complex subunit 1*		**PPP2R2C**	*protein phosphatase 2, regulatory subunit B, gamma*	
**SYCE1L**	*synaptonemal complex central element protein 1-like*	✓	**RPL22L1**	*ribosomal protein L22-like 1*	
**WFDC3**	*WAP four-disulfide core domain 3*		**MYH10**	*myosin, heavy chain 10, non-muscle*	
**SMR3B**	*submaxillary gland androgen regulated protein 3B*		**TSPAN11**	*tetraspanin 11*	

**TCF25**	*transcription factor 25 (basic helix-loop-helix)*		**UNC5C**	*unc-5 homolog C (C. elegans)*	
**GPHN**	*gephyrin*		**PLEKHA1**	*pleckstrin homology domain containing, family A (phosphoinositide binding specific) member 1*	
**LEPROT**	*leptin receptor overlapping transcript*		**LRPPRC**	*leucine-rich pentatricopeptide repeat containing*	
**PRKAB2**	*protein kinase, AMP-activated, beta 2 non-catalytic subunit*		**KIAA1586**	*KIAA1586*	
**C10orf67**	*chromosome 10 open reading frame 67*		**THAP5**	*THAP domain containing 5*	
**RNF146**	*ring finger protein 146*		**KMT2A**	*lysine (K)-specific methyltransferase 2A*	
**FN1**	*fibronectin 1*		**METRNL**	*meteorin, glial cell differentiation regulator-like*	
**S1PR5**	*sphingosine-1-phosphate receptor 5*		**UACA**	*uveal autoantigen with coiled-coil domains and ankyrin repeats*	
**GRHL2**	*grainyhead-like 2 (Drosophila)*		**TRIM68**	*tripartite motif containing 68*	
**LINC00632**	*long intergenic non-protein coding RNA 632*		**OLR1**	*oxidized low density lipoprotein (lectin-like) receptor 1*	
**FOXK1**	*forkhead box K1*		**RASGRF1**	*Ras protein-specific guanine nucleotide-releasing factor 1*	
**DUSP1**	*dual specificity phosphatase 1*		**GOLGA6L6**	*golgin A6 family-like 6*	
**EMP2**	*epithelial membrane protein 2*		**SCRT2**	*scratch homolog 2, zinc finger protein (Drosophila)*	
**BIN1**	*bridging integrator 1*		**TEF**	*thyrotrophic embryonic factor*	
**TSC1**	*tuberous sclerosis 1*		**B3GAT1**	*beta-1,3-glucuronyltransferase 1 (glucuronosyltransferase P)*	
**STAMBP**	*STAM binding protein*		**TRIM26**	*tripartite motif containing 26*	
**AGTRAP**	*angiotensin II receptor-associated protein*		**MSL1**	*male-specific lethal 1 homolog (Drosophila)*	
**SH3BP5L**	*SH3-binding domain protein 5-like*		**ITPRIP**	*inositol 1,4,5-trisphosphate receptor interacting protein*	
**C14orf119**	*chromosome 14 open reading frame 119*		**AGO4**	*argonaute RISC catalytic component 4*	
**PURG**	*purine-rich element binding protein G*		**CDC42BPA**	*CDC42 binding protein kinase alpha (DMPK-like)*	
**GNAS**	*GNAS complex locus*	✓	**POLH**	*polymerase (DNA directed), eta*	
**DRAXIN**	*dorsal inhibitory axon guidance protein*		**TMEM200C**	*transmembrane protein 200C*	
**FGD1**	*FYVE, RhoGEF and PH domain containing 1*		**ZC3H13**	*zinc finger CCCH-type containing 13*	

**RBM5**	*RNA binding motif protein 5*		**FAM175A**	*family with sequence similarity 175, member A*	
**GOLGA7B**	*golgin A7 family, member B*		**TMEM235**	*transmembrane protein 235*	
**BACH2**	*BTB and CNC homology 1, basic leucine zipper transcription factor 2*		**BIN3**	*bridging integrator 3*	
**AC004899.1**	*Uncharacterized protein*		**GMEB2**	*glucocorticoid modulatory element binding protein 2*	
**DERL1**	*derlin 1*		**TTYH2**	*tweety family member 2*	
**KIAA1522**	*KIAA1522*		**WDFY3**	*WD repeat and FYVE domain containing 3*	
**GSTT1**	*glutathione S-transferase theta 1*		**SREBF1**	*sterol regulatory element binding transcription factor 1*	
**NFS1**	*NFS1 cysteine desulfurase*		**MDM2**	*MDM2 oncogene, E3 ubiquitin protein ligase*	
**LECT2**	*leukocyte cell-derived chemotaxin 2*		**PDK3**	*pyruvate dehydrogenase kinase, isozyme 3*	
**FOSB**	*FBJ murine osteosarcoma viral oncogene homolog B*		**PAPD7**	*PAP associated domain containing 7*	
**GSG1**	*germ cell associated 1*		**TIA1**	*TIA1 cytotoxic granule-associated RNA binding protein*	
**KCNS3**	*potassium voltage-gated channel, delayed-rectifier, subfamily S, member 3*		**PLXNA4**	*plexin A4*	
**CDK18**	*cyclin-dependent kinase 18*		**SKIDA1**	*SKI/DACH domain containing 1*	
**C5orf45**	*chromosome 5 open reading frame 45*		**AMOTL1**	*angiomotin like 1*	
**SLITRK6**	*SLIT and NTRK-like family, member 6*		**MAPKAPK3**	*mitogen-activated protein kinase-activated protein kinase 3*	
**SLC11A1**	*solute carrier family 11 (proton-coupled divalent metal ion transporter), member 1*		**PROSER2**	*proline and serine-rich protein 2*	
**CTD-2162K18.4**	*Uncharacterized protein*		**SMPD3**	*sphingomyelin phosphodiesterase 3, neutral membrane (neutral sphingomyelinase II)*	
**NAT8L**	*N-acetyltransferase 8-like (GCN5-related, putative)*		**VEGFA**	*vascular endothelial growth factor A*	
**SH3BGR**	*SH3 domain binding glutamic acid-rich protein*		**XRN1**	*5′-3′ exoribonuclease 1*	
**KRTAP5-5**	*keratin associated protein 5-5*		**TRAK2**	*trafficking protein, kinesin binding 2*	
**TSEN34**	*TSEN34 tRNA splicing endonuclease subunit*		**TAPBP**	*TAP binding protein (tapasin)*	
**PCDH1**	*protocadherin 1*		**LAMP3**	*lysosomal-associated membrane protein 3*	

**C15orf37**	*chromosome 15 open reading frame 37*		**SNX9**	*sorting nexin 9*	
**ERLIN2**	*ER lipid raft associated 2*		**NDUFA7**	*NADH dehydrogenase (ubiquinone) 1 alpha subcomplex, 7, 14.5kDa*	✓
**NRGN**	*neurogranin (protein kinase C substrate, RC3)*		**CLUH**	*clustered mitochondria (cluA/CLU1) homolog*	
**NMNAT3**	*nicotinamide nucleotide adenylyltransferase 3*		**ZNF618**	*zinc finger protein 618*	
**BTK**	*Bruton agammaglobulinemia tyrosine kinase*		**DENND6B**	*DENN/MADD domain containing 6B*	
**DNAJC30**	*DnaJ (Hsp40) homolog, subfamily C, member 30*		**FAHD2A**	*fumarylacetoacetate hydrolase domain containing 2A*	
**CD40**	*CD40 molecule, TNF receptor superfamily member 5*		**CBX6**	*chromobox homolog 6*	
**SEPT4**	*septin 4*		**ADCY9**	*adenylate cyclase 9*	
**VSTM2L**	*V-set and transmembrane domain containing 2 like*		**SMURF1**	*SMAD specific E3 ubiquitin protein ligase 1*	
**FUT4**	*fucosyltransferase 4 (alpha (1,3) fucosyltransferase, myeloid-specific)*		**VPS8**	*vacuolar protein sorting 8 homolog (S. cerevisiae)*	
**FAM57A**	*family with sequence similarity 57, member A*		**UBIAD1**	*UbiA prenyltransferase domain containing 1*	
**SCEL**	*sciellin*		**LMTK2**	*lemur tyrosine kinase 2*	
**SSRP1**	*structure specific recognition protein 1*		**SERPINB1**	*serpin peptidase inhibitor, clade B (ovalbumin), member 1*	
**SEC22C**	*SEC22 vesicle trafficking protein homolog C (S. cerevisiae)*		**PIRT**	*phosphoinositide-interacting regulator of transient receptor potential channels*	
**RBAK**	*RB-associated KRAB zinc finger*		**USHBP1**	*Usher syndrome 1C binding protein 1*	
**NETO1**	*neuropilin (NRP) and tolloid (TLL)-like 1*		**GALNT3**	*UDP-N-acetyl-alpha-D-galactosamine:polypeptide N-acetylgalactosaminyltransferase 3 (GalNAc-T3)*	
**KCTD5**	*potassium channel tetramerization domain containing 5*		**CTDSPL2**	*CTD (carboxy-terminal domain, RNA polymerase II, polypeptide A) small phosphatase like 2*	
**UBE2G2**	*ubiquitin-conjugating enzyme E2G 2*		**LHPP**	*phospholysine phosphohistidine inorganic pyrophosphate phosphatase*	
**MGAT5B**	*mannosyl (alpha-1,6-)-glycoprotein beta-1,6-N-acetyl-glucosaminyltransferase, isozyme B*		**NDST4**	*N-deacetylase/N-sulfotransferase (heparan glucosaminyl) 4*	
**REXO1L1**	*REX1, RNA exonuclease 1 homolog (S. cerevisiae)-like 1*		**PNPLA3**	*patatin-like phospholipase domain containing 3*	
**RAB36**	*RAB36, member RAS oncogene family*		**CBX1**	*chromobox homolog 1*	

**ZNF394**	*zinc finger protein 394*		**MED22**	*mediator complex subunit 22*	
**C6**	*complement component 6*		**GPM6B**	*glycoprotein M6B*	
**WDR25**	*WD repeat domain 25*		**SLC7A6**	*solute carrier family 7 (amino acid transporter light chain, y+L system), member 6*	
**GTF3C5**	*general transcription factor IIIC, polypeptide 5, 63kDa*		**DPY19L3**	*dpy-19-like 3 (C. elegans)*	
**IMPAD1**	*inositol monophosphatase domain containing 1*		**KIF3A**	*kinesin family member 3A*	
**C10orf53**	*chromosome 10 open reading frame 53*		**CHST3**	*carbohydrate (chondroitin 6) sulfotransferase 3*	
**MT-ND5**	*mitochondrially encoded NADH dehydrogenase 5*		**PLD5**	*phospholipase D family, member 5*	
**TMEM174**	*transmembrane protein 174*		**AP5B1**	*adaptor-related protein complex 5, beta 1 subunit*	
**ZNF814**	*zinc finger protein 814*		**FAM168B**	*family with sequence similarity 168, member B*	
**MSANTD1**	*Myb/SANT-like DNA-binding domain containing 1*		**IGF2R**	*insulin-like growth factor 2 receptor*	
**SHF**	*Src homology 2 domain containing F*		**ZNF43**	*zinc finger protein 43*	
**GYPC**	*glycophorin C (Gerbich blood group)*		**TMEM259**	*transmembrane protein 259*	
**ENTPD3**	*ectonucleoside triphosphate diphosphohydrolase 3*		**MPV17L2**	*MPV17 mitochondrial membrane protein-like 2*	
**T**	*T, brachyury homolog (mouse)*		**RNF125**	*ring finger protein 125, E3 ubiquitin protein ligase*	
**GDAP1**	*ganglioside induced differentiation associated protein 1*		**PTPN14**	*protein tyrosine phosphatase, non-receptor type 14*	
**OLIG1**	*oligodendrocyte transcription factor 1*		**QKI**	*QKI, KH domain containing, RNA binding*	
**ADI1**	*acireductone dioxygenase 1*		**SLC44A2**	*solute carrier family 44 (choline transporter), member 2*	
**ZBTB9**	*zinc finger and BTB domain containing 9*		**ZNF490**	*zinc finger protein 490*	
**FAM27E2**	*family with sequence similarity 27, member E2*		**TNKS**	*tankyrase, TRF1-interacting ankyrin-related ADP-ribose polymerase*	
**EML3**	*echinoderm microtubule associated protein like 3*		**SP6**	*Sp6 transcription factor*	
**ACADVL**	*acyl-CoA dehydrogenase, very long chain*		**N4BP1**	*NEDD4 binding protein 1*	
**AC079354.1**	*uncharacterized protein KIAA2012*		**ADD2**	*adducin 2 (beta)*	

**FAM27E1**	*family with sequence similarity 27, member E1*		**RIF1**	*RAP1 interacting factor homolog (yeast)*	
**AC112693.2**			**TLE3**	*transducin-like enhancer of split 3 (E(sp1) homolog, Drosophila)*	
**FAM27E3**	*family with sequence similarity 27, member E3*		**PDCD11**	*programmed cell death 11*	
**HIST1H3G**	*histone cluster 1, H3g*		**KLHL6**	*kelch-like family member 6*	
**C7orf50**	*chromosome 7 open reading frame 50*		**POLM**	*polymerase (DNA directed), mu*	
**INHBE**	*inhibin, beta E*		**KBTBD11**	*kelch repeat and BTB (POZ) domain containing 11*	
**ENPP5**	*ectonucleotide pyrophosphatase/phosphodiesterase 5 (putative)*		**HECTD3**	*HECT domain containing E3 ubiquitin protein ligase 3*	
**ZKSCAN1**	*zinc finger with KRAB and SCAN domains 1*		**PLEKHG2**	*pleckstrin homology domain containing, family G (with RhoGef domain) member 2*	
**COL6A1**	*collagen, type VI, alpha 1*		**PHF12**	*PHD finger protein 12*	
**PLIN5**	*perilipin 5*		**CDK19**	*cyclin-dependent kinase 19*	
**CXCR5**	*chemokine (C-X-C motif) receptor 5*		**FAM118A**	*family with sequence similarity 118, member A*	
**BHLHE40**	*basic helix-loop-helix family, member e40*		**SLC30A4**	*solute carrier family 30 (zinc transporter), member 4*	
**HLA-DPA1**	*major histocompatibility complex, class II, DP alpha 1*		**PDE12**	*phosphodiesterase 12*	
**BRSK2**	*BR serine/threonine kinase 2*		**EIF2AK2**	*eukaryotic translation initiation factor 2-alpha kinase 2*	
**FAM192A**	*family with sequence similarity 192, member A*		**BMPR1A**	*bone morphogenetic protein receptor, type IA*	
**GREM2**	*gremlin 2, DAN family BMP antagonist*		**HIPK2**	*homeodomain interacting protein kinase 2*	
**PRKCG**	*protein kinase C, gamma*		**TRIM45**	*tripartite motif containing 45*	
**MECP2**	*methyl CpG binding protein 2 (Rett syndrome)*	✓	**MTUS2**	*microtubule associated tumor suppressor candidate 2*	
**CSF3**	*colony stimulating factor 3 (granulocyte)*		**SIPA1L3**	*signal-induced proliferation-associated 1 like 3*	
**SEPT5**	*septin 5*		**HIST1H2AH**	*histone cluster 1, H2ah*	
**RAB11A**	*RAB11A, member RAS oncogene family*		**CLPB**	*ClpB caseinolytic peptidase B homolog (E. coli)*	
**PSTK**	*phosphoseryl-tRNA kinase*		**RFESD**	*Rieske (Fe-S) domain containing*	

**ZFP36L1**	*ZFP36 ring finger protein-like 1*		**ITPK1**	*inositol-tetrakisphosphate 1-kinase*	
**HTR3C**	*5-hydroxytryptamine (serotonin) receptor 3C, ionotropic*		**SLC25A53**	*solute carrier family 25, member 53*	
**ABHD17B**	*abhydrolase domain containing 17B*		**CMTM4**	*CKLF-like MARVEL transmembrane domain containing 4*	
**SLC35C1**	*solute carrier family 35 (GDP-fucose transporter), member C1*		**ADIRF**	*adipogenesis regulatory factor*	
**FOXN4**	*forkhead box N4*		**CAMK2D**	*calcium/calmodulin-dependent protein kinase II delta*	
**PFKFB4**	*6-phosphofructo-2-kinase/fructose-2,6-biphosphatase 4*		**MYPN**	*myopalladin*	
**F9**	*coagulation factor IX*		**NTNG2**	*netrin G2*	
**C16orf98**	*chromosome 16 open reading frame 98*		**EMC8**	*ER membrane protein complex subunit 8*	
**AC110619.2**	*Uncharacterized protein*		**PAQR4**	*progestin and adipoQ receptor family member IV*	
**FOXD4**	*forkhead box D4*		**SOX5**	*SRY (sex determining region Y)-box 5*	
**CSDE1**	*cold shock domain containing E1, RNA-binding*		**YWHAZ**	*tyrosine 3-monooxygenase/tryptophan 5-monooxygenase activation protein, zeta polypeptide*	
**FAM163A**	*family with sequence similarity 163, member A*		**TBL1X**	*transducin (beta)-like 1X-linked*	
**SLC9C2**	*solute carrier family 9, member C2 (putative)*		**NAT16**	*N-acetyltransferase 16 (GCN5-related, putative)*	
**MID1**	*midline 1 (Opitz/BBB syndrome)*		**NKX6-2**	*NK6 homeobox 2*	
**NOP14**	*NOP14 nucleolar protein*		**MCM8**	*minichromosome maintenance complex component 8*	
**ZSCAN2**	*zinc finger and SCAN domain containing 2*		**GDF7**	*growth differentiation factor 7*	
**FAM154A**	*family with sequence similarity 154, member A*		**UBA2**	*ubiquitin-like modifier activating enzyme 2*	
**SLC20A2**	*solute carrier family 20 (phosphate transporter), member 2*		**HIST1H2BD**	*histone cluster 1, H2bd*	
**CSNK2A2**	*casein kinase 2, alpha prime polypeptide*		**FAM206A**	*family with sequence similarity 206, member A*	
**TENM3**	*teneurin transmembrane protein 3*		**CXADR**	*coxsackie virus and adenovirus receptor*	
**ARHGAP35**	*Rho GTPase activating protein 35*		**SEPN1**	*selenoprotein N, 1*	
**DLGAP3**	*discs, large (Drosophila) homolog-associated protein 3*		**ARHGAP39**	*Rho GTPase activating protein 39*	

**MEOX1**	*mesenchyme homeobox 1*		**KDELR2**	*KDEL (Lys-Asp-Glu-Leu) endoplasmic reticulum protein retention receptor 2*	
**TOR1A**	*torsin family 1, member A (torsin A)*		**EPB41L1**	*erythrocyte membrane protein band 4.1-like 1*	
**CD1E**	*CD1e molecule*		**DHCR7**	*7-dehydrocholesterol reductase*	
**MICB**	*MHC class I polypeptide-related sequence B*		**HMCES**	*5-hydroxymethylcytosine (hmC) binding, ES cell-specific*	
**FAM46B**	*family with sequence similarity 46, member B*		**ARRB1**	*arrestin, beta 1*	
**PPAP2B**	*phosphatidic acid phosphatase type 2B*		**CMKLR1**	*chemokine-like receptor 1*	
**RPL13A**	*ribosomal protein L13a*		**DNMT3A**	*DNA (cytosine-5-)-methyltransferase 3 alpha*	
**CORO6**	*coronin 6*		**PSD2**	*pleckstrin and Sec7 domain containing 2*	
**CYB561D1**	*cytochrome b561 family, member D1*		**USP20**	*ubiquitin specific peptidase 20*	
**KCNC1**	*potassium voltage-gated channel, Shaw-related subfamily, member 1*		**UNC13A**	*unc-13 homolog A (C. elegans)*	
**SPRED3**	*sprouty-related, EVH1 domain containing 3*		**DCAF7**	*DDB1 and CUL4 associated factor 7*	
**RNF44**	*ring finger protein 44*		**ABI2**	*abl-interactor 2*	
**PALM**	*paralemmin*		**NEDD4L**	*neural precursor cell expressed, developmentally down-regulated 4-like, E3 ubiquitin protein ligase*	
**ADRB2**	*adrenoceptor beta 2, surface*		**PPP1R12B**	*protein phosphatase 1, regulatory subunit 12B*	
**ADAT1**	*adenosine deaminase, tRNA-specific 1*		**CBX8**	*chromobox homolog 8*	
**PDCD1**	*programmed cell death 1*		**CENPN**	*centromere protein N*	
**CLGN**	*calmegin*		**SIAE**	*sialic acid acetylesterase*	
**SLC10A1**	*solute carrier family 10 (sodium/bile acid cotransporter), member 1*		**CEBPD**	*CCAAT/enhancer binding protein (C/EBP), delta*	
**KLF4**	*Kruppel-like factor 4 (gut)*		**HNRNPUL2**	*heterogeneous nuclear ribonucleoprotein U-like 2*	
**HOXA13**	*homeobox A13*		**PEX26**	*peroxisomal biogenesis factor 26*	
**IL23R**	*interleukin 23 receptor*		**HOXD11**	*homeobox D11*	
**SPN**	*sialophorin*		**CLUAP1**	*clusterin associated protein 1*	
**PSAPL1**	*prosaposin-like 1 (gene/pseudogene)*		**MED7**	*mediator complex subunit 7*	

**TMEM158**	*transmembrane protein 158 (gene/pseudogene)*		**UTP15**	*UTP15, U3 small nucleolar ribonucleoprotein, homolog (S. cerevisiae)*	
**POU2F3**	*POU class 2 homeobox 3*		**NUDT1**	*nudix (nucleoside diphosphate linked moiety X)-type motif 1*	
**B3GNT6**	*UDP-GlcNAc:betaGal beta-1,3-N-acetylglucosaminyltransferase 6 (core 3 synthase)*		**SYT7**	*synaptotagmin VII*	
**ZNF282**	*zinc finger protein 282*		**HIST1H2AI**	*histone cluster 1, H2ai*	
**ZFP41**	*ZFP41 zinc finger protein*		**CYTH2**	*cytohesin 2*	
**NFATC2**	*nuclear factor of activated T-cells, cytoplasmic, calcineurin-dependent 2*		**COLGALT2**	*collagen beta(1-O)galactosyltransferase 2*	
**COL19A1**	*collagen, type XIX, alpha 1*		**TMEM251**	*transmembrane protein 251*	
**CASP14**	*caspase 14, apoptosis-related cysteine peptidase*		**ZNF701**	*zinc finger protein 701*	
**SEC31B**	*SEC31 homolog B (S. cerevisiae)*		**RPL32**	*ribosomal protein L32*	
**GRAMD4**	*GRAM domain containing 4*		**TIMM8A**	*translocase of inner mitochondrial membrane 8 homolog A (yeast)*	
**USP4**	*ubiquitin specific peptidase 4 (proto-oncogene)*		**BDH1**	*3-hydroxybutyrate dehydrogenase, type 1*	
**SSU72**	*SSU72 RNA polymerase II CTD phosphatase homolog (S. cerevisiae)*		**PTCD1**	*pentatricopeptide repeat domain 1*	
**SH3BP4**	*SH3-domain binding protein 4*		**FANCA**	*Fanconi anemia, complementation group A*	
**HES7**	*hairy and enhancer of split 7 (Drosophila)*		**FXN**	*frataxin*	
**B4GALT5**	*UDP-Gal:betaGlcNAc beta 1,4-galactosyltransferase, polypeptide 5*		**UBXN7**	*UBX domain protein 7*	
**LASP1**	*LIM and SH3 protein 1*		**DNAJC18**	*DnaJ (Hsp40) homolog, subfamily C, member 18*	
**CELF6**	*CUGBP, Elav-like family member 6*		**ZNF573**	*zinc finger protein 573*	
**HOXC13**	*homeobox C13*		**KIF26B**	*kinesin family member 26B*	
**PRPH2**	*peripherin 2 (retinal degeneration, slow)*		**TMEM242**	*transmembrane protein 242*	
**UBAP2**	*ubiquitin associated protein 2*		**INPP4A**	*inositol polyphosphate-4-phosphatase, type I, 107kDa*	
**MYO18B**	*myosin XVIIIB*		**TMEM120B**	*transmembrane protein 120B*	
**MCM3AP**	*minichromosome maintenance complex component 3 associated protein*		**NRG2**	*neuregulin 2*	

**SNX33**	*sorting nexin 33*		**PSMG1**	*proteasome (prosome, macropain) assembly chaperone 1*	
**FAM50B**	*family with sequence similarity 50, member B*		**AUNIP**	*aurora kinase A and ninein interacting protein*	
**IPP**	*intracisternal A particle-promoted polypeptide*		**EXPH5**	*exophilin 5*	
**TOR2A**	*torsin family 2, member A*		**MNAT1**	*MNAT CDK-activating kinase assembly factor 1*	
**PPARGC1B**	*peroxisome proliferator-activated receptor gamma, coactivator 1 beta*		**SH3PXD2A**	*SH3 and PX domains 2A*	
**PTPRQ**	*protein tyrosine phosphatase, receptor type, Q*		**ZDHHC24**	*zinc finger, DHHC-type containing 24*	
**RSPO4**	*R-spondin 4*		**PARVA**	*parvin, alpha*	
**GFRAL**	*GDNF family receptor alpha like*		**VLDLR**	*very low density lipoprotein receptor*	
**CYS1**	*cystin 1*		**TYRO3**	*TYRO3 protein tyrosine kinase*	
**FIBIN**	*fin bud initiation factor homolog (zebrafish)*		**PSME3**	*proteasome (prosome, macropain) activator subunit 3 (PA28 gamma; Ki)*	✓
**ADM**	*adrenomedullin*		**ATP1B2**	*ATPase, Na+/K+ transporting, beta 2 polypeptide*	
**EID2B**	*EP300 interacting inhibitor of differentiation 2B*		**ZNF264**	*zinc finger protein 264*	
**PCDHGA11**	*protocadherin gamma subfamily A, 11*		**MICA**	*MHC class I polypeptide-related sequence A*	✓
**PTX4**	*pentraxin 4, long*		**TPRG1**	*tumor protein p63 regulated 1*	
**FAM83B**	*family with sequence similarity 83, member B*		**ZNF805**	*zinc finger protein 805*	
**SESN2**	*sestrin 2*	✓	**CREB5**	*cAMP responsive element binding protein 5*	
**TBX10**	*T-box 10*		**ASPHD2**	*aspartate beta-hydroxylase domain containing 2*	
**DUSP16**	*dual specificity phosphatase 16*		**ATP6V1C1**	*ATPase, H+ transporting, lysosomal 42kDa, V1 subunit C1*	
**ABCA10**	*ATP-binding cassette, sub-family A (ABC1), member 10*		**GPC1**	*glypican 1*	
**GPR124**	*G protein-coupled receptor 124*		**ASB1**	*ankyrin repeat and SOCS box containing 1*	
**RAP2A**	*RAP2A, member of RAS oncogene family*		**FNBP1**	*formin binding protein 1*	
**ZNF260**	*zinc finger protein 260*	✓	**THSD4**	*thrombospondin, type I, domain containing 4*	
**ZNF595**	*zinc finger protein 595*		**TENM4**	*teneurin transmembrane protein 4*	

**ZNF804B**	*zinc finger protein 804B*		**MBNL3**	*muscleblind-like splicing regulator 3*	
**PRKRIP1**	*PRKR interacting protein 1 (IL11 inducible)*		**FANCM**	*Fanconi anemia, complementation group M*	
**TTC7A**	*tetratricopeptide repeat domain 7A*		**REPS2**	*RALBP1 associated Eps domain containing 2*	
**POMK**	*protein-O-mannose kinase*		**DYNC1LI2**	*dynein, cytoplasmic 1, light intermediate chain 2*	
**TOR4A**	*torsin family 4, member A*		**ICOSLG**	*inducible T-cell co-stimulator ligand*	
**TM4SF20**	*transmembrane 4 L six family member 20*		**WDFY2**	*WD repeat and FYVE domain containing 2*	
**TNFRSF10A**	*tumor necrosis factor receptor superfamily, member 10a*		**XPNPEP3**	*X-prolyl aminopeptidase (aminopeptidase P) 3, putative*	
**LPIN1**	*lipin 1*		**BCAR1**	*breast cancer anti-estrogen resistance 1*	
**ATG16L1**	*autophagy related 16-like 1 (S. cerevisiae)*		**FAM105B**	*family with sequence similarity 105, member B*	
**B4GALT2**	*UDP-Gal:betaGlcNAc beta 1,4-galactosyltransferase, polypeptide 2*		**ZNF555**	*zinc finger protein 555*	
**TSPAN18**	*tetraspanin 18*		**HSD17B12**	*hydroxysteroid (17-beta) dehydrogenase 12*	
**COL9A1**	*collagen, type IX, alpha 1*		**ENOSF1**	*enolase superfamily member 1*	
**VAMP8**	*vesicle-associated membrane protein 8*		**TFDP2**	*transcription factor Dp-2 (E2F dimerization partner 2)*	
**SEMA6C**	*sema domain, transmembrane domain (TM), and cytoplasmic domain, (semaphorin) 6C*		**RAB11FIP4**	*RAB11 family interacting protein 4 (class II)*	
**ENPP7**	*ectonucleotide pyrophosphatase/phosphodiesterase 7*		**HSD17B8**	*hydroxysteroid (17-beta) dehydrogenase 8*	
**C9orf91**	*chromosome 9 open reading frame 91*		**TXNDC17**	*thioredoxin domain containing 17*	
**ENTPD1**	*ectonucleoside triphosphate diphosphohydrolase 1*		**KLHL30**	*kelch-like family member 30*	
**LIX1L**	*Lix1 homolog (mouse)-like*		**LHX6**	*LIM homeobox 6*	
**PCDHGA12**	*protocadherin gamma subfamily A, 12*		**AJAP1**	*adherens junctions associated protein 1*	
**PCDHGA1**	*protocadherin gamma subfamily A, 1*		**PTPRF**	*protein tyrosine phosphatase, receptor type, F*	✓
**PCDHGA2**	*protocadherin gamma subfamily A, 2*		**ZNF766**	*zinc finger protein 766*	
**MTMR10**	*myotubularin related protein 10*		**NSL1**	*NSL1, MIS12 kinetochore complex component*	

**WNT7B**	*wingless-type MMTV integration site family, member 7B*		**C11orf48**	*chromosome 11 open reading frame 48*	
**LIN28B**	*lin-28 homolog B (C. elegans)*		**ZNRF3**	*zinc and ring finger 3*	
**FAM126B**	*family with sequence similarity 126, member B*		**ANXA11**	*annexin A11*	
**IRF4**	*interferon regulatory factor 4*		**NIPA1**	*non imprinted in Prader-Willi/Angelman syndrome 1*	
**C10orf54**	*chromosome 10 open reading frame 54*		**DDN**	*dendrin*	
**FAM86C1**	*family with sequence similarity 86, member C1*		**HECW2**	*HECT, C2 and WW domain containing E3 ubiquitin protein ligase 2*	
**STARD8**	*StAR-related lipid transfer (START) domain containing 8*		**DMXL1**	*Dmx-like 1*	
**SPSB1**	*splA/ryanodine receptor domain and SOCS box containing 1*	✓	**ACTR1A**	*ARP1 actin-related protein 1 homolog A, centractin alpha (yeast)*	
**LSM11**	*LSM11, U7 small nuclear RNA associated*		**C5orf63**	*chromosome 5 open reading frame 63*	
**ABHD17A**	*abhydrolase domain containing 17A*		**TTLL1**	*tubulin tyrosine ligase-like family, member 1*	
**GEMIN7**	*gem (nuclear organelle) associated protein 7*		**C6orf211**	*chromosome 6 open reading frame 211*	
**GPR20**	*G protein-coupled receptor 20*		**RBM28**	*RNA binding motif protein 28*	
**MFAP4**	*microfibrillar-associated protein 4*		**SSPN**	*sarcospan*	
**GAK**	*cyclin G associated kinase*		**LPAR2**	*lysophosphatidic acid receptor 2*	
**CACNG7**	*calcium channel, voltage-dependent, gamma subunit 7*		**SLC25A16**	*solute carrier family 25 (mitochondrial carrier; Graves disease autoantigen), member 16*	
**OPN4**	*opsin 4*		**GLUD1**	*glutamate dehydrogenase 1*	
**CAMKK2**	*calcium/calmodulin-dependent protein kinase kinase 2, beta*		**GFER**	*growth factor, augmenter of liver regeneration*	
**BICD2**	*bicaudal D homolog 2 (Drosophila)*		**CENPA**	*centromere protein A*	
**PRRC2B**	*proline-rich coiled-coil 2B*		**C17orf85**	*chromosome 17 open reading frame 85*	
**HOXA10**	*homeobox A10*		**RAB3C**	*RAB3C, member RAS oncogene family*	
**UBTD1**	*ubiquitin domain containing 1*		**ISL2**	*ISL LIM homeobox 2*	
**BCAS3**	*breast carcinoma amplified sequence 3*		**MRPL46**	*mitochondrial ribosomal protein L46*	
**ZNF28**	*zinc finger protein 28*	✓	**PHB2**	*prohibitin 2*	

**N4BP3**	*NEDD4 binding protein 3*		**TMEM18**	*transmembrane protein 18*	
**SLC16A13**	*solute carrier family 16, member 13*		**CTIF**	*CBP80/20-dependent translation initiation factor*	
**QPCTL**	*glutaminyl-peptide cyclotransferase-like*		**SMNDC1**	*survival motor neuron domain containing 1*	
**PLEK**	*pleckstrin*		**CENPP**	*centromere protein P*	
**NOS1AP**	*nitric oxide synthase 1 (neuronal) adaptor protein*		**HMX1**	*H6 family homeobox 1*	
**PDE4B**	*phosphodiesterase 4B, cAMP-specific*		**RNF20**	*ring finger protein 20, E3 ubiquitin protein ligase*	
**ARHGAP19**	*Rho GTPase activating protein 19*		**KLHL18**	*kelch-like family member 18*	
**POMGNT1**	*protein O-linked mannose N-acetylglucosaminyltransferase 1 (beta 1,2-)*		**THUMPD3**	*THUMP domain containing 3*	
**VSTM5**	*V-set and transmembrane domain containing 5*		**KDM5A**	*lysine (K)-specific demethylase 5A*	
**GABRA6**	*gamma-aminobutyric acid (GABA) A receptor, alpha 6*		**KIAA0141**	*KIAA0141*	
**APLN**	*apelin*		**MINPP1**	*multiple inositol-polyphosphate phosphatase 1*	
**CCNJL**	*cyclin J-like*		**C18orf21**	*chromosome 18 open reading frame 21*	
**CCL22**	*chemokine (C-C motif) ligand 22*		**RPP14**	*ribonuclease P/MRP 14kDa subunit*	
**FAM129B**	*family with sequence similarity 129, member B*		**PLCG2**	*phospholipase C, gamma 2 (phosphatidylinositol-specific)*	
**RNF150**	*ring finger protein 150*		**TLN2**	*talin 2*	
**C5orf24**	*chromosome 5 open reading frame 24*		**MXI1**	*MAX interactor 1, dimerization protein*	
**SLC6A3**	*solute carrier family 6 (neurotransmitter transporter), member 3*		**PLEKHA3**	*pleckstrin homology domain containing, family A (phosphoinositide binding specific) member 3*	
**ZNF878**	*zinc finger protein 878*		**MED28**	*mediator complex subunit 28*	
**LRRC20**	*leucine rich repeat containing 20*		**ARSG**	*arylsulfatase G*	
**HES2**	*hairy and enhancer of split 2 (Drosophila)*		**DSC3**	*desmocollin 3*	
**GBA**	*glucosidase, beta, acid*		**MTHFD2**	*methylenetetrahydrofolate dehydrogenase (NADP+ dependent) 2, methenyltetrahydrofolate cyclohydrolase*	✓
**ZFP69B**	*ZFP69 zinc finger protein B*		**MRPL42**	*mitochondrial ribosomal protein L42*	

**UMODL1**	*uromodulin-like 1*		**KCNMB4**	*potassium large conductance calcium-activated channel, subfamily M, beta member 4*	
**FBLIM1**	*filamin binding LIM protein 1*		**PTGR2**	*prostaglandin reductase 2*	
**ZNF101**	*zinc finger protein 101*		**CLK4**	*CDC-like kinase 4*	
**DKFZP434H0512**	*Protein LOC100506667; Putative uncharacterized protein DKFZp434H0512*		**TFIP11**	*tuftelin interacting protein 11*	✓
**KRT80**	*keratin 80*		**SLC25A12**	*solute carrier family 25 (aspartate/glutamate carrier), member 12*	
**SIM1**	*single-minded homolog 1 (Drosophila)*		**PIK3CA**	*phosphatidylinositol-4,5-bisphosphate 3-kinase, catalytic subunit alpha*	
**AC132872.2**	*CDNA FLJ27256 fis, clone SYN09689; Uncharacterized protein*		**ZNF397**	*zinc finger protein 397*	
**APOLD1**	*apolipoprotein L domain containing 1*		**PRIM1**	*primase, DNA, polypeptide 1 (49kDa)*	
**PAX7**	*paired box 7*		**ANXA6**	*annexin A6*	
**VWC2L**	*von Willebrand factor C domain containing protein 2-like*		**OIP5**	*Opa interacting protein 5*	
**ITGA11**	*integrin, alpha 11*		**NCAPG2**	*non-SMC condensin II complex, subunit G2*	✓
**SLC35F1**	*solute carrier family 35, member F1*		**GPR39**	*G protein-coupled receptor 39*	
**COL5A1**	*collagen, type V, alpha 1*		**SMARCC1**	*SWI/SNF related, matrix associated, actin dependent regulator of chromatin, subfamily c, member 1*	
**VWA1**	*von Willebrand factor A domain containing 1*		**NIF3L1**	*NIF3 NGG1 interacting factor 3-like 1 (S. cerevisiae)*	
**MINK1**	*misshapen-like kinase 1*		**C1orf35**	*chromosome 1 open reading frame 35*	✓
**SLC12A8**	*solute carrier family 12, member 8*		**HNRNPA0**	*heterogeneous nuclear ribonucleoprotein A0*	
**TMEM52B**	*transmembrane protein 52B*		**C12orf5**	*chromosome 12 open reading frame 5*	
**KIAA1432**	*KIAA1432*		**ZBTB40**	*zinc finger and BTB domain containing 40*	
**PITX1**	*paired-like homeodomain 1*		**LMF1**	*lipase maturation factor 1*	
**ANGPTL1**	*angiopoietin-like 1*		**SERPING1**	*serpin peptidase inhibitor, clade G (C1 inhibitor), member 1*	
**CHRNA4**	*cholinergic receptor, nicotinic, alpha 4 (neuronal)*		**CCDC149**	*coiled-coil domain containing 149*	
**EXOSC10**	*exosome component 10*		**AKT1**	*v-akt murine thymoma viral oncogene homolog 1*	

**SHISA5**	*shisa family member 5*		**SPRYD4**	*SPRY domain containing 4*	
**DNAJC6**	*DnaJ (Hsp40) homolog, subfamily C, member 6*		**BTG1**	*B-cell translocation gene 1, anti-proliferative*	
**FBXO45**	*F-box protein 45*		**FOXK2**	*forkhead box K2*	
**PIM1**	*pim-1 oncogene*		**HIF1A**	*hypoxia inducible factor 1, alpha subunit (basic helix-loop-helix transcription factor)*	
**HECA**	*headcase homolog (Drosophila)*		**RANBP6**	*RAN binding protein 6*	
**ZNF275**	*zinc finger protein 275*		**ITGB5**	*integrin, beta 5*	
**ANKS1A**	*ankyrin repeat and sterile alpha motif domain containing 1A*		**HSPA13**	*heat shock protein 70kDa family, member 13*	
**DTL**	*denticleless E3 ubiquitin protein ligase homolog (Drosophila)*		**DYRK1A**	*dual-specificity tyrosine-(Y)-phosphorylation regulated kinase 1A*	
**PPFIA3**	*protein tyrosine phosphatase, receptor type, f polypeptide (PTPRF), interacting protein (liprin), alpha 3*		**FAS**	*Fas cell surface death receptor*	
**GCC1**	*GRIP and coiled-coil domain containing 1*		**MRPL36**	*mitochondrial ribosomal protein L36*	
**FSTL4**	*follistatin-like 4*		**PKIA**	*protein kinase (cAMP-dependent, catalytic) inhibitor alpha*	
**TNFRSF13C**	*tumor necrosis factor receptor superfamily, member 13C*		**CDK12**	*cyclin-dependent kinase 12*	
**HLA-DPB1**	*major histocompatibility complex, class II, DP beta 1*		**SERTAD1**	*SERTA domain containing 1*	
**SLC35E2B**	*solute carrier family 35, member E2B*		**CHAF1B**	*chromatin assembly factor 1, subunit B (p60)*	
**PCDH11Y**	*protocadherin 11 Y-linked*		**YTHDC1**	*YTH domain containing 1*	
**TAL2**	*T-cell acute lymphocytic leukemia 2*		**C11orf83**	*chromosome 11 open reading frame 83*	
**ADO**	*2-aminoethanethiol (cysteamine) dioxygenase*		**CEP63**	*centrosomal protein 63kDa*	
**KCNC3**	*potassium voltage-gated channel, Shaw-related subfamily, member 3*		**SURF2**	*surfeit 2*	
**ICA1L**	*islet cell autoantigen 1,69kDa-like*		**EMC2**	*ER membrane protein complex subunit 2*	✓
**PMEPA1**	*prostate transmembrane protein, androgen induced 1*		**RNF115**	*ring finger protein 115*	
**USP36**	*ubiquitin specific peptidase 36*		**CBX2**	*chromobox homolog 2*	
**RASL12**	*RAS-like, family 12*		**TMEM43**	*transmembrane protein 43*	

**ARHGEF18**	*Rho/Rac guanine nucleotide exchange factor (GEF) 18*		**PLXNA3**	*plexin A3*	
**DNAJC24**	*DnaJ (Hsp40) homolog, subfamily C, member 24*		**CENPI**	*centromere protein I*	
**CARNS1**	*carnosine synthase 1*		**AP5Z1**	*adaptor-related protein complex 5, zeta 1 subunit*	
**SCRN1**	*secernin 1*		**STX7**	*syntaxin 7*	
**SH3RF2**	*SH3 domain containing ring finger 2*		**GATA6**	*GATA binding protein 6*	✓
**SCUBE3**	*signal peptide, CUB domain, EGF-like 3*		**RAE1**	*ribonucleic acid export 1*	
**HAPLN4**	*hyaluronan and proteoglycan link protein 4*		**MAN2B2**	*mannosidase, alpha, class 2B, member 2*	
**IQCG**	*IQ motif containing G*		**CTC1**	*CTS telomere maintenance complex component 1*	
**ABLIM1**	*actin binding LIM protein 1*		**VPS18**	*vacuolar protein sorting 18 homolog (S. cerevisiae)*	
**NUDT15**	*nudix (nucleoside diphosphate linked moiety X)-type motif 15*		**WDR73**	*WD repeat domain 73*	✓
**CCDC180**	*coiled-coil domain containing 180*		**HERPUD1**	*homocysteine-inducible, endoplasmic reticulum stress-inducible, ubiquitin-like domain member 1*	
**PCDHGB1**	*protocadherin gamma subfamily B, 1*		**BDKRB2**	*bradykinin receptor B2*	
**LEMD2**	*LEM domain containing 2*		**NCKIPSD**	*NCK interacting protein with SH3 domain*	
**PCDHGB4**	*protocadherin gamma subfamily B, 4*		**DDI2**	*DNA-damage inducible 1 homolog 2 (S. cerevisiae)*	
**PCDHGA6**	*protocadherin gamma subfamily A, 6*		**PTDSS2**	*phosphatidylserine synthase 2*	✓
**PCDHGA8**	*protocadherin gamma subfamily A, 8*		**RPL23**	*ribosomal protein L23*	
**PCDHGA5**	*protocadherin gamma subfamily A, 5*		**GRSF1**	*G-rich RNA sequence binding factor 1*	
**PCDHGB3**	*protocadherin gamma subfamily B, 3*		**MRPS23**	*mitochondrial ribosomal protein S23*	
**PCDHGA7**	*protocadherin gamma subfamily A, 7*		**HIGD2A**	*HIG1 hypoxia inducible domain family, member 2A*	
**PCDHGC4**	*protocadherin gamma subfamily C, 4*		**SGSM2**	*small G protein signaling modulator 2*	
**PCDHGA3**	*protocadherin gamma subfamily A, 3*		**PHAX**	*phosphorylated adaptor for RNA export*	
**PCDHGB7**	*protocadherin gamma subfamily B, 7*		**NFYA**	*nuclear transcription factor Y, alpha*	
**PCDHGC5**	*protocadherin gamma subfamily C, 5*		**TCP10L**	*t-complex 10-like*	
**PCDHGA10**	*protocadherin gamma subfamily A, 10*		**IGFBP4**	*insulin-like growth factor binding protein 4*	

**PCDHGB2**	*protocadherin gamma subfamily B, 2*		**G6PC**	*glucose-6-phosphatase, catalytic subunit*	
**PCDHGC3**	*protocadherin gamma subfamily C, 3*		**TCIRG1**	*T-cell, immune regulator 1, ATPase, H+ transporting, lysosomal V0 subunit A3*	
**PCDHGB6**	*protocadherin gamma subfamily B, 6*		**PHF19**	*PHD finger protein 19*	
**PCDHGA9**	*protocadherin gamma subfamily A, 9*		**C17orf72**	*chromosome 17 open reading frame 72*	
**PCDHGA4**	*protocadherin gamma subfamily A, 4*		**CCS**	*copper chaperone for superoxide dismutase*	
**GLTSCR1**	*glioma tumor suppressor candidate region gene 1*		**CAPZB**	*capping protein (actin filament) muscle Z-line, beta*	
**MYOG**	*myogenin (myogenic factor 4)*		**C9orf173**	*chromosome 9 open reading frame 173*	
**BPTF**	*bromodomain PHD finger transcription factor*		**PDE9A**	*phosphodiesterase 9A*	
**ALKBH4**	*alkB, alkylation repair homolog 4 (E. coli)*		**MED23**	*mediator complex subunit 23*	
**WT1**	*Wilms tumor 1*		**VAV3**	*vav 3 guanine nucleotide exchange factor*	✓
**ATCAY**	*ataxia, cerebellar, Cayman type*		**SLC2A9**	*solute carrier family 2 (facilitated glucose transporter), member 9*	
**LITAF**	*lipopolysaccharide-induced TNF factor*		**OPN1MW**	*opsin 1 (cone pigments), medium-wave-sensitive*	
**MLLT1**	*myeloid/lymphoid or mixed-lineage leukemia (trithorax homolog, Drosophila); translocated to, 1*		**TRAF1**	*TNF receptor-associated factor 1*	
**ZNF263**	*zinc finger protein 263*		**OPN1MW2**	*opsin 1 (cone pigments), medium-wave-sensitive 2*	
**GPAM**	*glycerol-3-phosphate acyltransferase, mitochondrial*		**GRIN2A**	*glutamate receptor, ionotropic, N-methyl D-aspartate 2A*	
**MET**	*met proto-oncogene*		**GATSL2**	*GATS protein-like 2*	
**HOXB6**	*homeobox B6*		**RNF126**	*ring finger protein 126*	
**KCNJ16**	*potassium inwardly-rectifying channel, subfamily J, member 16*		**CLEC12B**	*C-type lectin domain family 12, member B*	
**PRIMA1**	*proline rich membrane anchor 1*		**CD226**	*CD226 molecule*	
**PBX1**	*pre-B-cell leukemia homeobox 1*		**RBX1**	*ring-box 1, E3 ubiquitin protein ligase*	
**USP28**	*ubiquitin specific peptidase 28*		**KCNC2**	*potassium voltage-gated channel, Shaw-related subfamily, member 2*	
**TRAF5**	*TNF receptor-associated factor 5*		**CCSER1**	*coiled-coil serine-rich protein 1*	

**SLC25A46**	*solute carrier family 25, member 46*		**CLEC7A**	*C-type lectin domain family 7, member A*	
**FTH1**	*ferritin, heavy polypeptide 1*		**EPHX2**	*epoxide hydrolase 2, cytoplasmic*	
**WDR31**	*WD repeat domain 31*		**PCDH11X**	*protocadherin 11 X-linked*	
**KCNAB3**	*potassium voltage-gated channel, shaker-related subfamily, beta member 3*		**SMARCD1**	*SWI/SNF related, matrix associated, actin dependent regulator of chromatin, subfamily d, member 1*	
**CDH13**	*cadherin 13*		**ASB15**	*ankyrin repeat and SOCS box containing 15*	
**ISLR2**	*immunoglobulin superfamily containing leucine-rich repeat 2*	✓	**YIPF2**	*Yip1 domain family, member 2*	
**FZD3**	*frizzled family receptor 3*		**MMP15**	*matrix metallopeptidase 15 (membrane-inserted)*	
**ZBED1**	*zinc finger, BED-type containing 1*		**TRMU**	*tRNA 5-methylaminomethyl-2-thiouridylate methyltransferase*	
**CDH4**	*cadherin 4, type 1, R-cadherin (retinal)*		**OR2A4**	*olfactory receptor, family 2, subfamily A, member 4*	
**PPP2R3B**	*protein phosphatase 2, regulatory subunit B'', beta*		**LRP8**	*low density lipoprotein receptor-related protein 8, apolipoprotein e receptor*	
**FBXW5**	*F-box and WD repeat domain containing 5*		**GALNT8**	*UDP-N-acetyl-alpha-D-galactosamine:polypeptide N-acetylgalactosaminyltransferase 8 (GalNAc-T8)*	
**IFI44L**	*interferon-induced protein 44-like*		**PKHD1L1**	*polycystic kidney and hepatic disease 1 (autosomal recessive)-like 1*	
**NUDCD3**	*NudC domain containing 3*		**XRCC5**	*X-ray repair complementing defective repair in Chinese hamster cells 5 (double-strand-break rejoining)*	
**TTC22**	*tetratricopeptide repeat domain 22*		**UQCR11**	*ubiquinol-cytochrome c reductase, complex III subunit XI*	
**TNIK**	*TRAF2 and NCK interacting kinase*		**FARSB**	*phenylalanyl-tRNA synthetase, beta subunit*	
**CCND2**	*cyclin D2*		**SIGLEC14**	*sialic acid binding Ig-like lectin 14*	
**APC**	*adenomatous polyposis coli*		**PYCARD**	*PYD and CARD domain containing*	
**TMEM209**	*transmembrane protein 209*		**SHC3**	*SHC (Src homology 2 domain containing) transforming protein 3*	
**ALS2**	*amyotrophic lateral sclerosis 2 (juvenile)*		**CCAR2**	*cell cycle and apoptosis regulator 2*	
**THBS2**	*thrombospondin 2*		**ALDOA**	*aldolase A, fructose-bisphosphate*	
**HES4**	*hairy and enhancer of split 4 (Drosophila)*		**PRMT7**	*protein arginine methyltransferase 7*	
**CECR1**	*cat eye syndrome chromosome region, candidate 1*		**ATP5J2-PTCD1**	*ATP5J2-PTCD1 readthrough*	

**INO80D**	*INO80 complex subunit D*		**SMTNL2**	*smoothelin-like 2*	
**ATXN7**	*ataxin 7*		**PPP2R1A**	*protein phosphatase 2, regulatory subunit A, alpha*	
**TADA2A**	*transcriptional adaptor 2A*		**WDR13**	*WD repeat domain 13*	
**NOTCH2**	*notch 2*		**SEC23IP**	*SEC23 interacting protein*	
**CLIC5**	*chloride intracellular channel 5*		**TMEM106C**	*transmembrane protein 106C*	
**FBXL12**	*F-box and leucine-rich repeat protein 12*		**KCNH1**	*potassium voltage-gated channel, subfamily H (eag-related), member 1*	
**NPAP1**	*nuclear pore associated protein 1*		**STRADA**	*STE20-related kinase adaptor alpha*	
**KCNJ5**	*potassium inwardly-rectifying channel, subfamily J, member 5*		**CNDP2**	*CNDP dipeptidase 2 (metallopeptidase M20 family)*	
**GSR**	*glutathione reductase*		**FAM208A**	*family with sequence similarity 208, member A*	✓
**ZIK1**	*zinc finger protein interacting with K protein 1*		**LIMK2**	*LIM domain kinase 2*	
**ZNF460**	*zinc finger protein 460*	✓	**ANGPTL3**	*angiopoietin-like 3*	
**MED14**	*mediator complex subunit 14*		**CFL2**	*cofilin 2 (muscle)*	
**MYO7B**	*myosin VIIB*		**EHD3**	*EH-domain containing 3*	
**MKX**	*mohawk homeobox*		**DHX58**	*DEXH (Asp-Glu-X-His) box polypeptide 58*	
**PLEKHB2**	*pleckstrin homology domain containing, family B (evectins) member 2*		**ZBTB22**	*zinc finger and BTB domain containing 22*	
**RNF152**	*ring finger protein 152*		**TNFAIP8L3**	*tumor necrosis factor, alpha-induced protein 8-like 3*	
**ZNF2**	*zinc finger protein 2*		**AGAP9**	*ArfGAP with GTPase domain, ankyrin repeat and PH domain 9*	
**SV2B**	*synaptic vesicle glycoprotein 2B*		**ABHD15**	*abhydrolase domain containing 15*	
**ZCCHC14**	*zinc finger, CCHC domain containing 14*		**S100PBP**	*S100P binding protein*	
**HIVEP3**	*human immunodeficiency virus type I enhancer binding protein 3*		**CELF3**	*CUGBP, Elav-like family member 3*	
**ANKRD46**	*ankyrin repeat domain 46*		**ANKFY1**	*ankyrin repeat and FYVE domain containing 1*	
**ACAP2**	*ArfGAP with coiled-coil, ankyrin repeat and PH domains 2*		**LMBR1**	*limb development membrane protein 1*	
**KIAA1239**	*KIAA1239*		**AGTPBP1**	*ATP/GTP binding protein 1*	

**MCM5**	*minichromosome maintenance complex component 5*		**DNAJC15**	*DnaJ (Hsp40) homolog, subfamily C, member 15*	
**CA12**	*carbonic anhydrase XII*		**BAZ2B**	*bromodomain adjacent to zinc finger domain, 2B*	✓
**IL22RA2**	*interleukin 22 receptor, alpha 2*				

Table S2. Predicted and validated mRNAs targeted by miR-584-5p. A total of 2668 transcripts were found to potentially be targeted by miR-584-5p, with a total of 3296 sites, between them 133 were experimentally validated (indicated in table by a check mark). Data were obtained by a combined bioinformatical approach using TargetScan Human v8.0 to identify mRNAs predicted to be bounded by miR-584-5p and miRTarBase release 9 to identify validated miRNA-mRNA interactions. Both databases were accessed on December 6th.

Target gene	Gene name	Validated	Target gene	Gene name	Validated
**RP11-169F17.1**	*Protein LOC400655*		**PPAT**	*phosphoribosyl pyrophosphate amidotransferase*	
**CTXN2**	*cortexin 2*		**GPLD1**	*glycosylphosphatidylinositol specific phospholipase D1*	
**MIS12**	*MIS12 kinetochore complex component*		**ZNF16**	*zinc finger protein 16*	
**HBD**	*hemoglobin, delta*		**RIMS1**	*regulating synaptic membrane exocytosis 1*	
**GLRA3**	*glycine receptor, alpha 3*		**OSBPL11**	*oxysterol binding protein-like 11*	
**CTD-2600O9.1**	*Homo sapiens uncharacterized LOC388282 (LOC388282), mRNA.*		**SLC5A6**	*solute carrier family 5 (sodium/multivitamin and iodide cotransporter), member 6*	
**RQCD1**	*RCD1 required for cell differentiation1 homolog (S. pombe)*		**PURA**	*purine-rich element binding protein A*	
**GTF2A2**	*general transcription factor IIA, 2, 12kDa*		**DBR1**	*debranching RNA lariats 1*	
**SNTG1**	*syntrophin, gamma 1*		**DCUN1D1**	*DCN1, defective in cullin neddylation 1, domain containing 1*	
**KCNE2**	*potassium voltage-gated channel, Isk-related family, member 2*		**FBLN7**	*fibulin 7*	
**ATP6V1E2**	*ATPase, H+ transporting, lysosomal 31kDa, V1 subunit E2*		**PCBD2**	*pterin-4 alpha-carbinolamine dehydratase/dimerization cofactor of hepatocyte nuclear factor 1 alpha (TCF1) 2*	
**AL139147.1**	*Uncharacterized protein*		**PABPC5**	*poly(A) binding protein, cytoplasmic 5*	
**NDUFA12**	*NADH dehydrogenase (ubiquinone) 1 alpha subcomplex, 12*		**NAV2**	*neuron navigator 2*	
**CCL5**	*chemokine (C-C motif) ligand 5*		**SLC33A1**	*solute carrier family 33 (acetyl-CoA transporter), member 1*	
**C2orf43**	*chromosome 2 open reading frame 43*		**ZNF652**	*zinc finger protein 652*	

**ZNF616**	*zinc finger protein 616*		**NOL10**	*nucleolar protein 10*	
**HIGD2B**	*HIG1 hypoxia inducible domain family, member 2B*		**TMEM108**	*transmembrane protein 108*	
**DPY30**	*dpy-30 homolog (C. elegans)*		**SAMD4A**	*sterile alpha motif domain containing 4A*	
**CCDC152**	*coiled-coil domain containing 152*		**PLEKHH1**	*pleckstrin homology domain containing, family H (with MyTH4 domain) member 1*	
**DSG3**	*desmoglein 3*		**ZNF514**	*zinc finger protein 514*	
**ANXA1**	*annexin A1*		**SCYL2**	*SCY1-like 2 (S. cerevisiae)*	
**EBPL**	*emopamil binding protein-like*		**CDC27**	*cell division cycle 27*	
**C2orf66**	*chromosome 2 open reading frame 66*		**TMEM192**	*transmembrane protein 192*	
**AC008964.1**			**GOSR1**	*golgi SNAP receptor complex member 1*	
**NPHP1**	*nephronophthisis 1 (juvenile)*		**TAP2**	*transporter 2, ATP-binding cassette, sub-family B (MDR/TAP)*	
**ZBP1**	*Z-DNA binding protein 1*		**SHISA7**	*shisa family member 7*	
**C12orf65**	*chromosome 12 open reading frame 65*		**TGFB2**	*transforming growth factor, beta 2*	
**PAXIP1-AS2**	*PAXIP1 antisense RNA 2*		**RLIM**	*ring finger protein, LIM domain interacting*	
**C17orf62**	*chromosome 17 open reading frame 62*		**PIK3C3**	*phosphatidylinositol 3-kinase, catalytic subunit type 3*	
**UTP11L**	*UTP11-like, U3 small nucleolar ribonucleoprotein, (yeast)*		**RNF157**	*ring finger protein 157*	
**ANKRD18B**	*ankyrin repeat domain 18B*		**TMPRSS11A**	*transmembrane protease, serine 11A*	
**CENPQ**	*centromere protein Q*		**KLF13**	*Kruppel-like factor 13*	
**LYPD1**	*LY6/PLAUR domain containing 1*		**MTL5**	*metallothionein-like 5, testis-specific (tesmin)*	
**GYPE**	*glycophorin E (MNS blood group)*		**TAOK1**	*TAO kinase 1*	
**ZIK1**	*zinc finger protein interacting with K protein 1*		**RANBP6**	*RAN binding protein 6*	
**NEUROD4**	*neuronal differentiation 4*		**KCNC1**	*potassium voltage-gated channel, Shaw-related subfamily, member 1*	
**UBTD2**	*ubiquitin domain containing 2*		**ABHD2**	*abhydrolase domain containing 2*	
**CYP2J2**	*cytochrome P450, family 2, subfamily J, polypeptide 2*		**MMAA**	*methylmalonic aciduria (cobalamin deficiency) cblA type*	
**TAF13**	*TAF13 RNA polymerase II, TATA box binding protein (TBP)-associated factor, 18kDa*		**MEX3C**	*mex-3 RNA binding family member C*	

**ANGPTL5**	*angiopoietin-like 5*		**LRP6**	*low density lipoprotein receptor-related protein 6*	
**ZNF268**	*zinc finger protein 268*	✓	**ZNF510**	*zinc finger protein 510*	
**BTNL3**	*butyrophilin-like 3*		**VNN1**	*vanin 1*	
**AVPR1A**	*arginine vasopressin receptor 1A*		**SLC22A9**	*solute carrier family 22 (organic anion transporter), member 9*	
**TMEM212**	*transmembrane protein 212*		**HCN1**	*hyperpolarization activated cyclic nucleotide-gated potassium channel 1*	
**C2orf83**	*chromosome 2 open reading frame 83*		**KLF12**	*Kruppel-like factor 12*	
**MORC3**	*MORC family CW-type zinc finger 3*	✓	**UBE4B**	*ubiquitination factor E4B*	✓
**FGF10**	*fibroblast growth factor 10*		**HSPA4L**	*heat shock 70kDa protein 4-like*	
**HOXA11**	*homeobox A11*		**NKAPL**	*NFKB activating protein-like*	
**SLC25A40**	*solute carrier family 25, member 40*		**SKP1**	*S-phase kinase-associated protein 1*	
**ZNF195**	*zinc finger protein 195*	✓	**TMEM170A**	*transmembrane protein 170A*	✓
**ZNF233**	*zinc finger protein 233*		**PRRG4**	*proline rich Gla (G-carboxyglutamic acid) 4 (transmembrane)*	
**TMEM248**	*transmembrane protein 248*	✓	**ZNF217**	*zinc finger protein 217*	
**AKNAD1**	*AKNA domain containing 1*		**IGDCC3**	*immunoglobulin superfamily, DCC subclass, member 3*	
**CD200**	*CD200 molecule*		**CYP4A11**	*cytochrome P450, family 4, subfamily A, polypeptide 11*	
**PRRX1**	*paired related homeobox 1*		**RAB3IP**	*RAB3A interacting protein*	✓
**AP003068.23**	*Uncharacterized protein*		**SOAT1**	*sterol O-acyltransferase 1*	
**YKT6**	*YKT6 v-SNARE homolog (S. cerevisiae)*	✓	**RGMB**	*RGM domain family, member B*	
**RP11-552I14.1**	*Uncharacterized protein*		**ZNF573**	*zinc finger protein 573*	
**HDAC1**	*histone deacetylase 1*		**TGFBR1**	*transforming growth factor, beta receptor 1*	
**CHORDC1**	*cysteine and histidine-rich domain (CHORD) containing 1*		**RHOH**	*ras homolog family member H*	
**SUCNR1**	*succinate receptor 1*		**PSMF1**	*proteasome (prosome, macropain) inhibitor subunit 1 (PI31)*	
**SLC35D3**	*solute carrier family 35, member D3*		**CNBP**	*CCHC-type zinc finger, nucleic acid binding protein*	
**E2F5**	*E2F transcription factor 5, p130-binding*	✓	**SSR1**	*signal sequence receptor, alpha*	

**FAM78B**	*family with sequence similarity 78, member B*		**ETNK1**	*ethanolamine kinase 1*	
**ADAMDEC1**	*ADAM-like, decysin 1*		**UHRF1BP1**	*UHRF1 binding protein 1*	
**C22orf24**	*chromosome 22 open reading frame 24*		**RNF125**	*ring finger protein 125, E3 ubiquitin protein ligase*	
**ANKRD18A**	*ankyrin repeat domain 18A*		**CYP4A22**	*cytochrome P450, family 4, subfamily A, polypeptide 22*	
**XCL1**	*chemokine (C motif) ligand 1*		**KLHL32**	*kelch-like family member 32*	
**SEC24D**	*SEC24 family, member D (S. cerevisiae)*		**NAA38**	*N(alpha)-acetyltransferase 38, NatC auxiliary subunit*	
**CCNC**	*cyclin C*		**ACAP2**	*ArfGAP with coiled-coil, ankyrin repeat and PH domains 2*	
**ADNP2**	*ADNP homeobox 2*		**ZFX**	*zinc finger protein, X-linked*	
**FBLN5**	*fibulin 5*		**ADAM22**	*ADAM metallopeptidase domain 22*	
**TAZ**	*tafazzin*		**ZDHHC2**	*zinc finger, DHHC-type containing 2*	
**GNG12**	*guanine nucleotide binding protein (G protein), gamma 12*	✓	**KDSR**	*3-ketodihydrosphingosine reductase*	
**PRPF19**	*pre-mRNA processing factor 19*		**PPP6C**	*protein phosphatase 6, catalytic subunit*	
**NEK3**	*NIMA-related kinase 3*		**ZNF658**	*zinc finger protein 658*	
**MCPH1**	*microcephalin 1*		**SP3**	*Sp3 transcription factor*	
**NRXN1**	*neurexin 1*		**C6orf89**	*chromosome 6 open reading frame 89*	
**POGLUT1**	*protein O-glucosyltransferase 1*		**SERPINB8**	*serpin peptidase inhibitor, clade B (ovalbumin), member 8*	
**SPIN1**	*spindlin 1*		**GRIA1**	*glutamate receptor, ionotropic, AMPA 1*	
**NDFIP2**	*Nedd4 family interacting protein 2*		**NAIP**	*NLR family, apoptosis inhibitory protein*	
**GBP5**	*guanylate binding protein 5*		**TMIE**	*transmembrane inner ear*	
**KIAA1841**	*KIAA1841*		**KCNQ3**	*potassium voltage-gated channel, KQT-like subfamily, member 3*	
**AIDA**	*axin interactor, dorsalization associated*		**SPIN3**	*spindlin family, member 3*	
**ZNF432**	*zinc finger protein 432*		**CNTNAP5**	*contactin associated protein-like 5*	
**C9orf170**	*chromosome 9 open reading frame 170*		**MICAL2**	*microtubule associated monooxygenase, calponin and LIM domain containing 2*	
**ANKLE1**	*ankyrin repeat and LEM domain containing 1*		**TTC5**	*tetratricopeptide repeat domain 5*	

**PI4K2B**	*phosphatidylinositol 4-kinase type 2 beta*		**C11orf54**	*chromosome 11 open reading frame 54*	
**CHN1**	*chimerin 1*		**EML1**	*echinoderm microtubule associated protein like 1*	
**INO80C**	*INO80 complex subunit C*		**ZNF614**	*zinc finger protein 614*	
**DYNAP**	*dynactin associated protein*		**PRKAA2**	*protein kinase, AMP-activated, alpha 2 catalytic subunit*	
**LRP11**	*low density lipoprotein receptor-related protein 11*		**SUSD1**	*sushi domain containing 1*	
**PAGE3**	*P antigen family, member 3 (prostate associated)*		**ZNF106**	*zinc finger protein 106*	
**ARL13B**	*ADP-ribosylation factor-like 13B*		**HN1**	*hematological and neurological expressed 1*	
**BCKDHB**	*branched chain keto acid dehydrogenase E1, beta polypeptide*		**SPRY4**	*sprouty homolog 4 (Drosophila)*	
**RBFOX1**	*RNA binding protein, fox-1 homolog (C. elegans) 1*		**WASF3**	*WAS protein family, member 3*	
**HDAC8**	*histone deacetylase 8*		**GPR158**	*G protein-coupled receptor 158*	
**NSRP1**	*nuclear speckle splicing regulatory protein 1*		**SGPP2**	*sphingosine-1-phosphate phosphatase 2*	
**RAP2A**	*RAP2A, member of RAS oncogene family*		**VWC2L**	*von Willebrand factor C domain containing protein 2-like*	
**CTD-2140B24.4**	*Zinc finger protein 268*		**CHMP2B**	*charged multivesicular body protein 2B*	
**CACYBP**	*calcyclin binding protein*		**CLIC6**	*chloride intracellular channel 6*	
**SIGLEC10**	*sialic acid binding Ig-like lectin 10*		**CTBP2**	*C-terminal binding protein 2*	
**PI15**	*peptidase inhibitor 15*		**ZIC1**	*Zic family member 1*	
**TRIM52**	*tripartite motif containing 52*		**NUDT16**	*nudix (nucleoside diphosphate linked moiety X)-type motif 16*	
**ZNF750**	*zinc finger protein 750*		**CREB1**	*cAMP responsive element binding protein 1*	
**RPL41**	*ribosomal protein L41*	✓	**IREB2**	*iron-responsive element binding protein 2*	
**ZMYM6NB**	*ZMYM6 neighbor*		**FAM169B**	*family with sequence similarity 169, member B*	
**THBS4**	*thrombospondin 4*		**ZMYM4**	*zinc finger, MYM-type 4*	
**SRPX**	*sushi-repeat containing protein, X-linked*		**UBXN2B**	*UBX domain protein 2B*	
**CALB1**	*calbindin 1, 28kDa*		**ATXN7L1**	*ataxin 7-like 1*	
**CD3G**	*CD3g molecule, gamma (CD3-TCR complex)*		**VSTM2A**	*V-set and transmembrane domain containing 2A*	

**SLAMF7**	*SLAM family member 7*		**TMEM178B**	*transmembrane protein 178B*	
**KRTAP19-7**	*keratin associated protein 19-7*		**GSTO2**	*glutathione S-transferase omega 2*	
**ZNF83**	*zinc finger protein 83*	✓	**MAGI3**	*membrane associated guanylate kinase, WW and PDZ domain containing 3*	
**GNPDA2**	*glucosamine-6-phosphate deaminase 2*		**PTPLAD2**	*protein tyrosine phosphatase-like A domain containing 2*	
**PPIL1**	*peptidylprolyl isomerase (cyclophilin)-like 1*	✓	**ZNF250**	*zinc finger protein 250*	
**C10orf126**	*chromosome 10 open reading frame 126*		**GID8**	*GID complex subunit 8*	
**DPPA4**	*developmental pluripotency associated 4*		**PHACTR2**	*phosphatase and actin regulator 2*	
**RPL3L**	*ribosomal protein L3-like*		**FLRT3**	*fibronectin leucine rich transmembrane protein 3*	
**C18orf63**	*chromosome 18 open reading frame 63*		**TLR7**	*toll-like receptor 7*	
**TRIQK**	*triple QxxK/R motif containing*		**MYOZ3**	*myozenin 3*	
**NUDCD2**	*NudC domain containing 2*		**SCAP**	*SREBF chaperone*	
**C12orf5**	*chromosome 12 open reading frame 5*		**ATP7B**	*ATPase, Cu++ transporting, beta polypeptide*	
**PPM1A**	*protein phosphatase, Mg2+/Mn2+ dependent, 1A*		**ARAP2**	*ArfGAP with RhoGAP domain, ankyrin repeat and PH domain 2*	
**COL1A2**	*collagen, type I, alpha 2*		**GTF3C2**	*general transcription factor IIIC, polypeptide 2, beta 110kDa*	
**ZNF583**	*zinc finger protein 583*		**LRPPRC**	*leucine-rich pentatricopeptide repeat containing*	
**ZNF512**	*zinc finger protein 512*		**FGF1**	*fibroblast growth factor 1 (acidic)*	
**EEF2**	*eukaryotic translation elongation factor 2*		**BNC2**	*basonuclin 2*	
**C5orf24**	*chromosome 5 open reading frame 24*		**UBE3C**	*ubiquitin protein ligase E3C*	
**MAP2**	*microtubule-associated protein 2*		**SH3BP4**	*SH3-domain binding protein 4*	
**SRP72**	*signal recognition particle 72kDa*		**TNFSF15**	*tumor necrosis factor (ligand) superfamily, member 15*	
**TTC13**	*tetratricopeptide repeat domain 13*		**TRIM24**	*tripartite motif containing 24*	
**WWP1**	*WW domain containing E3 ubiquitin protein ligase 1*		**CACNB2**	*calcium channel, voltage-dependent, beta 2 subunit*	
**SGTB**	*small glutamine-rich tetratricopeptide repeat (TPR)-containing, beta*		**TMX1**	*thioredoxin-related transmembrane protein 1*	
**MPC2**	*mitochondrial pyruvate carrier 2*		**SLC2A4**	*solute carrier family 2 (facilitated glucose transporter), member 4*	

**PTTG1IP**	*pituitary tumor-transforming 1 interacting protein*		**ACSL4**	*acyl-CoA synthetase long-chain family member 4*	
**PDZD11**	*PDZ domain containing 11*		**ZNF792**	*zinc finger protein 792*	
**WWTR1**	*WW domain containing transcription regulator 1*		**DSC3**	*desmocollin 3*	
**CALCR**	*calcitonin receptor*		**ARSK**	*arylsulfatase family, member K*	
**IMPA1**	*inositol(myo)-1(or 4)-monophosphatase 1*		**SRGAP1**	*SLIT-ROBO Rho GTPase activating protein 1*	
**CCDC50**	*coiled-coil domain containing 50*		**C5orf42**	*chromosome 5 open reading frame 42*	
**CDH8**	*cadherin 8, type 2*		**CDK6**	*cyclin-dependent kinase 6*	
**LRRIQ3**	*leucine-rich repeats and IQ motif containing 3*		**PDE7A**	*phosphodiesterase 7A*	
**TMEM17**	*transmembrane protein 17*		**POLR2M**	*polymerase (RNA) II (DNA directed) polypeptide M*	
**H2AFZ**	*H2A histone family, member Z*		**SCN1A**	*sodium channel, voltage-gated, type I, alpha subunit*	
**ANKRD34B**	*ankyrin repeat domain 34B*		**DST**	*dystonin*	
**ZNF791**	*zinc finger protein 791*		**DCX**	*doublecortin*	
**C14orf178**	*chromosome 14 open reading frame 178*		**QSER1**	*glutamine and serine rich 1*	
**SSH2**	*slingshot protein phosphatase 2*		**ST13**	*suppression of tumorigenicity 13 (colon carcinoma) (Hsp70 interacting protein)*	
**HINFP**	*histone H4 transcription factor*		**PREPL**	*prolyl endopeptidase-like*	
**MAGEB2**	*melanoma antigen family B, 2*		**GPR161**	*G protein-coupled receptor 161*	
**SAR1B**	*SAR1 homolog B (S. cerevisiae)*		**PNO1**	*partner of NOB1 homolog (S. cerevisiae)*	
**METTL21A**	*methyltransferase like 21A*		**EVX1**	*even-skipped homeobox 1*	
**NRG4**	*neuregulin 4*		**ZNF626**	*zinc finger protein 626*	
**PELI2**	*pellino E3 ubiquitin protein ligase family member 2*		**SLIT3**	*slit homolog 3 (Drosophila)*	
**OR52A1**	*olfactory receptor, family 52, subfamily A, member 1*		**GUCY1A3**	*guanylate cyclase 1, soluble, alpha 3*	
**TRMT11**	*tRNA methyltransferase 11 homolog (S. cerevisiae)*		**RGS7BP**	*regulator of G-protein signaling 7 binding protein*	
**SULT1C4**	*sulfotransferase family, cytosolic, 1C, member 4*		**SYPL1**	*synaptophysin-like 1*	
**THEMIS2**	*thymocyte selection associated family member 2*		**CD226**	*CD226 molecule*	
**MURC**	*muscle-related coiled-coil protein*		**SAMD8**	*sterile alpha motif domain containing 8*	

**CCNDBP1**	*cyclin D-type binding-protein 1*		**SBNO1**	*strawberry notch homolog 1 (Drosophila)*	
**GADD45A**	*growth arrest and DNA-damage-inducible, alpha*		**ARHGAP31**	*Rho GTPase activating protein 31*	
**BMPR1A**	*bone morphogenetic protein receptor, type IA*		**SPEF2**	*sperm flagellar 2*	
**CLEC5A**	*C-type lectin domain family 5, member A*		**GJD3**	*gap junction protein, delta 3, 31.9kDa*	
**MT-ATP6**	*mitochondrially encoded ATP synthase 6*		**FAM19A5**	*family with sequence similarity 19 (chemokine (C-C motif)-like), member A5*	
**SCAMP5**	*secretory carrier membrane protein 5*		**PANK3**	*pantothenate kinase 3*	
**MUC15**	*mucin 15, cell surface associated*		**TTC26**	*tetratricopeptide repeat domain 26*	
**ZNF525**	*zinc finger protein 525*		**ZNF35**	*zinc finger protein 35*	
**SLC13A3**	*solute carrier family 13 (sodium-dependent dicarboxylate transporter), member 3*		**TPK1**	*thiamin pyrophosphokinase 1*	
**C6orf10**	*chromosome 6 open reading frame 10*		**CCDC71L**	*coiled-coil domain containing 71-like*	
**RPE**	*ribulose-5-phosphate-3-epimerase*		**SRPK1**	*SRSF protein kinase 1*	
**ZNF813**	*zinc finger protein 813*		**GPR153**	*G protein-coupled receptor 153*	
**RNF11**	*ring finger protein 11*	✓	**BTN2A2**	*butyrophilin, subfamily 2, member A2*	
**MBNL3**	*muscleblind-like splicing regulator 3*		**PPP4R2**	*protein phosphatase 4, regulatory subunit 2*	
**KCND2**	*potassium voltage-gated channel, Shal-related subfamily, member 2*		**LSM11**	*LSM11, U7 small nuclear RNA associated*	
**AKAP10**	*A kinase (PRKA) anchor protein 10*		**MSANTD4**	*Myb/SANT-like DNA-binding domain containing 4 with coiled-coils*	
**RXFP1**	*relaxin/insulin-like family peptide receptor 1*		**GCH1**	*GTP cyclohydrolase 1*	
**CDC123**	*cell division cycle 123*		**EDEM3**	*ER degradation enhancer, mannosidase alpha-like 3*	
**SRSF5**	*serine/arginine-rich splicing factor 5*		**ZNF585B**	*zinc finger protein 585B*	
**FKTN**	*fukutin*		**SNX17**	*sorting nexin 17*	
**MTERF**	*mitochondrial transcription termination factor*		**UBIAD1**	*UbiA prenyltransferase domain containing 1*	
**TTC19**	*tetratricopeptide repeat domain 19*		**PPP1CB**	*protein phosphatase 1, catalytic subunit, beta isozyme*	
**MAGEB3**	*melanoma antigen family B, 3*		**CUL2**	*cullin 2*	
**ZNF24**	*zinc finger protein 24*		**FRMPD4**	*FERM and PDZ domain containing 4*	

**ZNF780B**	*zinc finger protein 780B*		**FREM2**	*FRAS1 related extracellular matrix protein 2*	
**MTX3**	*metaxin 3*		**CXCL3**	*chemokine (C-X-C motif) ligand 3*	
**SQLE**	*squalene epoxidase*		**HBS1L**	*HBS1-like (S. cerevisiae)*	
**TXNL1**	*thioredoxin-like 1*		**ZBTB26**	*zinc finger and BTB domain containing 26*	
**ZNF154**	*zinc finger protein 154*		**IGFBP5**	*insulin-like growth factor binding protein 5*	✓
**TMEM183A**	*transmembrane protein 183A*		**LILRB4**	*leukocyte immunoglobulin-like receptor, subfamily B (with TM and ITIM domains), member 4*	
**MOB1B**	*MOB kinase activator 1B*		**PCYT1B**	*phosphate cytidylyltransferase 1, choline, beta*	
**UBE2W**	*ubiquitin-conjugating enzyme E2W (putative)*		**ZNF594**	*zinc finger protein 594*	
**FGF20**	*fibroblast growth factor 20*		**C5orf51**	*chromosome 5 open reading frame 51*	
**PHF6**	*PHD finger protein 6*		**CUX2**	*cut-like homeobox 2*	
**CSGALNACT1**	*chondroitin sulfate N-acetylgalactosaminyltransferase 1*		**DNAJB14**	*DnaJ (Hsp40) homolog, subfamily B, member 14*	
**CEP19**	*centrosomal protein 19kDa*		**ROR1**	*receptor tyrosine kinase-like orphan receptor 1*	
**NCOR1**	*nuclear receptor corepressor 1*		**SLC30A1**	*solute carrier family 30 (zinc transporter), member 1*	
**ZDHHC13**	*zinc finger, DHHC-type containing 13*		**SLC5A3**	*sodium/myo-inositol cotransporter*	
**CDH13**	*cadherin 13*		**PLEKHA2**	*pleckstrin homology domain containing, family A (phosphoinositide binding specific) member 2*	
**ZNF669**	*zinc finger protein 669*		**TACR3**	*tachykinin receptor 3*	
**C14orf119**	*chromosome 14 open reading frame 119*		**AL355390.1**	*Uncharacterized protein*	
**ITGAD**	*integrin, alpha D*		**SASH1**	*SAM and SH3 domain containing 1*	
**FSTL5**	*follistatin-like 5*		**ZBTB8A**	*zinc finger and BTB domain containing 8A*	
**PURB**	*purine-rich element binding protein B*		**ZBTB25**	*zinc finger and BTB domain containing 25*	
**PRKX**	*protein kinase, X-linked*		**PYGO1**	*pygopus homolog 1 (Drosophila)*	
**FBXO28**	*F-box protein 28*		**DYNC1LI2**	*dynein, cytoplasmic 1, light intermediate chain 2*	
**RP11-181C3.1**	*Uncharacterized protein*		**PDK1**	*pyruvate dehydrogenase kinase, isozyme 1*	
**VPS41**	*vacuolar protein sorting 41 homolog (S. cerevisiae)*		**ABAT**	*4-aminobutyrate aminotransferase*	

**ACTR6**	*ARP6 actin-related protein 6 homolog (yeast)*		**ATP2B1**	*ATPase, Ca++ transporting, plasma membrane 1*	
**MZT1**	*mitotic spindle organizing protein 1*		**ANKRD44**	*ankyrin repeat domain 44*	
**GSTM2**	*glutathione S-transferase mu 2 (muscle)*		**VPS13B**	*vacuolar protein sorting 13 homolog B (yeast)*	
**CCL18**	*chemokine (C-C motif) ligand 18 (pulmonary and activation-regulated)*		**FAM110B**	*family with sequence similarity 110, member B*	
**KRTDAP**	*keratinocyte differentiation-associated protein*		**ERC1**	*ELKS/RAB6-interacting/CAST family member 1*	
**IL33**	*interleukin 33*		**C1RL**	*complement component 1, r subcomponent-like*	
**PEX5L**	*peroxisomal biogenesis factor 5-like*		**HOXD11**	*homeobox D11*	
**PIGW**	*phosphatidylinositol glycan anchor biosynthesis, class W*	✓	**TMOD2**	*tropomodulin 2 (neuronal)*	
**SPOPL**	*speckle-type POZ protein-like*		**TMEM194A**	*transmembrane protein 194A*	
**CADPS**	*Ca++-dependent secretion activator*		**CYSLTR1**	*cysteinyl leukotriene receptor 1*	
**RP11-17M16.1**	*Homo sapiens FLJ44313 protein (FLJ44313), mRNA.*		**ZNF708**	*zinc finger protein 708*	
**NET1**	*neuroepithelial cell transforming 1*		**PDE6A**	*phosphodiesterase 6A, cGMP-specific, rod, alpha*	
**COX7B**	*cytochrome c oxidase subunit VIIb*		**LOX**	*lysyl oxidase*	
**EIF2AK1**	*eukaryotic translation initiation factor 2-alpha kinase 1*		**USP37**	*ubiquitin specific peptidase 37*	
**PIAS2**	*protein inhibitor of activated STAT, 2*		**MAP9**	*microtubule-associated protein 9*	
**WIPI1**	*WD repeat domain, phosphoinositide interacting 1*		**VN1R1**	*vomeronasal 1 receptor 1*	
**SLITRK6**	*SLIT and NTRK-like family, member 6*		**YTHDF3**	*YTH domain family, member 3*	
**COL25A1**	*collagen, type XXV, alpha 1*		**C17orf75**	*chromosome 17 open reading frame 75*	
**CYP1A2**	*cytochrome P450, family 1, subfamily A, polypeptide 2*		**AKNA**	*AT-hook transcription factor*	
**ABCB10**	*ATP-binding cassette, sub-family B (MDR/TAP), member 10*		**SPRY1**	*sprouty homolog 1, antagonist of FGF signaling (Drosophila)*	
**ATG3**	*autophagy related 3*		**CSMD3**	*CUB and Sushi multiple domains 3*	
**KRT40**	*keratin 40*		**HIF3A**	*hypoxia inducible factor 3, alpha subunit*	
**PIGP**	*phosphatidylinositol glycan anchor biosynthesis, class P*		**GLTP**	*glycolipid transfer protein*	
**GATAD1**	*GATA zinc finger domain containing 1*		**GPN2**	*GPN-loop GTPase 2*	

**TIMM17A**	*translocase of inner mitochondrial membrane 17 homolog A (yeast)*		**ZNF182**	*zinc finger protein 182*	
**HDGFRP3**	*Hepatoma-derived growth factor-related protein 3*		**PIK3C2B**	*phosphatidylinositol-4-phosphate 3-kinase, catalytic subunit type 2 beta*	
**SGCD**	*sarcoglycan, delta (35kDa dystrophin-associated glycoprotein)*		**SLC4A5**	*solute carrier family 4 (sodium bicarbonate cotransporter), member 5*	
**PM20D1**	*peptidase M20 domain containing 1*		**GOPC**	*golgi-associated PDZ and coiled-coil motif containing*	
**RP11-180C1.1**	*Uncharacterized protein*		**GPATCH8**	*G patch domain containing 8*	
**PPP1R12A**	*protein phosphatase 1, regulatory subunit 12A*		**ACVR1C**	*activin A receptor, type IC*	
**C2orf27A**	*chromosome 2 open reading frame 27A*		**SUPT16H**	*suppressor of Ty 16 homolog (S. cerevisiae)*	
**PCMT1**	*protein-L-isoaspartate (D-aspartate) O-methyltransferase*		**ZNF816**	*zinc finger protein 816*	
**DCTN4**	*dynactin 4 (p62)*	✓	**RAD23B**	*RAD23 homolog B (S. cerevisiae)*	
**S100A14**	*S100 calcium binding protein A14*		**RCAN3**	*RCAN family member 3*	
**C2orf27B**	*chromosome 2 open reading frame 27B*		**ALKBH8**	*alkB, alkylation repair homolog 8 (E. coli)*	
**UBQLN3**	*ubiquilin 3*		**PIWIL1**	*piwi-like RNA-mediated gene silencing 1*	
**FKBP9**	*FK506 binding protein 9, 63 kDa*		**TMEM167B**	*transmembrane protein 167B*	
**XPO7**	*exportin 7*		**EDA**	*ectodysplasin A*	
**STAMBP**	*STAM binding protein*	✓	**PCSK6**	*proprotein convertase subtilisin/kexin type 6*	
**C12orf23**	*chromosome 12 open reading frame 23*		**C21orf91**	*chromosome 21 open reading frame 91*	
**KCNJ6**	*potassium inwardly-rectifying channel, subfamily J, member 6*	✓	**GMPS**	*guanine monphosphate synthase*	
**KPNB1**	*karyopherin (importin) beta 1*		**FOXO3**	*forkhead box O3*	
**MUC16**	*mucin 16, cell surface associated*		**KCNH5**	*potassium voltage-gated channel, subfamily H (eag-related), member 5*	
**RAX**	*retina and anterior neural fold homeobox*		**SEC22C**	*SEC22 vesicle trafficking protein homolog C (S. cerevisiae)*	
**TRUB1**	*TruB pseudouridine (psi) synthase family member 1*		**MPP6**	*membrane protein, palmitoylated 6 (MAGUK p55 subfamily member 6)*	
**BTLA**	*B and T lymphocyte associated*		**HMGB1**	*high mobility group box 1*	
**ITGBL1**	*integrin, beta-like 1 (with EGF-like repeat domains)*		**NKD1**	*naked cuticle homolog 1 (Drosophila)*	
**GTF2A1**	*general transcription factor IIA, 1, 19/37kDa*		**DUOX2**	*dual oxidase 2*	

**NEGR1**	*neuronal growth regulator 1*		**TET3**	*tet methylcytosine dioxygenase 3*	✓
**SETD1B**	*SET domain containing 1B*		**SLC38A1**	*solute carrier family 38, member 1*	
**PUS7L**	*pseudouridylate synthase 7 homolog (S. cerevisiae)-like*		**EYA1**	*eyes absent homolog 1 (Drosophila)*	
**M1AP**	*meiosis 1 associated protein*		**MFAP5**	*microfibrillar associated protein 5*	
**PTP4A1**	*protein tyrosine phosphatase type IVA, member 1*	✓	**TMEM184C**	*transmembrane protein 184C*	
**ZNF354C**	*zinc finger protein 354C*		**NEDD1**	*neural precursor cell expressed, developmentally down-regulated 1*	
**CMTM1**	*CKLF-like MARVEL transmembrane domain containing 1*		**SLC16A7**	*solute carrier family 16 (monocarboxylate transporter), member 7*	
**TCF12**	*transcription factor 12*		**ABL2**	*c-abl oncogene 2, non-receptor tyrosine kinase*	
**COX17**	*COX17 cytochrome c oxidase copper chaperone*		**MFSD6**	*major facilitator superfamily domain containing 6*	
**IFNA6**	*interferon, alpha 6*		**SNTB1**	*syntrophin, beta 1 (dystrophin-associated protein A1, 59kDa, basic component 1)*	
**CKLF-CMTM1**	*CKLF-CMTM1 readthrough*		**SDC3**	*syndecan 3*	
**BMP3**	*bone morphogenetic protein 3*		**OXTR**	*oxytocin receptor*	
**SLC28A3**	*solute carrier family 28 (concentrative nucleoside transporter), member 3*		**PPP1R12B**	*protein phosphatase 1, regulatory subunit 12B*	
**C5orf47**	*chromosome 5 open reading frame 47*		**RBMS3**	*RNA binding motif, single stranded interacting protein 3*	
**TCEANC**	*transcription elongation factor A (SII) N-terminal and central domain containing*		**KIAA1549L**	*KIAA1549-like*	
**AK7**	*adenylate kinase 7*		**ESR1**	*estrogen receptor 1*	
**CPLX3**	*complexin 3*		**BMP6**	*bone morphogenetic protein 6*	
**PRKCQ**	*protein kinase C, theta*		**ZNF738**	*zinc finger protein 738*	
**DAND5**	*DAN domain family member 5, BMP antagonist*		**MCM4**	*minichromosome maintenance complex component 4*	
**PPFIA2**	*protein tyrosine phosphatase, receptor type, f polypeptide (PTPRF), interacting protein (liprin), alpha 2*		**ASXL2**	*additional sex combs like 2 (Drosophila)*	
**F11**	*coagulation factor XI*		**P2RY1**	*purinergic receptor P2Y, G-protein coupled, 1*	
**XPR1**	*xenotropic and polytropic retrovirus receptor 1*		**THBD**	*thrombomodulin*	
**ETF1**	*eukaryotic translation termination factor 1*		**FITM2**	*fat storage-inducing transmembrane protein 2*	
**HGD**	*homogentisate 1,2-dioxygenase*		**DZIP3**	*DAZ interacting zinc finger protein 3*	

**PLN**	*phospholamban*		**TTLL7**	*tubulin tyrosine ligase-like family, member 7*	
**TRIM44**	*tripartite motif containing 44*		**RUNX1**	*runt-related transcription factor 1*	
**CTD-2054N24.2**	*Uncharacterized protein*		**TRAF3IP1**	*TNF receptor-associated factor 3 interacting protein 1*	
**LOXHD1**	*lipoxygenase homology domains 1*		**ESRRG**	*estrogen-related receptor gamma*	
**KIF2A**	*kinesin heavy chain member 2A*		**FAM167A**	*family with sequence similarity 167, member A*	
**BHLHB9**	*basic helix-loop-helix domain containing, class B, 9*		**FUT9**	*fucosyltransferase 9 (alpha (1,3) fucosyltransferase)*	
**QRFPR**	*pyroglutamylated RFamide peptide receptor*		**LAMTOR3**	*late endosomal/lysosomal adaptor, MAPK and MTOR activator 3*	
**RUNX2**	*runt-related transcription factor 2*		**KIF11**	*kinesin family member 11*	
**MEIS2**	*Meis homeobox 2*		**KDELR2**	*KDEL (Lys-Asp-Glu-Leu) endoplasmic reticulum protein retention receptor 2*	
**MYRIP**	*myosin VIIA and Rab interacting protein*		**C6**	*complement component 6*	
**ABHD17C**	*abhydrolase domain containing 17C*		**KITLG**	*KIT ligand*	
**SMIM20**	*small integral membrane protein 20*		**COX7A2L**	*cytochrome c oxidase subunit VIIa polypeptide 2 like*	
**ZNF585A**	*zinc finger protein 585A*		**FASTK**	*Fas-activated serine/threonine kinase*	
**ITGAE**	*integrin, alpha E (antigen CD103, human mucosal lymphocyte antigen 1; alpha polypeptide)*		**TMC8**	*transmembrane channel-like 8*	
**C4orf45**	*chromosome 4 open reading frame 45*		**CPEB3**	*cytoplasmic polyadenylation element binding protein 3*	
**LACTB2**	*lactamase, beta 2*		**PDE12**	*phosphodiesterase 12*	
**SLC25A23**	*solute carrier family 25 (mitochondrial carrier; phosphate carrier), member 23*		**LANCL3**	*LanC lantibiotic synthetase component C-like 3 (bacterial)*	
**ZNF415**	*zinc finger protein 415*	✓	**BRWD3**	*bromodomain and WD repeat domain containing 3*	
**SPOCK3**	*sparc/osteonectin, cwcv and kazal-like domains proteoglycan (testican) 3*		**CLPB**	*ClpB caseinolytic peptidase B homolog (E. coli)*	
**KLRK1**	*killer cell lectin-like receptor subfamily K, member 1*		**GLIPR1**	*GLI pathogenesis-related 1*	
**MEX3B**	*mex-3 RNA binding family member B*		**ZBTB41**	*zinc finger and BTB domain containing 41*	
**PATE4**	*prostate and testis expressed 4*		**ACADSB**	*acyl-CoA dehydrogenase, short/branched chain*	
**HOOK1**	*hook microtubule-tethering protein 1*	✓	**NUTF2**	*nuclear transport factor 2*	
**DEF6**	*differentially expressed in FDCP 6 homolog (mouse)*		**ALPK1**	*alpha-kinase 1*	

**ZKSCAN8**	*zinc finger with KRAB and SCAN domains 8*		**PLSCR1**	*phospholipid scramblase 1*	
**FRRS1L**	*ferric-chelate reductase 1-like*		**SEC24A**	*SEC24 family, member A (S. cerevisiae)*	
**RAB30**	*RAB30, member RAS oncogene family*		**ARSE**	*arylsulfatase E (chondrodysplasia punctata 1)*	
**ZDHHC15**	*zinc finger, DHHC-type containing 15*		**IGF2BP1**	*insulin-like growth factor 2 mRNA binding protein 1*	
**COQ10B**	*coenzyme Q10 homolog B (S. cerevisiae)*		**SLC25A46**	*solute carrier family 25, member 46*	
**CCL13**	*chemokine (C-C motif) ligand 13*		**TIAL1**	*TIA1 cytotoxic granule-associated RNA binding protein-like 1*	
**PLCZ1**	*phospholipase C, zeta 1*		**ZSCAN30**	*zinc finger and SCAN domain containing 30*	
**ADAM28**	*ADAM metallopeptidase domain 28*		**STMN2**	*stathmin-like 2*	
**PTHLH**	*parathyroid hormone-like hormone*		**USH2A**	*Usher syndrome 2A (autosomal recessive, mild)*	
**RAB31**	*RAB31, member RAS oncogene family*		**ABI3BP**	*ABI family, member 3 (NESH) binding protein*	
**GBP2**	*guanylate binding protein 2, interferon-inducible*		**UNC119B**	*unc-119 homolog B (C. elegans)*	
**INMT**	*indolethylamine N-methyltransferase*		**FLNB**	*filamin B, beta*	
**TBX20**	*T-box 20*		**SGPP1**	*sphingosine-1-phosphate phosphatase 1*	
**CADM2**	*cell adhesion molecule 2*		**KIAA1549**	*KIAA1549*	
**TRIM61**	*tripartite motif containing 61*		**CPM**	*carboxypeptidase M*	
**FLVCR2**	*feline leukemia virus subgroup C cellular receptor family, member 2*		**GFPT1**	*glutamine--fructose-6-phosphate transaminase 1*	
**DHRSX**	*dehydrogenase/reductase (SDR family) X-linked*		**HELZ**	*helicase with zinc finger*	
**ZNF84**	*zinc finger protein 84*		**TMEM135**	*transmembrane protein 135*	
**CSNK1A1L**	*casein kinase 1, alpha 1-like*		**CCDC77**	*coiled-coil domain containing 77*	
**KBTBD6**	*kelch repeat and BTB (POZ) domain containing 6*		**EIF2S2**	*eukaryotic translation initiation factor 2, subunit 2 beta, 38kDa*	
**CXorf61**	*chromosome X open reading frame 61*		**C7orf60**	*chromosome 7 open reading frame 60*	
**PACSIN1**	*protein kinase C and casein kinase substrate in neurons 1*		**FZD1**	*frizzled family receptor 1*	
**FOXA1**	*forkhead box A1*	✓	**C15orf38**	*chromosome 15 open reading frame 38*	
**OLA1**	*Obg-like ATPase 1*	✓	**FCF1**	*FCF1 rRNA-processing protein*	

**ZNF846**	*zinc finger protein 846*	✓	**TPGS2**	*tubulin polyglutamylase complex subunit 2*	✓
**SEPP1**	*selenoprotein P, plasma, 1*		**ZDHHC14**	*zinc finger, DHHC-type containing 14*	
**KLF6**	*Kruppel-like factor 6*		**ZMPSTE24**	*zinc metallopeptidase STE24*	
**LYST**	*lysosomal trafficking regulator*		**GABRG1**	*gamma-aminobutyric acid (GABA) A receptor, gamma 1*	
**CDKL4**	*cyclin-dependent kinase-like 4*		**TET2**	*tet methylcytosine dioxygenase 2*	
**WWC2**	*WW and C2 domain containing 2*		**MACROD2**	*MACRO domain containing 2*	
**AVIL**	*advillin*		**LRRC19**	*leucine rich repeat containing 19*	
**LUZP2**	*leucine zipper protein 2*		**SMARCE1**	*SWI/SNF related, matrix associated, actin dependent regulator of chromatin, subfamily e, member 1*	
**SMLR1**	*small leucine-rich protein 1*		**ATAD1**	*ATPase family, AAA domain containing 1*	
**METTL14**	*methyltransferase like 14*		**ZNF45**	*zinc finger protein 45*	
**ERI1**	*exoribonuclease 1*		**CLDN1**	*claudin 1*	
**KRAS**	*Kirsten rat sarcoma viral oncogene homolog*		**PHF20**	*PHD finger protein 20*	
**SLAMF8**	*SLAM family member 8*		**KCNK5**	*potassium channel, subfamily K, member 5*	
**GRID2**	*glutamate receptor, ionotropic, delta 2*		**THAP2**	*THAP domain containing, apoptosis associated protein 2*	
**TREH**	*trehalase (brush-border membrane glycoprotein)*		**STAG2**	*stromal antigen 2*	
**SLC4A1AP**	*solute carrier family 4 (anion exchanger), member 1, adaptor protein*		**GABRB2**	*gamma-aminobutyric acid (GABA) A receptor, beta 2*	
**OSER1**	*oxidative stress responsive serine-rich 1*		**C11orf1**	*chromosome 11 open reading frame 1*	
**TM4SF18**	*transmembrane 4 L six family member 18*		**SKIL**	*SKI-like oncogene*	
**PPP2CA**	*protein phosphatase 2, catalytic subunit, alpha isozyme*	✓	**UBE2J1**	*ubiquitin-conjugating enzyme E2, J1*	
**NAA30**	*N(alpha)-acetyltransferase 30, NatC catalytic subunit*		**PAN3**	*PAN3 poly(A) specific ribonuclease subunit homolog (S. cerevisiae)*	
**ELL2**	*elongation factor, RNA polymerase II, 2*	✓	**MYSM1**	*Myb-like, SWIRM and MPN domains 1*	
**FAM9C**	*family with sequence similarity 9, member C*		**RLN2**	*relaxin 2*	
**FAM120AOS**	*family with sequence similarity 120A opposite strand*		**KIF1C**	*kinesin family member 1C*	
**FAXC**	*failed axon connections homolog (Drosophila)*		**GCOM1**	*GRINL1A complex locus 1*	

**CH25H**	*cholesterol 25-hydroxylase*		**KLHL23**	*kelch-like family member 23*	
**USP3**	*ubiquitin specific peptidase 3*		**TGFBI**	*transforming growth factor, beta-induced, 68kDa*	
**ZNF121**	*zinc finger protein 121*	✓	**TMTC2**	*transmembrane and tetratricopeptide repeat containing 2*	
**PCDHB11**	*protocadherin beta 11*		**ANKS1A**	*ankyrin repeat and sterile alpha motif domain containing 1A*	
**USP45**	*ubiquitin specific peptidase 45*		**WNT2B**	*wingless-type MMTV integration site family, member 2B*	
**RP11-867G23.8**	*Uncharacterized protein*		**DIAPH3**	*diaphanous-related formin 3*	
**METTL21B**	*methyltransferase like 21B*		**PSEN1**	*presenilin 1*	
**CHST15**	*carbohydrate (N-acetylgalactosamine 4-sulfate 6-O) sulfotransferase 15*		**BTN2A1**	*butyrophilin, subfamily 2, member A1*	
**RTN1**	*reticulon 1*		**MOSPD2**	*motile sperm domain containing 2*	
**ZNF92**	*zinc finger protein 92*		**KCNA7**	*potassium voltage-gated channel, shaker-related subfamily, member 7*	
**FBXW7**	*F-box and WD repeat domain containing 7, E3 ubiquitin protein ligase*		**CDH6**	*cadherin 6, type 2, K-cadherin (fetal kidney)*	
**PTBP2**	*polypyrimidine tract binding protein 2*		**CFL2**	*cofilin 2 (muscle)*	
**BRI3BP**	*BRI3 binding protein*		**NCAM2**	*neural cell adhesion molecule 2*	
**MAP7D2**	*MAP7 domain containing 2*		**SFR1**	*SWI5-dependent recombination repair 1*	
**FAM211A**	*family with sequence similarity 211, member A*		**C6orf141**	*chromosome 6 open reading frame 141*	
**ARL2BP**	*ADP-ribosylation factor-like 2 binding protein*		**H2AFJ**	*H2A histone family, member J*	
**MATN3**	*matrilin 3*		**ERO1LB**	*ERO1-like beta (S. cerevisiae)*	
**KRTAP4-2**	*keratin associated protein 4-2*		**UBE3A**	*ubiquitin protein ligase E3A*	
**IKBIP**	*IKBKB interacting protein*		**ATP5S**	*ATP synthase, H+ transporting, mitochondrial Fo complex, subunit s (factor B)*	
**CCNYL1**	*cyclin Y-like 1*		**CD84**	*CD84 molecule*	
**STK24**	*serine/threonine kinase 24*		**PIAS1**	*protein inhibitor of activated STAT, 1*	
**CBX3**	*chromobox homolog 3*		**KCTD7**	*potassium channel tetramerization domain containing 7*	
**C12orf52**	*chromosome 12 open reading frame 52*		**AP5M1**	*adaptor-related protein complex 5, mu 1 subunit*	
**ANGPTL7**	*angiopoietin-like 7*		**ATG14**	*autophagy related 14*	

**POLR3B**	*polymerase (RNA) III (DNA directed) polypeptide B*		**PIP5K1C**	*phosphatidylinositol-4-phosphate 5-kinase, type I, gamma*	
**DCTN3**	*dynactin 3 (p22)*		**CDKL2**	*cyclin-dependent kinase-like 2 (CDC2-related kinase)*	
**GPC6**	*glypican 6*		**AGT**	*angiotensinogen (serpin peptidase inhibitor, clade A, member 8)*	
**C8orf48**	*chromosome 8 open reading frame 48*		**ZNF491**	*zinc finger protein 491*	
**SLC30A7**	*solute carrier family 30 (zinc transporter), member 7*		**HECW1**	*HECT, C2 and WW domain containing E3 ubiquitin protein ligase 1*	
**TMPRSS11BNL**	*TMPRSS11B N-terminal like*		**AADAT**	*aminoadipate aminotransferase*	
**AC005477.1**			**TMEM127**	*transmembrane protein 127*	
**ENTPD1**	*ectonucleoside triphosphate diphosphohydrolase 1*		**HIPK2**	*homeodomain interacting protein kinase 2*	
**MTAP**	*methylthioadenosine phosphorylase*		**KCNJ5**	*potassium inwardly-rectifying channel, subfamily J, member 5*	
**RAB8B**	*RAB8B, member RAS oncogene family*		**TAL1**	*T-cell acute lymphocytic leukemia 1*	
**ZNF674**	*zinc finger protein 674*		**ATRN**	*attractin*	
**RAB4B**	*RAB4B, member RAS oncogene family*		**GRIN2B**	*glutamate receptor, ionotropic, N-methyl D-aspartate 2B*	
**BPIFB2**	*BPI fold containing family B, member 2*		**FAM135B**	*family with sequence similarity 135, member B*	
**RINL**	*Ras and Rab interactor-like*		**SORBS1**	*sorbin and SH3 domain containing 1*	
**MCIDAS**	*multiciliate differentiation and DNA synthesis associated cell cycle protein*		**FAM83F**	*family with sequence similarity 83, member F*	
**MCFD2**	*multiple coagulation factor deficiency 2*		**PKHD1**	*polycystic kidney and hepatic disease 1 (autosomal recessive)*	
**IYD**	*iodotyrosine deiodinase*		**ZNF37A**	*zinc finger protein 37A*	
**CBLL1**	*Cbl proto-oncogene-like 1, E3 ubiquitin protein ligase*		**SCN8A**	*sodium channel, voltage gated, type VIII, alpha subunit*	
**NUFIP2**	*nuclear fragile X mental retardation protein interacting protein 2*		**TSPAN2**	*tetraspanin 2*	
**DCTN5**	*dynactin 5 (p25)*		**CCDC88A**	*coiled-coil domain containing 88A*	
**C17orf105**	*chromosome 17 open reading frame 105*		**PAX9**	*paired box 9*	
**LIPA**	*lipase A, lysosomal acid, cholesterol esterase*		**CPSF2**	*cleavage and polyadenylation specific factor 2, 100kDa*	
**DCAF16**	*DDB1 and CUL4 associated factor 16*		**FBN2**	*fibrillin 2*	
**DNAJC6**	*DnaJ (Hsp40) homolog, subfamily C, member 6*		**KMT2A**	*lysine (K)-specific methyltransferase 2A*	

**EPDR1**	*ependymin related 1*		**WDR26**	*WD repeat domain 26*	
**ATRX**	*alpha thalassemia/mental retardation syndrome X-linked*		**DTHD1**	*death domain containing 1*	
**AC069547.2**	*Uncharacterized protein*		**USP32**	*ubiquitin specific peptidase 32*	
**AC144568.2**	*Uncharacterized protein*		**TMA7**	*translation machinery associated 7 homolog (S. cerevisiae)*	
**MAMDC2**	*MAM domain containing 2*		**TLR4**	*toll-like receptor 4*	
**C14orf166**	*chromosome 14 open reading frame 166*		**DUSP28**	*dual specificity phosphatase 28*	
**HAPLN1**	*hyaluronan and proteoglycan link protein 1*		**CLN5**	*ceroid-lipofuscinosis, neuronal 5*	
**TMED2**	*transmembrane emp24 domain trafficking protein 2*		**FAM155B**	*family with sequence similarity 155, member B*	
**ZNF740**	*zinc finger protein 740*		**MGA**	*MGA, MAX dimerization protein*	
**AK3**	*adenylate kinase 3*		**TMEM139**	*transmembrane protein 139*	
**KCTD12**	*potassium channel tetramerization domain containing 12*		**GATAD2B**	*GATA zinc finger domain containing 2B*	
**DPP8**	*dipeptidyl-peptidase 8*		**PITX1**	*paired-like homeodomain 1*	
**SRR**	*serine racemase*		**PRKD3**	*protein kinase D3*	
**EMC4**	*ER membrane protein complex subunit 4*		**PPARGC1B**	*peroxisome proliferator-activated receptor gamma, coactivator 1 beta*	
**PEX10**	*peroxisomal biogenesis factor 10*		**ZNF790**	*zinc finger protein 790*	
**ZNF772**	*zinc finger protein 772*		**TLR3**	*toll-like receptor 3*	
**AICDA**	*activation-induced cytidine deaminase*		**IL17RD**	*interleukin 17 receptor D*	
**FAM198B**	*family with sequence similarity 198, member B*		**PGM3**	*phosphoglucomutase 3*	
**ETS1**	*v-ets avian erythroblastosis virus E26 oncogene homolog 1*		**PAWR**	*PRKC, apoptosis, WT1, regulator*	
**ETV5**	*ets variant 5*		**TTBK2**	*tau tubulin kinase 2*	
**MAN2A1**	*mannosidase, alpha, class 2A, member 1*		**FDX1**	*ferredoxin 1*	
**BTBD10**	*BTB (POZ) domain containing 10*		**PPIG**	*peptidylprolyl isomerase G (cyclophilin G)*	
**NR1D2**	*nuclear receptor subfamily 1, group D, member 2*		**ZBTB44**	*zinc finger and BTB domain containing 44*	
**SSX2IP**	*synovial sarcoma, X breakpoint 2 interacting protein*		**KCNK3**	*potassium channel, subfamily K, member 3*	
**UCHL5**	*ubiquitin carboxyl-terminal hydrolase L5*		**ADAM23**	*ADAM metallopeptidase domain 23*	

**TCF24**	*transcription factor 24*		**HEBP2**	*heme binding protein 2*	
**REL**	*v-rel avian reticuloendotheliosis viral oncogene homolog*		**FOXN3**	*forkhead box N3*	
**RPGRIP1**	*retinitis pigmentosa GTPase regulator interacting protein 1*		**FLT3**	*fms-related tyrosine kinase 3*	
**DGKE**	*diacylglycerol kinase, epsilon 64kDa*		**HACE1**	*HECT domain and ankyrin repeat containing E3 ubiquitin protein ligase 1*	
**C17orf58**	*chromosome 17 open reading frame 58*		**CLIC2**	*chloride intracellular channel 2*	
**RMI2**	*RecQ mediated genome instability 2*		**DLEU1**	*deleted in lymphocytic leukemia 1 (non-protein coding)*	
**RSPO1**	*R-spondin 1*		**VCL**	*vinculin*	
**IL6ST**	*interleukin 6 signal transducer (gp130, oncostatin M receptor)*		**RHOBTB1**	*Rho-related BTB domain containing 1*	
**STX7**	*syntaxin 7*		**TTC30A**	*tetratricopeptide repeat domain 30A*	
**FBXO4**	*F-box protein 4*		**TBL1X**	*transducin (beta)-like 1X-linked*	
**HIST1H2BK**	*histone cluster 1, H2bk*		**TMED5**	*transmembrane emp24 protein transport domain containing 5*	
**HSPA13**	*heat shock protein 70kDa family, member 13*		**EMR2**	*egf-like module containing, mucin-like, hormone receptor-like 2*	
**MARCH11**	*membrane-associated ring finger (C3HC4) 11*		**ALDH1L2**	*aldehyde dehydrogenase 1 family, member L2*	
**LCMT2**	*leucine carboxyl methyltransferase 2*		**ADD3**	*adducin 3 (gamma)*	
**C8orf31**	*chromosome 8 open reading frame 31*		**MPDZ**	*multiple PDZ domain protein*	
**TRABD2A**	*TraB domain containing 2A*		**TMEM254**	*transmembrane protein 254*	
**PHF17**	*PHD finger protein 17*		**MARCH8**	*membrane-associated ring finger (C3HC4) 8, E3 ubiquitin protein ligase*	
**SKIDA1**	*SKI/DACH domain containing 1*		**SLAIN2**	*SLAIN motif family, member 2*	
**FAM127C**	*family with sequence similarity 127, member C*		**ZNF780A**	*zinc finger protein 780A*	
**GRB14**	*growth factor receptor-bound protein 14*		**SLC8A1**	*solute carrier family 8 (sodium/calcium exchanger), member 1*	
**RIC3**	*RIC3 acetylcholine receptor chaperone*		**C1orf56**	*chromosome 1 open reading frame 56*	
**TMX4**	*thioredoxin-related transmembrane protein 4*		**ABHD4**	*abhydrolase domain containing 4*	
**PPT1**	*palmitoyl-protein thioesterase 1*		**CCDC80**	*coiled-coil domain containing 80*	

**ZNF280B**	*zinc finger protein 280B*		**RBMS2**	*RNA binding motif, single stranded interacting protein 2*	
**EDDM3B**	*epididymal protein 3B*		**SH3PXD2A**	*SH3 and PX domains 2A*	
**EIF3A**	*eukaryotic translation initiation factor 3, subunit A*		**ACOT9**	*acyl-CoA thioesterase 9*	
**PTCH2**	*patched 2*		**PON1**	*paraoxonase 1*	
**TTC14**	*tetratricopeptide repeat domain 14*		**FABP4**	*fatty acid binding protein 4, adipocyte*	
**WFDC13**	*WAP four-disulfide core domain 13*		**LURAP1L**	*leucine rich adaptor protein 1-like*	
**ZNF655**	*zinc finger protein 655*	✓	**STON2**	*stonin 2*	
**CRISP2**	*cysteine-rich secretory protein 2*		**CBFA2T2**	*core-binding factor, runt domain, alpha subunit 2; translocated to, 2*	
**ZNF117**	*zinc finger protein 117*	✓	**SNRPD1**	*small nuclear ribonucleoprotein D1 polypeptide 16kDa*	
**EIF3J**	*eukaryotic translation initiation factor 3, subunit J*		**LIN7A**	*lin-7 homolog A (C. elegans)*	
**LMBRD1**	*LMBR1 domain containing 1*		**EPC2**	*enhancer of polycomb homolog 2 (Drosophila)*	
**PKD2L2**	*polycystic kidney disease 2-like 2*		**SLK**	*STE20-like kinase*	
**XKR4**	*XK, Kell blood group complex subunit-related family, member 4*		**RECK**	*reversion-inducing-cysteine-rich protein with kazal motifs*	
**TC2N**	*tandem C2 domains, nuclear*		**NAA35**	*N(alpha)-acetyltransferase 35, NatC auxiliary subunit*	
**HOXA4**	*homeobox A4*		**SLC35B3**	*solute carrier family 35 (adenosine 3′-phospho 5′-phosphosulfate transporter), member B3*	
**XIAP**	*X-linked inhibitor of apoptosis*	✓	**PTPRB**	*protein tyrosine phosphatase, receptor type, B*	
**MPLKIP**	*M-phase specific PLK1 interacting protein*		**RWDD1**	*RWD domain containing 1*	
**FKBP1C**	*FK506 binding protein 1C*		**OSGEPL1**	*O-sialoglycoprotein endopeptidase-like 1*	
**CTXN3**	*cortexin 3*		**CYP1B1**	*cytochrome P450, family 1, subfamily B, polypeptide 1*	
**PLD5**	*phospholipase D family, member 5*		**ZNF33B**	*zinc finger protein 33B*	
**LL22NC03-63E9.3**	*Uncharacterized protein*		**AGBL3**	*ATP/GTP binding protein-like 3*	
**ZNF551**	*zinc finger protein 551*		**FBXL20**	*F-box and leucine-rich repeat protein 20*	
**ARMC10**	*armadillo repeat containing 10*		**BRAP**	*BRCA1 associated protein*	
**ARIH2OS**	*ariadne homolog 2 opposite strand*		**ABCB7**	*ATP-binding cassette, sub-family B (MDR/TAP), member 7*	

**CYP3A5**	*cytochrome P450, family 3, subfamily A, polypeptide 5*		**PHACTR4**	*phosphatase and actin regulator 4*	
**DISC1**	*disrupted in schizophrenia 1*		**AJAP1**	*adherens junctions associated protein 1*	
**LYPD6**	*LY6/PLAUR domain containing 6*		**KIAA0586**	*KIAA0586*	
**GSTK1**	*glutathione S-transferase kappa 1*		**GCNT1**	*glucosaminyl (N-acetyl) transferase 1, core 2*	
**PFN2**	*profilin 2*		**ZNF260**	*zinc finger protein 260*	
**GALNTL6**	*UDP-N-acetyl-alpha-D-galactosamine:polypeptide N-acetylgalactosaminyltransferase-like 6*		**TRIM71**	*tripartite motif containing 71, E3 ubiquitin protein ligase*	
**LRRC8B**	*leucine rich repeat containing 8 family, member B*		**SLC5A12**	*solute carrier family 5 (sodium/monocarboxylate cotransporter), member 12*	
**EPHA7**	*EPH receptor A7*		**SMAD2**	*SMAD family member 2*	
**IPMK**	*inositol polyphosphate multikinase*		**LRRC59**	*leucine rich repeat containing 59*	
**UHMK1**	*U2AF homology motif (UHM) kinase 1*		**ARNTL2**	*aryl hydrocarbon receptor nuclear translocator-like 2*	
**CCT5**	*chaperonin containing TCP1, subunit 5 (epsilon)*		**AGAP1**	*ArfGAP with GTPase domain, ankyrin repeat and PH domain 1*	
**DNER**	*delta/notch-like EGF repeat containing*		**CTH**	*cystathionase (cystathionine gamma-lyase)*	
**NANP**	*N-acetylneuraminic acid phosphatase*		**RUNX3**	*runt-related transcription factor 3*	
**AKAP12**	*A kinase (PRKA) anchor protein 12*		**SRRD**	*SRR1 domain containing*	
**CASR**	*calcium-sensing receptor*		**PRRC2C**	*proline-rich coiled-coil 2C*	
**AS3MT**	*arsenic (+3 oxidation state) methyltransferase*		**PAX7**	*paired box 7*	
**C18orf25**	*chromosome 18 open reading frame 25*		**CMSS1**	*cms1 ribosomal small subunit homolog (yeast)*	
**ZNF431**	*zinc finger protein 431*		**RTN4IP1**	*reticulon 4 interacting protein 1*	
**NT5E**	*5′-nucleotidase, ecto (CD73)*		**GTPBP10**	*GTP-binding protein 10 (putative)*	
**BRCC3**	*BRCA1/BRCA2-containing complex, subunit 3*		**NXPE3**	*neurexophilin and PC-esterase domain family, member 3*	
**MAGT1**	*magnesium transporter 1*		**EIF2AK2**	*eukaryotic translation initiation factor 2-alpha kinase 2*	
**CTD-2267D19.3**	*Uncharacterized protein*		**SLC9B2**	*solute carrier family 9, subfamily B (NHA2, cation proton antiporter 2), member 2*	
**SETD5**	*SET domain containing 5*	✓	**GOLIM4**	*golgi integral membrane protein 4*	
**CTDSPL2**	*CTD (carboxy-terminal domain, RNA polymerase II, polypeptide A) small phosphatase like 2*		**RBM12B**	*RNA binding motif protein 12B*	

**CFHR3**	*complement factor H-related 3*		**C11orf45**	*chromosome 11 open reading frame 45*	
**CLEC6A**	*C-type lectin domain family 6, member A*		**TCEB1**	*transcription elongation factor B (SIII), polypeptide 1 (15kDa, elongin C)*	
**SLC2A13**	*solute carrier family 2 (facilitated glucose transporter), member 13*		**ADH5**	*alcohol dehydrogenase 5 (class III), chi polypeptide*	
**ETV1**	*ets variant 1*		**IFI44L**	*interferon-induced protein 44-like*	
**CYP4F11**	*cytochrome P450, family 4, subfamily F, polypeptide 11*		**NRG1**	*neuregulin 1*	
**ANKRD29**	*ankyrin repeat domain 29*		**IL16**	*interleukin 16*	
**ANGPT2**	*angiopoietin 2*		**SON**	*SON DNA binding protein*	✓
**C2orf91**	*chromosome 2 open reading frame 91*		**ANKRD12**	*ankyrin repeat domain 12*	
**GOLGA5**	*golgin A5*		**EXOSC2**	*exosome component 2*	
**WAPAL**	*wings apart-like homolog (Drosophila)*		**P2RY2**	*purinergic receptor P2Y, G-protein coupled, 2*	
**MINA**	*MYC induced nuclear antigen*		**CYP20A1**	*cytochrome P450, family 20, subfamily A, polypeptide 1*	
**GABRA6**	*gamma-aminobutyric acid (GABA) A receptor, alpha 6*		**EIF3F**	*eukaryotic translation initiation factor 3, subunit F*	
**TENM1**	*teneurin transmembrane protein 1*		**CCNT1**	*cyclin T1*	
**AC011755.1**	*HCG2006742; Protein LOC100996685*		**FAM126A**	*family with sequence similarity 126, member A*	
**TTYH3**	*tweety family member 3*		**ZBTB14**	*zinc finger and BTB domain containing 14*	
**DYNLT3**	*dynein, light chain, Tctex-type 3*		**LGALSL**	*lectin, galactoside-binding-like*	
**TOX4**	*TOX high mobility group box family member 4*		**MMP16**	*matrix metallopeptidase 16 (membrane-inserted)*	
**UBE2E3**	*ubiquitin-conjugating enzyme E2E 3*		**LARP4**	*La ribonucleoprotein domain family, member 4*	
**FBXO17**	*F-box protein 17*		**MYPN**	*myopalladin*	
**AC011897.1**	*Uncharacterized protein*		**FLG2**	*filaggrin family member 2*	
**FBXW11**	*F-box and WD repeat domain containing 11*		**MED1**	*mediator complex subunit 1*	
**EIF4E3**	*eukaryotic translation initiation factor 4E family member 3*		**SOGA3**	*SOGA family member 3*	
**IKBKG**	*inhibitor of kappa light polypeptide gene enhancer in B-cells, kinase gamma*		**ADAT2**	*adenosine deaminase, tRNA-specific 2*	
**HSD17B11**	*hydroxysteroid (17-beta) dehydrogenase 11*		**TRIM59**	*tripartite motif containing 59*	

**CTSO**	*cathepsin O*		**PAPOLG**	*poly(A) polymerase gamma*	
**CXorf36**	*chromosome X open reading frame 36*		**C5orf63**	*chromosome 5 open reading frame 63*	
**FOCAD**	*focadhesin*		**NME6**	*NME/NM23 nucleoside diphosphate kinase 6*	
**PRLR**	*prolactin receptor*	✓	**GMNC**	*geminin coiled-coil domain containing*	
**JAG1**	*jagged 1*		**MOCS2**	*molybdenum cofactor synthesis 2*	
**TAS2R20**	*taste receptor, type 2, member 20*		**UBB**	*ubiquitin B*	
**ZNF229**	*zinc finger protein 229*		**TMEM186**	*transmembrane protein 186*	
**HPS3**	*Hermansky-Pudlak syndrome 3*		**FBXL13**	*F-box and leucine-rich repeat protein 13*	
**MXI1**	*MAX interactor 1, dimerization protein*		**PLXDC2**	*plexin domain containing 2*	
**FEM1C**	*fem-1 homolog c (C. elegans)*	✓	**FGF12**	*fibroblast growth factor 12*	
**BIRC3**	*baculoviral IAP repeat containing 3*		**ZDHHC17**	*zinc finger, DHHC-type containing 17*	
**TWIST1**	*twist basic helix-loop-helix transcription factor 1*		**GNAI1**	*guanine nucleotide binding protein (G protein), alpha inhibiting activity polypeptide 1*	
**LYRM7**	*LYR motif containing 7*		**WDHD1**	*WD repeat and HMG-box DNA binding protein 1*	
**MIS18BP1**	*MIS18 binding protein 1*		**PTPRT**	*protein tyrosine phosphatase, receptor type, T*	
**ZNF451**	*zinc finger protein 451*		**SLCO5A1**	*solute carrier organic anion transporter family, member 5A1*	
**CDH19**	*cadherin 19, type 2*		**JARID2**	*jumonji, AT rich interactive domain 2*	
**MEDAG**	*mesenteric estrogen-dependent adipogenesis*		**B3GALT5**	*UDP-Gal:betaGlcNAc beta 1,3-galactosyltransferase, polypeptide 5*	
**CCDC102B**	*coiled-coil domain containing 102B*		**SGSM1**	*small G protein signaling modulator 1*	
**GSDMA**	*gasdermin A*		**ARHGAP19**	*Rho GTPase activating protein 19*	
**NOV**	*nephroblastoma overexpressed*		**ARHGEF15**	*Rho guanine nucleotide exchange factor (GEF) 15*	
**FZD7**	*frizzled family receptor 7*		**PHLPP2**	*PH domain and leucine rich repeat protein phosphatase 2*	
**ZNF30**	*zinc finger protein 30*		**PKNOX2**	*PBX/knotted 1 homeobox 2*	
**ZBTB32**	*zinc finger and BTB domain containing 32*		**LUC7L3**	*LUC7-like 3 (S. cerevisiae)*	
**SRPK2**	*SRSF protein kinase 2*		**NFASC**	*neurofascin*	

**PDXDC1**	*pyridoxal-dependent decarboxylase domain containing 1*		**ANK3**	*ankyrin 3, node of Ranvier (ankyrin G)*	
**CAPRIN1**	*cell cycle associated protein 1*		**CLCF1**	*cardiotrophin-like cytokine factor 1*	
**TBC1D15**	*TBC1 domain family, member 15*		**SLC2A10**	*solute carrier family 2 (facilitated glucose transporter), member 10*	
**CCDC91**	*coiled-coil domain containing 91*		**MAB21L3**	*mab-21-like 3 (C. elegans)*	
**USP33**	*ubiquitin specific peptidase 33*		**PALM**	*paralemmin*	
**INSL3**	*insulin-like 3 (Leydig cell)*		**SFXN5**	*sideroflexin 5*	
**DDB2**	*damage-specific DNA binding protein 2, 48kDa*		**RASGRF1**	*Ras protein-specific guanine nucleotide-releasing factor 1*	
**TWF1**	*twinfilin actin-binding protein 1*		**MEF2C**	*myocyte enhancer factor 2C*	
**ZBTB49**	*zinc finger and BTB domain containing 49*		**TTBK1**	*tau tubulin kinase 1*	
**STYX**	*serine/threonine/tyrosine interacting protein*		**GHR**	*growth hormone receptor*	
**ALAD**	*aminolevulinate dehydratase*		**XKR7**	*XK, Kell blood group complex subunit-related family, member 7*	
**CDKL1**	*cyclin-dependent kinase-like 1 (CDC2-related kinase)*		**ANKRD52**	*ankyrin repeat domain 52*	
**WSB2**	*WD repeat and SOCS box containing 2*	✓	**KIAA2022**	*KIAA2022*	
**FAM117A**	*family with sequence similarity 117, member A*		**PCDH17**	*protocadherin 17*	
**PLCXD3**	*phosphatidylinositol-specific phospholipase C, X domain containing 3*		**KBTBD8**	*kelch repeat and BTB (POZ) domain containing 8*	
**AGXT2**	*alanine--glyoxylate aminotransferase 2*		**RANBP10**	*RAN binding protein 10*	
**ERRFI1**	*ERBB receptor feedback inhibitor 1*		**RASAL2**	*RAS protein activator like 2*	
**RNF180**	*ring finger protein 180*		**CLIP3**	*CAP-GLY domain containing linker protein 3*	
**CRTC3**	*CREB regulated transcription coactivator 3*		**ARHGEF5**	*Rho guanine nucleotide exchange factor (GEF) 5*	
**ULBP1**	*UL16 binding protein 1*		**CAMK4**	*calcium/calmodulin-dependent protein kinase IV*	
**TGM4**	*transglutaminase 4*		**SUB1**	*SUB1 homolog (S. cerevisiae)*	
**LRP2BP**	*LRP2 binding protein*		**MTR**	*5-methyltetrahydrofolate-homocysteine methyltransferase*	
**RAD9B**	*RAD9 homolog B (S. pombe)*		**SNX13**	*sorting nexin 13*	
**AQP4**	*aquaporin 4*		**TRIM36**	*tripartite motif containing 36*	

**TRIM4**	*tripartite motif containing 4*		**GPD2**	*glycerol-3-phosphate dehydrogenase 2 (mitochondrial)*	
**ZNF26**	*zinc finger protein 26*		**TMCC3**	*transmembrane and coiled-coil domain family 3*	
**KCNK15**	*potassium channel, subfamily K, member 15*		**SEPT11**	*septin 11*	
**DNAJC10**	*DnaJ (Hsp40) homolog, subfamily C, member 10*		**KLB**	*klotho beta*	
**NARS**	*asparaginyl-tRNA synthetase*	✓	**OTUD7B**	*OTU domain containing 7B*	
**LRRC55**	*leucine rich repeat containing 55*		**ARHGAP32**	*Rho GTPase activating protein 32*	
**APOBEC2**	*apolipoprotein B mRNA editing enzyme, catalytic polypeptide-like 2*		**EPB41L5**	*erythrocyte membrane protein band 4.1 like 5*	
**BCAS1**	*breast carcinoma amplified sequence 1*		**BBX**	*bobby sox homolog (Drosophila)*	
**SSFA2**	*sperm specific antigen 2*		**PRKCE**	*protein kinase C, epsilon*	
**ZNF625**	*zinc finger protein 625*		**IQCB1**	*IQ motif containing B1*	
**G2E3**	*G2/M-phase specific E3 ubiquitin protein ligase*		**DCAF4L1**	*DDB1 and CUL4 associated factor 4-like 1*	
**LNX2**	*ligand of numb-protein X 2*		**SMC2**	*structural maintenance of chromosomes 2*	
**RBBP9**	*retinoblastoma binding protein 9*		**C2orf68**	*chromosome 2 open reading frame 68*	
**R3HCC1**	*R3H domain and coiled-coil containing 1*		**WHSC1L1**	*Wolf-Hirschhorn syndrome candidate 1-like 1*	
**MTFR1**	*mitochondrial fission regulator 1*		**RABEP1**	*rabaptin, RAB GTPase binding effector protein 1*	
**TMEM143**	*transmembrane protein 143*		**RNF217**	*ring finger protein 217*	
**MARCKS**	*myristoylated alanine-rich protein kinase C substrate*		**PPP4R1L**	*protein phosphatase 4, regulatory subunit 1-like*	
**FBXL4**	*F-box and leucine-rich repeat protein 4*		**PNPLA4**	*patatin-like phospholipase domain containing 4*	
**SLC10A2**	*solute carrier family 10 (sodium/bile acid cotransporter), member 2*		**CDK16**	*cyclin-dependent kinase 16*	
**PPAP2B**	*phosphatidic acid phosphatase type 2B*		**KPNA4**	*karyopherin alpha 4 (importin alpha 3)*	
**COMMD10**	*COMM domain containing 10*		**CENPN**	*centromere protein N*	
**KIAA1715**	*KIAA1715*		**GAPVD1**	*GTPase activating protein and VPS9 domains 1*	
**ZNF677**	*zinc finger protein 677*		**ZHX3**	*zinc fingers and homeoboxes 3*	
**ZNF141**	*zinc finger protein 141*		**AP5B1**	*adaptor-related protein complex 5, beta 1 subunit*	

**SLC35G1**	*solute carrier family 35, member G1*		**BID**	*BH3 interacting domain death agonist*	
**ERBB3**	*v-erb-b2 avian erythroblastic leukemia viral oncogene homolog 3*		**SPRED2**	*sprouty-related, EVH1 domain containing 2*	
**ATG4A**	*autophagy related 4A, cysteine peptidase*		**LUZP1**	*leucine zipper protein 1*	
**BX088651.1**	*LOC100126582 protein; Uncharacterized protein*		**RAB11FIP2**	*RAB11 family interacting protein 2 (class I)*	
**STAT1**	*signal transducer and activator of transcription 1, 91kDa*		**INPP4A**	*inositol polyphosphate-4-phosphatase, type I, 107kDa*	
**GRAP2**	*GRB2-related adaptor protein 2*		**UQCC1**	*ubiquinol-cytochrome c reductase complex assembly factor 1*	
**LMO7**	*LIM domain 7*		**SLC20A2**	*solute carrier family 20 (phosphate transporter), member 2*	
**TRH**	*thyrotropin-releasing hormone*		**PTER**	*phosphotriesterase related*	
**HNRNPA1**	*heterogeneous nuclear ribonucleoprotein A1*		**ARL5B**	*ADP-ribosylation factor-like 5B*	
**SERAC1**	*serine active site containing 1*		**API5**	*apoptosis inhibitor 5*	
**NAMPT**	*nicotinamide phosphoribosyltransferase*		**BDH1**	*3-hydroxybutyrate dehydrogenase, type 1*	
**CEP104**	*centrosomal protein 104kDa*		**DYNLL2**	*dynein, light chain, LC8-type 2*	
**ARID5B**	*AT rich interactive domain 5B (MRF1-like)*		**SFT2D2**	*SFT2 domain containing 2*	
**STX2**	*syntaxin 2*		**SHANK2**	*SH3 and multiple ankyrin repeat domains 2*	
**CCDC115**	*coiled-coil domain containing 115*		**TUBGCP4**	*tubulin, gamma complex associated protein 4*	
**DUSP13**	*dual specificity phosphatase 13*		**IGF1**	*insulin-like growth factor 1 (somatomedin C)*	
**DHODH**	*dihydroorotate dehydrogenase (quinone)*		**ZNF138**	*zinc finger protein 138*	
**ATXN7L3B**	*ataxin 7-like 3B*		**MSL2**	*male-specific lethal 2 homolog (Drosophila)*	
**THOC7**	*THO complex 7 homolog (Drosophila)*		**STIM2**	*stromal interaction molecule 2*	
**ZNF468**	*zinc finger protein 468*		**SLC7A6OS**	*solute carrier family 7, member 6 opposite strand*	
**BLOC1S6**	*biogenesis of lysosomal organelles complex-1, subunit 6, pallidin*		**ZNF20**	*zinc finger protein 20*	
**RAP2B**	*RAP2B, member of RAS oncogene family*		**PAQR8**	*progestin and adipoQ receptor family member VIII*	
**ARL6**	*ADP-ribosylation factor-like 6*		**FBXO22**	*F-box protein 22*	
**DIO2**	*deiodinase, iodothyronine, type II*		**APPBP2**	*amyloid beta precursor protein (cytoplasmic tail) binding protein 2*	

**HMGA1**	*high mobility group AT-hook 1*		**CREG2**	*cellular repressor of E1A-stimulated genes 2*	
**CCDC38**	*coiled-coil domain containing 38*		**TWISTNB**	*TWIST neighbor*	
**ABHD6**	*abhydrolase domain containing 6*		**TADA2B**	*transcriptional adaptor 2B*	
**AKAP6**	*A kinase (PRKA) anchor protein 6*		**TRIM66**	*tripartite motif containing 66*	
**CHRM2**	*cholinergic receptor, muscarinic 2*		**YIPF6**	*Yip1 domain family, member 6*	
**KCNG3**	*potassium voltage-gated channel, subfamily G, member 3*		**MAML2**	*mastermind-like 2 (Drosophila)*	
**CACNA2D1**	*calcium channel, voltage-dependent, alpha 2/delta subunit 1*		**HDAC4**	*histone deacetylase 4*	
**TMTC3**	*transmembrane and tetratricopeptide repeat containing 3*		**FNBP1L**	*formin binding protein 1-like*	
**LRRTM2**	*leucine rich repeat transmembrane neuronal 2*		**GPR180**	*G protein-coupled receptor 180*	
**AGAP10**	*ArfGAP with GTPase domain, ankyrin repeat and PH domain 10*		**MRE11A**	*MRE11 meiotic recombination 11 homolog A (S. cerevisiae)*	
**GALNT15**	*UDP-N-acetyl-alpha-D-galactosamine:polypeptide N-acetylgalactosaminyltransferase 15*		**EIF4EBP3**	*eukaryotic translation initiation factor 4E binding protein 3*	
**MDM2**	*MDM2 oncogene, E3 ubiquitin protein ligase*		**DHRS7**	*dehydrogenase/reductase (SDR family) member 7*	
**TP63**	*tumor protein p63*		**TNRC6B**	*trinucleotide repeat containing 6B*	
**SPAG9**	*sperm associated antigen 9*		**PCNX**	*pecanex homolog (Drosophila)*	
**AGAP9**	*ArfGAP with GTPase domain, ankyrin repeat and PH domain 9*		**KLHL6**	*kelch-like family member 6*	
**BCOR**	*BCL6 corepressor*		**KIAA1033**	*KIAA1033*	
**UNC13C**	*unc-13 homolog C (C. elegans)*		**HS6ST3**	*heparan sulfate 6-O-sulfotransferase 3*	
**DCAF8L1**	*DDB1 and CUL4 associated factor 8-like 1*		**EIF1**	*eukaryotic translation initiation factor 1*	
**KPNA1**	*karyopherin alpha 1 (importin alpha 5)*		**CALCRL**	*calcitonin receptor-like*	
**FGF7**	*fibroblast growth factor 7*		**RRAGD**	*Ras-related GTP binding D*	
**SLC36A2**	*solute carrier family 36 (proton/amino acid symporter), member 2*		**ERBB4**	*v-erb-b2 avian erythroblastic leukemia viral oncogene homolog 4*	
**KCNJ15**	*potassium inwardly-rectifying channel, subfamily J, member 15*		**SCAI**	*suppressor of cancer cell invasion*	
**ENTPD5**	*ectonucleoside triphosphate diphosphohydrolase 5*		**KDM5A**	*lysine (K)-specific demethylase 5A*	
**PALLD**	*palladin, cytoskeletal associated protein*		**AMER2**	*APC membrane recruitment protein 2*	

**KLF3**	*Kruppel-like factor 3 (basic)*		**RBM7**	*RNA binding motif protein 7*	
**GAB1**	*GRB2-associated binding protein 1*		**PCDH18**	*protocadherin 18*	
**UBASH3B**	*ubiquitin associated and SH3 domain containing B*		**KCTD20**	*potassium channel tetramerization domain containing 20*	
**VWA3A**	*von Willebrand factor A domain containing 3A*		**TMEM200C**	*transmembrane protein 200C*	
**AC090616.2**	*Uncharacterized protein*		**PRPF40A**	*PRP40 pre-mRNA processing factor 40 homolog A (S. cerevisiae)*	
**EIF1AY**	*eukaryotic translation initiation factor 1A, Y-linked*		**ZC3H13**	*zinc finger CCCH-type containing 13*	
**MSMO1**	*methylsterol monooxygenase 1*		**PPP4R4**	*protein phosphatase 4, regulatory subunit 4*	
**GPHN**	*gephyrin*		**IL20RA**	*interleukin 20 receptor, alpha*	
**ATP6V1G2**	*ATPase, H+ transporting, lysosomal 13kDa, V1 subunit G2*		**MTO1**	*mitochondrial tRNA translation optimization 1*	
**PTEN**	*phosphatase and tensin homolog*		**DENND5B**	*DENN/MADD domain containing 5B*	
**RBM48**	*RNA binding motif protein 48*		**ZNF681**	*zinc finger protein 681*	
**HLTF**	*helicase-like transcription factor*		**SPATA17**	*spermatogenesis associated 17*	
**BTC**	*betacellulin*		**UBE2D4**	*ubiquitin-conjugating enzyme E2D 4 (putative)*	
**LUZPP1**	*HCG1777807; Leucine zipper protein 3; Uncharacterized protein*		**ATP8A2**	*ATPase, aminophospholipid transporter, class I, type 8A, member 2*	
**SLC4A7**	*solute carrier family 4, sodium bicarbonate cotransporter, member 7*		**LYRM9**	*LYR motif containing 9*	
**TBCK**	*TBC1 domain containing kinase*		**SPATA6**	*spermatogenesis associated 6*	
**MBTPS2**	*membrane-bound transcription factor peptidase, site 2*		**CEP57L1**	*centrosomal protein 57kDa-like 1*	
**FAM122B**	*family with sequence similarity 122B*		**SYT7**	*synaptotagmin VII*	
**MTDH**	*metadherin*		**ZNF324B**	*zinc finger protein 324B*	
**OMD**	*osteomodulin*		**MYO1F**	*myosin IF*	
**TMEM155**	*transmembrane protein 155*		**COMMD2**	*COMM domain containing 2*	
**LEKR1**	*leucine, glutamate and lysine rich 1*		**HNMT**	*histamine N-methyltransferase*	
**RECQL**	*RecQ protein-like (DNA helicase Q1-like)*		**GNL3L**	*guanine nucleotide binding protein-like 3 (nucleolar)-like*	
**PLA2R1**	*phospholipase A2 receptor 1, 180kDa*		**MESDC2**	*mesoderm development candidate 2*	

**SMIM13**	*small integral membrane protein 13*		**ZNF622**	*zinc finger protein 622*	
**SMUG1**	*single-strand-selective monofunctional uracil-DNA glycosylase 1*		**SLC4A8**	*solute carrier family 4, sodium bicarbonate cotransporter, member 8*	
**ORMDL1**	*ORM1-like 1 (S. cerevisiae)*		**A1CF**	*APOBEC1 complementation factor*	
**RGS17**	*regulator of G-protein signaling 17*		**FOXL1**	*forkhead box L1*	
**RPUSD2**	*RNA pseudouridylate synthase domain containing 2*		**FBXO11**	*F-box protein 11*	
**LRRC23**	*leucine rich repeat containing 23*		**SUGT1**	*SGT1, suppressor of G2 allele of SKP1 (S. cerevisiae)*	
**EPT1**	*ethanolaminephosphotransferase 1 (CDP-ethanolamine-specific)*		**TTC33**	*tetratricopeptide repeat domain 33*	
**SERPINA10**	*serpin peptidase inhibitor, clade A (alpha-1 antiproteinase, antitrypsin), member 10*		**DDHD1**	*DDHD domain containing 1*	
**LHFPL2**	*lipoma HMGIC fusion partner-like 2*		**TRMT5**	*tRNA methyltransferase 5*	
**MYO5B**	*myosin VB*		**CCDC30**	*coiled-coil domain containing 30*	
**PLAG1**	*pleiomorphic adenoma gene 1*		**RHCG**	*Rh family, C glycoprotein*	
**KBTBD3**	*kelch repeat and BTB (POZ) domain containing 3*		**LRRC47**	*leucine rich repeat containing 47*	
**SYT11**	*synaptotagmin XI*		**TFAM**	*transcription factor A, mitochondrial*	
**CMKLR1**	*chemokine-like receptor 1*		**RPL9**	*ribosomal protein L9*	
**ZKSCAN3**	*zinc finger with KRAB and SCAN domains 3*		**DGKH**	*diacylglycerol kinase, eta*	
**AK9**	*adenylate kinase 9*		**WBP11**	*WW domain binding protein 11*	
**ZBTB37**	*zinc finger and BTB domain containing 37*		**NLGN1**	*neuroligin 1*	
**ARMC1**	*armadillo repeat containing 1*		**LAMC2**	*laminin, gamma 2*	
**CARD8**	*caspase recruitment domain family, member 8*		**NUMA1**	*nuclear mitotic apparatus protein 1*	
**C10orf53**	*chromosome 10 open reading frame 53*		**NOL4**	*nucleolar protein 4*	
**HDGFL1**	*hepatoma derived growth factor-like 1*		**DNAL1**	*dynein, axonemal, light chain 1*	
**C16orf72**	*chromosome 16 open reading frame 72*		**DYRK2**	*dual-specificity tyrosine-(Y)-phosphorylation regulated kinase 2*	
**AFF1**	*AF4/FMR2 family, member 1*		**MLANA**	*melan-A*	
**DNAJC3**	*DnaJ (Hsp40) homolog, subfamily C, member 3*		**ITPRIP**	*inositol 1,4,5-trisphosphate receptor interacting protein*	

**GPBP1**	*GC-rich promoter binding protein 1*	✓	**LATS1**	*large tumor suppressor kinase 1*	
**RALGPS2**	*Ral GEF with PH domain and SH3 binding motif 2*		**LRIG3**	*leucine-rich repeats and immunoglobulin-like domains 3*	
**MAU2**	*MAU2 sister chromatid cohesion factor*		**TMEM50B**	*transmembrane protein 50B*	
**DOK6**	*docking protein 6*		**ZNF529**	*zinc finger protein 529*	✓
**C6ORF165**	*UPF0704 protein C6orf165*		**ITPR2**	*inositol 1,4,5-trisphosphate receptor, type 2*	
**HSF2**	*heat shock transcription factor 2*		**NIPSNAP3B**	*nipsnap homolog 3B (C. elegans)*	
**ZNF365**	*zinc finger protein 365*		**DMXL1**	*Dmx-like 1*	
**NKX2-4**	*NK2 homeobox 4*		**PPM1L**	*protein phosphatase, Mg2+/Mn2+ dependent, 1L*	
**DENND6A**	*DENN/MADD domain containing 6A*		**TRIM38**	*tripartite motif containing 38*	
**FKBP10**	*FK506 binding protein 10, 65 kDa*		**CAPN7**	*calpain 7*	
**TOR1B**	*torsin family 1, member B (torsin B)*		**ATXN10**	*ataxin 10*	
**IFIT3**	*interferon-induced protein with tetratricopeptide repeats 3*		**PTPN3**	*protein tyrosine phosphatase, non-receptor type 3*	
**ETNK2**	*ethanolamine kinase 2*		**MTMR7**	*myotubularin related protein 7*	
**BTAF1**	*BTAF1 RNA polymerase II, B-TFIID transcription factor-associated, 170kDa*		**GC**	*group-specific component (vitamin D binding protein)*	
**ZG16**	*zymogen granule protein 16*		**MTPAP**	*mitochondrial poly(A) polymerase*	
**PPP1R14C**	*protein phosphatase 1, regulatory (inhibitor) subunit 14C*		**CBLN1**	*cerebellin 1 precursor*	
**SEPT2**	*septin 2*		**CNOT6**	*CCR4-NOT transcription complex, subunit 6*	
**ZCCHC2**	*zinc finger, CCHC domain containing 2*		**GUCY1A2**	*guanylate cyclase 1, soluble, alpha 2*	
**C17orf77**	*chromosome 17 open reading frame 77*		**PCCB**	*propionyl CoA carboxylase, beta polypeptide*	
**SNAPC3**	*small nuclear RNA activating complex, polypeptide 3, 50kDa*		**MRPL3**	*mitochondrial ribosomal protein L3*	
**ZNF728**	*zinc finger protein 728*		**BHLHE41**	*basic helix-loop-helix family, member e41*	
**CUL5**	*cullin 5*		**ORC4**	*origin recognition complex, subunit 4*	
**PURG**	*purine-rich element binding protein G*		**UHRF1BP1L**	*UHRF1 binding protein 1-like*	
**ARHGEF38**	*Rho guanine nucleotide exchange factor (GEF) 38*		**TFDP2**	*transcription factor Dp-2 (E2F dimerization partner 2)*	

**LMX1A**	*LIM homeobox transcription factor 1, alpha*		**ZBTB40**	*zinc finger and BTB domain containing 40*	
**GK5**	*glycerol kinase 5 (putative)*		**WDR35**	*WD repeat domain 35*	
**ZNF521**	*zinc finger protein 521*		**ZNF354B**	*zinc finger protein 354B*	
**SIX3**	*SIX homeobox 3*		**UBA2**	*ubiquitin-like modifier activating enzyme 2*	
**B4GALT6**	*UDP-Gal:betaGlcNAc beta 1,4- galactosyltransferase, polypeptide 6*		**COA5**	*cytochrome c oxidase assembly factor 5*	
**AGAP5**	*ArfGAP with GTPase domain, ankyrin repeat and PH domain 5*		**TNPO3**	*transportin 3*	
**ZNF562**	*zinc finger protein 562*		**THSD4**	*thrombospondin, type I, domain containing 4*	
**ZMAT3**	*zinc finger, matrin-type 3*		**CISD1**	*CDGSH iron sulfur domain 1*	
**MBP**	*myelin basic protein*		**SMIM12**	*small integral membrane protein 12*	
**GPR110**	*G protein-coupled receptor 110*		**ABLIM3**	*actin binding LIM protein family, member 3*	
**ASPH**	*aspartate beta-hydroxylase*		**SOD2**	*superoxide dismutase 2, mitochondrial*	
**NKTR**	*natural killer-tumor recognition sequence*		**RBM4B**	*RNA binding motif protein 4B*	
**UBE2H**	*ubiquitin-conjugating enzyme E2H*		**IMPG2**	*interphotoreceptor matrix proteoglycan 2*	
**PIK3R1**	*phosphoinositide-3-kinase, regulatory subunit 1 (alpha)*		**PLEKHM3**	*pleckstrin homology domain containing, family M, member 3*	
**WASL**	*Wiskott-Aldrich syndrome-like*		**LNPEP**	*leucyl/cystinyl aminopeptidase*	
**FCGR3B**	*Fc fragment of IgG, low affinity IIIb, receptor (CD16b)*		**NUP43**	*nucleoporin 43kDa*	
**EYS**	*eyes shut homolog (Drosophila)*		**SEC63**	*SEC63 homolog (S. cerevisiae)*	
**TMEM174**	*transmembrane protein 174*		**FRMD4A**	*FERM domain containing 4A*	
**EFCAB14**	*EF-hand calcium binding domain 14*		**AP3M2**	*adaptor-related protein complex 3, mu 2 subunit*	
**SULT2A1**	*sulfotransferase family, cytosolic, 2A, dehydroepiandrosterone (DHEA)-preferring, member 1*		**CA5B**	*carbonic anhydrase VB, mitochondrial*	
**LRRTM3**	*leucine rich repeat transmembrane neuronal 3*		**POLR3D**	*polymerase (RNA) III (DNA directed) polypeptide D, 44kDa*	
**B3GNT2**	*UDP-GlcNAc:betaGal beta-1,3-N-acetylglucosaminyltransferase 2*		**ISPD**	*isoprenoid synthase domain containing*	
**VPS33B**	*vacuolar protein sorting 33 homolog B (yeast)*		**CBX5**	*chromobox homolog 5*	

**LYSMD2**	*LysM, putative peptidoglycan-binding, domain containing 2*		**FLRT2**	*fibronectin leucine rich transmembrane protein 2*	
**PIGK**	*phosphatidylinositol glycan anchor biosynthesis, class K*		**SESTD1**	*SEC14 and spectrin domains 1*	
**KCNJ16**	*potassium inwardly-rectifying channel, subfamily J, member 16*		**FBXW2**	*F-box and WD repeat domain containing 2*	
**EXOC1**	*exocyst complex component 1*		**PMPCA**	*peptidase (mitochondrial processing) alpha*	
**PMFBP1**	*polyamine modulated factor 1 binding protein 1*		**PHLDB1**	*pleckstrin homology-like domain, family B, member 1*	
**SPRY3**	*sprouty homolog 3 (Drosophila)*		**UGT3A1**	*UDP glycosyltransferase 3 family, polypeptide A1*	
**ALG6**	*ALG6, alpha-1,3-glucosyltransferase*		**TTL**	*tubulin tyrosine ligase*	
**MSI2**	*musashi RNA-binding protein 2*		**LRP12**	*low density lipoprotein receptor-related protein 12*	
**PGGT1B**	*protein geranylgeranyltransferase type I, beta subunit*		**NFAT5**	*nuclear factor of activated T-cells 5, tonicity-responsive*	
**CNKSR2**	*connector enhancer of kinase suppressor of Ras 2*		**KLHL5**	*kelch-like family member 5*	
**FMOD**	*fibromodulin*		**VPS37A**	*vacuolar protein sorting 37 homolog A (S. cerevisiae)*	
**METTL13**	*methyltransferase like 13*		**ORC1**	*origin recognition complex, subunit 1*	
**AGAP7**	*ArfGAP with GTPase domain, ankyrin repeat and PH domain 7*		**POLR3E**	*polymerase (RNA) III (DNA directed) polypeptide E (80kD)*	✓
**AGAP8**	*ArfGAP with GTPase domain, ankyrin repeat and PH domain 8*		**CCDC109B**	*coiled-coil domain containing 109B*	
**TRPC4**	*transient receptor potential cation channel, subfamily C, member 4*		**USP9X**	*ubiquitin specific peptidase 9, X-linked*	
**FNBP1**	*formin binding protein 1*		**GAB3**	*GRB2-associated binding protein 3*	
**PNMA1**	*paraneoplastic Ma antigen 1*		**INO80D**	*INO80 complex subunit D*	
**NUBPL**	*nucleotide binding protein-like*		**SCN2A**	*sodium channel, voltage-gated, type II, alpha subunit*	
**NCBP2**	*nuclear cap binding protein subunit 2, 20kDa*		**MED12L**	*mediator complex subunit 12-like*	
**PAX8**	*paired box 8*		**RPL23**	*ribosomal protein L23*	
**KLHL31**	*kelch-like family member 31*		**C2orf69**	*chromosome 2 open reading frame 69*	
**ANKRD20A2**	*ankyrin repeat domain 20 family, member A2*		**PDZD2**	*PDZ domain containing 2*	
**AGAP6**	*ArfGAP with GTPase domain, ankyrin repeat and PH domain 6*		**BICD2**	*bicaudal D homolog 2 (Drosophila)*	
**PDHX**	*pyruvate dehydrogenase complex, component X*		**CTNNA3**	*catenin (cadherin-associated protein), alpha 3*	

**ANKRD20A1**	*ankyrin repeat domain 20 family, member A1*		**LIPG**	*lipase, endothelial*	
**C15orf54**	*chromosome 15 open reading frame 54*		**LIN28B**	*lin-28 homolog B (C. elegans)*	
**MITF**	*microphthalmia-associated transcription factor*		**HS6ST2**	*heparan sulfate 6-O-sulfotransferase 2*	
**APOB**	*apolipoprotein B*		**EVC**	*Ellis van Creveld syndrome*	
**B3GNT9**	*UDP-GlcNAc:betaGal beta-1,3-N-acetylglucosaminyltransferase 9*		**ASTN1**	*astrotactin 1*	
**CXCL9**	*chemokine (C-X-C motif) ligand 9*		**KCNJ10**	*potassium inwardly-rectifying channel, subfamily J, member 10*	
**ARHGAP18**	*Rho GTPase activating protein 18*		**PCDH19**	*protocadherin 19*	
**METTL9**	*methyltransferase like 9*		**PDGFRA**	*platelet-derived growth factor receptor, alpha polypeptide*	
**GPR64**	*G protein-coupled receptor 64*		**SLC26A2**	*solute carrier family 26 (anion exchanger), member 2*	
**BEND4**	*BEN domain containing 4*		**C20orf194**	*chromosome 20 open reading frame 194*	
**AGAP4**	*ArfGAP with GTPase domain, ankyrin repeat and PH domain 4*		**RSPH3**	*radial spoke 3 homolog (Chlamydomonas)*	
**RAB5A**	*RAB5A, member RAS oncogene family*		**AGO1**	*argonaute RISC catalytic component 1*	
**HNRNPH2**	*heterogeneous nuclear ribonucleoprotein H2 (H′)*		**FRMD4B**	*FERM domain containing 4B*	
**PAN2**	*PAN2 poly(A) specific ribonuclease subunit homolog (S. cerevisiae)*		**RASA2**	*RAS p21 protein activator 2*	
**C10orf10**	*chromosome 10 open reading frame 10*		**TMEM154**	*transmembrane protein 154*	
**CD38**	*CD38 molecule*		**OCLN**	*occludin*	
**SIK1**	*salt-inducible kinase 1*		**TSPAN11**	*tetraspanin 11*	✓
**STOML3**	*stomatin (EPB72)-like 3*		**CSMD2**	*CUB and Sushi multiple domains 2*	
**FAM71C**	*family with sequence similarity 71, member C*		**UNC79**	*unc-79 homolog (C. elegans)*	
**ZNF2**	*zinc finger protein 2*		**ETV3**	*ets variant 3*	
**NDUFA5**	*NADH dehydrogenase (ubiquinone) 1 alpha subcomplex, 5*		**ZSCAN12**	*zinc finger and SCAN domain containing 12*	
**EDIL3**	*EGF-like repeats and discoidin I-like domains 3*		**CHL1**	*cell adhesion molecule L1-like*	
**TESK2**	*testis-specific kinase 2*		**NPFFR1**	*neuropeptide FF receptor 1*	
**MTRF1L**	*mitochondrial translational release factor 1-like*		**KIT**	*v-kit Hardy-Zuckerman 4 feline sarcoma viral oncogene homolog*	

**PRR18**	*proline rich 18*		**RRP7A**	*ribosomal RNA processing 7 homolog A (S. cerevisiae)*	
**POM121C**	*POM121 transmembrane nucleoporin C*		**ZNF639**	*zinc finger protein 639*	
**NOS3**	*nitric oxide synthase 3 (endothelial cell)*		**EXTL3**	*exostosin-like glycosyltransferase 3*	
**ATPAF1**	*ATP synthase mitochondrial F1 complex assembly factor 1*		**HSPA12A**	*heat shock 70kDa protein 12A*	
**MYLIP**	*myosin regulatory light chain interacting protein*		**PCSK7**	*proprotein convertase subtilisin/kexin type 7*	
**COL14A1**	*collagen, type XIV, alpha 1*		**RABGAP1L**	*RAB GTPase activating protein 1-like*	
**BEND2**	*BEN domain containing 2*		**PIK3R3**	*phosphoinositide-3-kinase, regulatory subunit 3 (gamma)*	
**NRSN2**	*neurensin 2*		**TUSC2**	*tumor suppressor candidate 2*	
**SIRT2**	*sirtuin 2*		**BRWD1**	*bromodomain and WD repeat domain containing 1*	
**RASSF2**	*Ras association (RalGDS/AF-6) domain family member 2*		**MAP1LC3B**	*microtubule-associated protein 1 light chain 3 beta*	
**RPL36A-HNRNPH2**	*RPL36A-HNRNPH2 readthrough*		**CCDC138**	*coiled-coil domain containing 138*	
**AFF4**	*AF4/FMR2 family, member 4*		**SLC7A5**	*solute carrier family 7 (amino acid transporter light chain, L system), member 5*	
**BAAT**	*bile acid CoA: amino acid N-acyltransferase (glycine N-choloyltransferase)*		**ZKSCAN1**	*zinc finger with KRAB and SCAN domains 1*	
**LYPLA1**	*lysophospholipase I*		**MYO18A**	*myosin XVIIIA*	
**ZNF701**	*zinc finger protein 701*		**ADAMTS1**	*ADAM metallopeptidase with thrombospondin type 1 motif, 1*	
**COL6A5**	*collagen, type VI, alpha 5*		**BRD3**	*bromodomain containing 3*	
**FAM102B**	*family with sequence similarity 102, member B*		**WDR41**	*WD repeat domain 41*	
**FBXL3**	*F-box and leucine-rich repeat protein 3*		**MIPOL1**	*mirror-image polydactyly 1*	
**NF1**	*neurofibromin 1*		**FAM227A**	*family with sequence similarity 227, member A*	
**ELP3**	*elongator acetyltransferase complex subunit 3*		**RRP8**	*ribosomal RNA processing 8, methyltransferase, homolog (yeast)*	
**LACC1**	*laccase (multicopper oxidoreductase) domain containing 1*		**FBXO25**	*F-box protein 25*	
**DDX60L**	*DEAD (Asp-Glu-Ala-Asp) box polypeptide 60-like*		**QKI**	*QKI, KH domain containing, RNA binding*	
**PAQR9**	*progestin and adipoQ receptor family member IX*		**IRS4**	*insulin receptor substrate 4*	
**ZNF582**	*zinc finger protein 582*		**ADARB1**	*adenosine deaminase, RNA-specific, B1*	

**SH3RF2**	*SH3 domain containing ring finger 2*		**ZNF134**	*zinc finger protein 134*	
**STC1**	*stanniocalcin 1*		**ADCY7**	*adenylate cyclase 7*	
**BCAP29**	*B-cell receptor-associated protein 29*		**ELP2**	*elongator acetyltransferase complex subunit 2*	
**GNG4**	*guanine nucleotide binding protein (G protein), gamma 4*		**ZNF850**	*zinc finger protein 850*	
**MAPK1**	*mitogen-activated protein kinase 1*	✓	**GK**	*glycerol kinase*	
**UTRN**	*utrophin*		**CSNK2A1**	*casein kinase 2, alpha 1 polypeptide*	
**CNOT4**	*CCR4-NOT transcription complex, subunit 4*		**MAGI1**	*membrane associated guanylate kinase, WW and PDZ domain containing 1*	
**PRDM10**	*PR domain containing 10*		**EGFR**	*epidermal growth factor receptor*	
**LYPLAL1**	*lysophospholipase-like 1*		**LONRF3**	*LON peptidase N-terminal domain and ring finger 3*	
**IPP**	*intracisternal A particle-promoted polypeptide*		**DOCK11**	*dedicator of cytokinesis 11*	
**ITGA4**	*integrin, alpha 4 (antigen CD49D, alpha 4 subunit of VLA-4 receptor)*		**PDIA3**	*protein disulfide isomerase family A, member 3*	
**KAT2B**	*K(lysine) acetyltransferase 2B*		**PRDM6**	*PR domain containing 6*	
**IL1A**	*interleukin 1, alpha*		**TMED8**	*transmembrane emp24 protein transport domain containing 8*	
**HOXC13**	*homeobox C13*		**KIAA0391**	*KIAA0391*	
**TMEM236**			**NHLRC2**	*NHL repeat containing 2*	
**UBE2V1**	*ubiquitin-conjugating enzyme E2 variant 1*	✓	**ASTN2**	*astrotactin 2*	
**GPR171**	*G protein-coupled receptor 171*		**TPST2**	*tyrosylprotein sulfotransferase 2*	
**TMEM236**	*transmembrane protein 236*		**SESN3**	*sestrin 3*	✓
**EDN1**	*endothelin 1*		**ZKSCAN4**	*zinc finger with KRAB and SCAN domains 4*	
**BLOC1S5**	*biogenesis of lysosomal organelles complex-1, subunit 5, muted*		**CEP97**	*centrosomal protein 97kDa*	
**REV3L**	*REV3-like, polymerase (DNA directed), zeta, catalytic subunit*		**RTN2**	*reticulon 2*	
**ICT1**	*immature colon carcinoma transcript 1*		**TXNDC17**	*thioredoxin domain containing 17*	
**PRR23C**	*proline rich 23C*		**TECPR2**	*tectonin beta-propeller repeat containing 2*	
**TBCA**	*tubulin folding cofactor A*		**PLCB4**	*phospholipase C, beta 4*	

**CHTOP**	*chromatin target of PRMT1*		**PXMP4**	*peroxisomal membrane protein 4, 24kDa*	
**ZC3HAV1L**	*zinc finger CCCH-type, antiviral 1-like*	✓	**NT5DC1**	*5′-nucleotidase domain containing 1*	
**EML6**	*echinoderm microtubule associated protein like 6*		**RCOR1**	*REST corepressor 1*	
**LEPR**	*leptin receptor*		**CA12**	*carbonic anhydrase XII*	
**SDHC**	*succinate dehydrogenase complex, subunit C, integral membrane protein, 15kDa*		**ADD2**	*adducin 2 (beta)*	
**RPP14**	*ribonuclease P/MRP 14kDa subunit*		**BACE2**	*beta-site APP-cleaving enzyme 2*	
**YES1**	*v-yes-1 Yamaguchi sarcoma viral oncogene homolog 1*		**ROCK2**	*Rho-associated, coiled-coil containing protein kinase 2*	
**PCK1**	*phosphoenolpyruvate carboxykinase 1 (soluble)*		**RPL37A**	*ribosomal protein L37a*	
**AASDHPPT**	*aminoadipate-semialdehyde dehydrogenase-phosphopantetheinyl transferase*		**HSD17B12**	*hydroxysteroid (17-beta) dehydrogenase 12*	
**ACER3**	*alkaline ceramidase 3*		**USP49**	*ubiquitin specific peptidase 49*	
**RPL10**	*ribosomal protein L10*		**MECP2**	*methyl CpG binding protein 2 (Rett syndrome)*	
**ZNF181**	*zinc finger protein 181*		**SV2B**	*synaptic vesicle glycoprotein 2B*	
**CNTN6**	*contactin 6*		**THEM4**	*thioesterase superfamily member 4*	
**PPP1R3B**	*protein phosphatase 1, regulatory subunit 3B*		**G3BP1**	*GTPase activating protein (SH3 domain) binding protein 1*	
**ZNF587B**	*zinc finger protein 587B*		**ZNF576**	*zinc finger protein 576*	
**DNAH5**	*dynein, axonemal, heavy chain 5*		**TBC1D1**	*TBC1 (tre-2/USP6, BUB2, cdc16) domain family, member 1*	
**ZNF284**	*zinc finger protein 284*		**SAAL1**	*serum amyloid A-like 1*	
**CGGBP1**	*CGG triplet repeat binding protein 1*		**HOOK3**	*hook microtubule-tethering protein 3*	
**ULBP3**	*UL16 binding protein 3*		**ZRANB3**	*zinc finger, RAN-binding domain containing 3*	
**RAB6C**	*RAB6C, member RAS oncogene family*		**SNX1**	*sorting nexin 1*	
**KIFC2**	*kinesin family member C2*		**STRN3**	*striatin, calmodulin binding protein 3*	
**RECQL5**	*RecQ protein-like 5*		**PPP3CC**	*protein phosphatase 3, catalytic subunit, gamma isozyme*	
**MED9**	*mediator complex subunit 9*		**CDK13**	*cyclin-dependent kinase 13*	
**HOMER2**	*homer homolog 2 (Drosophila)*		**BMPR2**	*bone morphogenetic protein receptor, type II (serine/threonine kinase)*	

**FREM1**	*FRAS1 related extracellular matrix 1*		**TNFRSF11A**	*tumor necrosis factor receptor superfamily, member 11a, NFKB activator*	
**CAND1**	*cullin-associated and neddylation-dissociated 1*		**TPM3**	*tropomyosin 3*	
**EHHADH**	*enoyl-CoA, hydratase/3-hydroxyacyl CoA dehydrogenase*		**IBA57**	*IBA57, iron-sulfur cluster assembly homolog (S. cerevisiae)*	
**TMCO6**	*transmembrane and coiled-coil domains 6*		**SRI**	*sorcin*	
**ST8SIA3**	*ST8 alpha-N-acetyl-neuraminide alpha-2,8-sialyltransferase 3*		**PHF15**	*PHD finger protein 15*	
**PMEPA1**	*prostate transmembrane protein, androgen induced 1*		**STRAP**	*serine/threonine kinase receptor associated protein*	
**TAF1L**	*TAF1 RNA polymerase II, TATA box binding protein (TBP)-associated factor, 210kDa-like*		**DRAM2**	*DNA-damage regulated autophagy modulator 2*	
**TNPO1**	*transportin 1*		**RPL15**	*ribosomal protein L15*	
**CD151**	*CD151 molecule (Raph blood group)*		**CTSB**	*cathepsin B*	
**SERTM1**	*serine-rich and transmembrane domain containing 1*		**FADS6**	*fatty acid desaturase 6*	
**ZYG11B**	*zyg-11 family member B, cell cycle regulator*		**EDC3**	*enhancer of mRNA decapping 3*	
**FAM175B**	*family with sequence similarity 175, member B*		**DAB1**	*Dab, reelin signal transducer, homolog 1 (Drosophila)*	
**KLHL26**	*kelch-like family member 26*		**DENR**	*density-regulated protein*	
**CPA3**	*carboxypeptidase A3 (mast cell)*		**ZNF107**	*zinc finger protein 107*	
**SIDT1**	*SID1 transmembrane family, member 1*		**STARD5**	*StAR-related lipid transfer (START) domain containing 5*	
**RALBP1**	*ralA binding protein 1*		**ORMDL3**	*ORM1-like 3 (S. cerevisiae)*	
**TMEM87A**	*transmembrane protein 87A*		**FOXF1**	*forkhead box F1*	
**LPAR5**	*lysophosphatidic acid receptor 5*		**OSGIN2**	*oxidative stress induced growth inhibitor family member 2*	
**METTL5**	*methyltransferase like 5*		**NAA50**	*N(alpha)-acetyltransferase 50, NatE catalytic subunit*	
**ZC3H8**	*zinc finger CCCH-type containing 8*		**DOCK5**	*dedicator of cytokinesis 5*	
**USP8**	*ubiquitin specific peptidase 8*		**CXXC4**	*CXXC finger protein 4*	
**RB1**	*retinoblastoma 1*		**HERPUD2**	*HERPUD family member 2*	
**PTPN4**	*protein tyrosine phosphatase, non-receptor type 4 (megakaryocyte)*		**DNAJB4**	*DnaJ (Hsp40) homolog, subfamily B, member 4*	
**FGFR1OP2**	*FGFR1 oncogene partner 2*		**PLEKHA3**	*pleckstrin homology domain containing, family A (phosphoinositide binding specific) member 3*	

**RAB23**	*RAB23, member RAS oncogene family*		**HIF1AN**	*hypoxia inducible factor 1, alpha subunit inhibitor*	
**POU6F2**	*POU class 6 homeobox 2*		**ANP32B**	*acidic (leucine-rich) nuclear phosphoprotein 32 family, member B*	
**MAP3K5**	*mitogen-activated protein kinase kinase kinase 5*		**PERP**	*PERP, TP53 apoptosis effector*	
**PKD2**	*polycystic kidney disease 2 (autosomal dominant)*		**EMC2**	*ER membrane protein complex subunit 2*	
**TMEM78**	*transmembrane protein 78*		**IKZF3**	*IKAROS family zinc finger 3 (Aiolos)*	
**TUBGCP3**	*tubulin, gamma complex associated protein 3*		**FKBP5**	*FK506 binding protein 5*	
**CCDC132**	*coiled-coil domain containing 132*		**DNAJC21**	*DnaJ (Hsp40) homolog, subfamily C, member 21*	
**PIGO**	*phosphatidylinositol glycan anchor biosynthesis, class O*		**SIN3A**	*SIN3 transcription regulator family member A*	
**CCDC103**	*coiled-coil domain containing 103*		**PDS5A**	*PDS5, regulator of cohesion maintenance, homolog A (S. cerevisiae)*	
**RBM46**	*RNA binding motif protein 46*		**MRRF**	*mitochondrial ribosome recycling factor*	
**ZNF208**	*zinc finger protein 208*	✓	**CECR1**	*cat eye syndrome chromosome region, candidate 1*	
**LARP1**	*La ribonucleoprotein domain family, member 1*		**CNTN3**	*contactin 3 (plasmacytoma associated)*	
**SRSF10**	*serine/arginine-rich splicing factor 10*		**ABCG5**	*ATP-binding cassette, sub-family G (WHITE), member 5*	
**DTL**	*denticleless E3 ubiquitin protein ligase homolog (Drosophila)*		**ZNF417**	*zinc finger protein 417*	
**SLC39A10**	*solute carrier family 39 (zinc transporter), member 10*		**METTL10**	*methyltransferase like 10*	
**STRN**	*striatin, calmodulin binding protein*	✓	**SLFN5**	*schlafen family member 5*	
**TRAF6**	*TNF receptor-associated factor 6, E3 ubiquitin protein ligase*		**KCTD16**	*potassium channel tetramerization domain containing 16*	
**CCDC117**	*coiled-coil domain containing 117*		**TNFRSF10A**	*tumor necrosis factor receptor superfamily, member 10a*	
**PDE10A**	*phosphodiesterase 10A*		**RGL2**	*ral guanine nucleotide dissociation stimulator-like 2*	
**AAR2**	*AAR2 splicing factor homolog (S. cerevisiae)*		**PEX26**	*peroxisomal biogenesis factor 26*	
**BACH1**	*BTB and CNC homology 1, basic leucine zipper transcription factor 1*		**BCAS4**	*breast carcinoma amplified sequence 4*	
**ZNF99**	*zinc finger protein 99*		**COX15**	*cytochrome c oxidase assembly homolog 15 (yeast)*	
**CXCL5**	*chemokine (C-X-C motif) ligand 5*		**NSUN4**	*NOP2/Sun domain family, member 4*	
**GDNF**	*glial cell derived neurotrophic factor*		**WSB1**	*WD repeat and SOCS box containing 1*	

**EXPH5**	*exophilin 5*		**CCDC174**	*coiled-coil domain containing 174*	
**PIGM**	*phosphatidylinositol glycan anchor biosynthesis, class M*		**WDR12**	*WD repeat domain 12*	
**NUDT5**	*nudix (nucleoside diphosphate linked moiety X)-type motif 5*		**KIN**	*KIN, antigenic determinant of recA protein homolog (mouse)*	
**FAM154A**	*family with sequence similarity 154, member A*		**RSBN1L**	*round spermatid basic protein 1-like*	
**OCRL**	*oculocerebrorenal syndrome of Lowe*		**ARL5A**	*ADP-ribosylation factor-like 5A*	
**ERVMER34-1**	*endogenous retrovirus group MER34, member 1*		**PGBD1**	*piggyBac transposable element derived 1*	
**CCDC28A**	*coiled-coil domain containing 28A*		**STAMBPL1**	*STAM binding protein-like 1*	
**RASEF**	*RAS and EF-hand domain containing*		**MSANTD3**	*Myb/SANT-like DNA-binding domain containing 3*	
**SAMD10**	*sterile alpha motif domain containing 10*		**RNPEPL1**	*arginyl aminopeptidase (aminopeptidase B)-like 1*	
**TAB2**	*TGF-beta activated kinase 1/MAP3K7 binding protein 2*		**FUT4**	*fucosyltransferase 4 (alpha (1,3) fucosyltransferase, myeloid-specific)*	
**CCND1**	*cyclin D1*		**COBLL1**	*cordon-bleu WH2 repeat protein-like 1*	
**COL6A1**	*collagen, type VI, alpha 1*		**NBN**	*nibrin*	
**RORA**	*RAR-related orphan receptor A*		**ACSS3**	*acyl-CoA synthetase short-chain family member 3*	
**HAUS6**	*HAUS augmin-like complex, subunit 6*		**ANTXR2**	*anthrax toxin receptor 2*	
**LPHN3**	*latrophilin 3*		**LDLRAD4**	*low density lipoprotein receptor class A domain containing 4*	
**RAB9B**	*RAB9B, member RAS oncogene family*		**SLC2A8**	*solute carrier family 2 (facilitated glucose transporter), member 8*	
**SC5D**	*sterol-C5-desaturase*		**ANKHD1-EIF4EBP3**	*ANKHD1-EIF4EBP3 readthrough*	
**FUNDC2**	*FUN14 domain containing 2*		**HSBP1**	*heat shock factor binding protein 1*	
**PARP8**	*poly (ADP-ribose) polymerase family, member 8*		**BPTF**	*bromodomain PHD finger transcription factor*	
**SLC37A3**	*solute carrier family 37, member 3*		**ING2**	*inhibitor of growth family, member 2*	
**PUM1**	*pumilio RNA-binding family member 1*		**TGS1**	*trimethylguanosine synthase 1*	
**GIT2**	*G protein-coupled receptor kinase interacting ArfGAP 2*		**PAX6**	*paired box 6*	
**CEP41**	*centrosomal protein 41kDa*		**XRRA1**	*X-ray radiation resistance associated 1*	
**SOCS6**	*suppressor of cytokine signaling 6*		**PHKA2**	*phosphorylase kinase, alpha 2 (liver)*	

**CCSAP**	*centriole, cilia and spindle-associated protein*		**ZNF860**	*zinc finger protein 860*	
**PDE8A**	*phosphodiesterase 8A*		**PELP1**	*proline, glutamate and leucine rich protein 1*	
**CHP2**	*calcineurin-like EF-hand protein 2*		**GALNT5**	*UDP-N-acetyl-alpha-D-galactosamine:polypeptide N-acetylgalactosaminyltransferase 5 (GalNAc-T5)*	
**PTAR1**	*protein prenyltransferase alpha subunit repeat containing 1*		**NABP1**	*nucleic acid binding protein 1*	
**RNF128**	*ring finger protein 128, E3 ubiquitin protein ligase*		**DDX20**	*DEAD (Asp-Glu-Ala-Asp) box polypeptide 20*	
**HSD11B1**	*hydroxysteroid (11-beta) dehydrogenase 1*		**TMEM106B**	*transmembrane protein 106B*	
**LMO3**	*LIM domain only 3 (rhombotin-like 2)*		**LEPROTL1**	*leptin receptor overlapping transcript-like 1*	
**ZNF180**	*zinc finger protein 180*		**FAM104B**	*family with sequence similarity 104, member B*	
**GJA3**	*gap junction protein, alpha 3, 46kDa*		**TIMP4**	*TIMP metallopeptidase inhibitor 4*	
**FUBP1**	*far upstream element (FUSE) binding protein 1*		**CEP72**	*centrosomal protein 72kDa*	
**ZNF546**	*zinc finger protein 546*		**NDUFS1**	*NADH dehydrogenase (ubiquinone) Fe-S protein 1, 75kDa (NADH-coenzyme Q reductase)*	
**PRKAA1**	*protein kinase, AMP-activated, alpha 1 catalytic subunit*		**NSUN3**	*NOP2/Sun domain family, member 3*	
**KLHL29**	*kelch-like family member 29*		**TTC37**	*tetratricopeptide repeat domain 37*	
**MYF5**	*myogenic factor 5*		**MAPRE2**	*microtubule-associated protein, RP/EB family, member 2*	
**ZNF512B**	*zinc finger protein 512B*	✓	**CHML**	*choroideremia-like (Rab escort protein 2)*	
**SLC7A14**	*solute carrier family 7, member 14*		**NAT10**	*N-acetyltransferase 10 (GCN5-related)*	
**RAB27A**	*RAB27A, member RAS oncogene family*		**SPA17**	*sperm autoantigenic protein 17*	
**GXYLT1**	*glucoside xylosyltransferase 1*		**MRPS14**	*mitochondrial ribosomal protein S14*	
**RSPRY1**	*ring finger and SPRY domain containing 1*		**LPHN2**	*latrophilin 2*	
**CLEC2D**	*C-type lectin domain family 2, member D*		**MED20**	*mediator complex subunit 20*	
**PLEKHA8**	*pleckstrin homology domain containing, family A (phosphoinositide binding specific) member 8*		**ERCC6L**	*excision repair cross-complementing rodent repair deficiency, complementation group 6-like*	
**LPAR1**	*lysophosphatidic acid receptor 1*		**SSBP2**	*single-stranded DNA binding protein 2*	
**ZNF318**	*zinc finger protein 318*		**CLYBL**	*citrate lyase beta like*	
**CLDND1**	*claudin domain containing 1*	✓	**GANAB**	*glucosidase, alpha; neutral AB*	

**ALG10B**	*ALG10B, alpha-1,2-glucosyltransferase*		**SFPQ**	*splicing factor proline/glutamine-rich*	
**CDC42EP3**	*CDC42 effector protein (Rho GTPase binding) 3*		**TRIM45**	*tripartite motif containing 45*	
**RP11-1220K2.2**	*Putative inactive maltase-glucoamylase-like protein LOC93432*		**HECW2**	*HECT, C2 and WW domain containing E3 ubiquitin protein ligase 2*	
**SLC5A2**	*solute carrier family 5 (sodium/glucose cotransporter), member 2*		**MKKS**	*McKusick-Kaufman syndrome*	
**SNN**	*stannin*		**MRPL48**	*mitochondrial ribosomal protein L48*	
**NLRP3**	*NLR family, pyrin domain containing 3*		**SETD9**	*SET domain containing 9*	
**OPCML**	*opioid binding protein/cell adhesion molecule-like*		**MGAT4A**	*mannosyl (alpha-1,3-)-glycoprotein beta-1,4-N-acetylglucosaminyltransferase, isozyme A*	
**GPRIN3**	*GPRIN family member 3*		**MCF2L**	*MCF.2 cell line derived transforming sequence-like*	
**AIMP1**	*aminoacyl tRNA synthetase complex-interacting multifunctional protein 1*		**FUT10**	*fucosyltransferase 10 (alpha (1,3) fucosyltransferase)*	
**FAM105B**	*family with sequence similarity 105, member B*		**MESP1**	*mesoderm posterior 1 homolog (mouse)*	
**TMEM117**	*transmembrane protein 117*		**TMEFF2**	*transmembrane protein with EGF-like and two follistatin-like domains 2*	
**ANKRD20A3**	*ankyrin repeat domain 20 family, member A3*		**TCEA3**	*transcription elongation factor A (SII), 3*	
**HSPA5**	*heat shock 70kDa protein 5 (glucose-regulated protein, 78kDa)*	✓	**GABPB1**	*GA binding protein transcription factor, beta subunit 1*	
**ASH2L**	*ash2 (absent, small, or homeotic)-like (Drosophila)*		**TAF1D**	*TATA box binding protein (TBP)-associated factor, RNA polymerase I, D, 41kDa*	
**KLHL1**	*kelch-like family member 1*		**KLHL7**	*kelch-like family member 7*	
**TRPM3**	*transient receptor potential cation channel, subfamily M, member 3*		**VPS4A**	*vacuolar protein sorting 4 homolog A (S. cerevisiae)*	
**B3GALTL**	*beta 1,3-galactosyltransferase-like*		**SELK**	*Selenoprotein K*	
**ICK**	*intestinal cell (MAK-like) kinase*		**FAM179A**	*family with sequence similarity 179, member A*	
**ZIC3**	*Zic family member 3*		**MND1**	*meiotic nuclear divisions 1 homolog (S. cerevisiae)*	
**AL033381.1**	*Uncharacterized protein; cDNA FLJ34594 fis, clone KIDNE2009109*		**SENP1**	*SUMO1/sentrin specific peptidase 1*	
**ORAI2**	*ORAI calcium release-activated calcium modulator 2*		**H2AFX**	*H2A histone family, member X*	
**ANKRD20A4**	*ankyrin repeat domain 20 family, member A4*		**TMEM194B**	*transmembrane protein 194B*	
**PHB**	*prohibitin*		**PCNP**	*PEST proteolytic signal containing nuclear protein*	

**ZC3H6**	*zinc finger CCCH-type containing 6*		**SLITRK4**	*SLIT and NTRK-like family, member 4*	
**MTMR9**	*myotubularin related protein 9*		**NEDD4L**	*neural precursor cell expressed, developmentally down-regulated 4-like, E3 ubiquitin protein ligase*	
**MIEF1**	*mitochondrial elongation factor 1*		**ZNF326**	*zinc finger protein 326*	
**PDE3A**	*phosphodiesterase 3A, cGMP-inhibited*		**GTPBP8**	*GTP-binding protein 8 (putative)*	
**LRRD1**	*leucine-rich repeats and death domain containing 1*		**ETV6**	*ets variant 6*	
**FAM129A**	*family with sequence similarity 129, member A*		**SERINC1**	*serine incorporator 1*	
**ANKS1B**	*ankyrin repeat and sterile alpha motif domain containing 1B*		**EIF2B5**	*eukaryotic translation initiation factor 2B, subunit 5 epsilon, 82kDa*	
**GPR17**	*G protein-coupled receptor 17*		**NAP1L1**	*nucleosome assembly protein 1-like 1*	
**PEX5**	*peroxisomal biogenesis factor 5*		**ZNF445**	*zinc finger protein 445*	
**ZEB1**	*zinc finger E-box binding homeobox 1*		**LHFPL4**	*lipoma HMGIC fusion partner-like 4*	
**MST4**	*Serine/threonine-protein kinase MST4*		**RBM33**	*RNA binding motif protein 33*	
**GUF1**	*GUF1 GTPase homolog (S. cerevisiae)*		**FOXK2**	*forkhead box K2*	
**PCYOX1**	*prenylcysteine oxidase 1*		**DDR2**	*discoidin domain receptor tyrosine kinase 2*	
**AGFG1**	*ArfGAP with FG repeats 1*		**GSTM3**	*glutathione S-transferase mu 3 (brain)*	
**PDLIM5**	*PDZ and LIM domain 5*		**VANGL1**	*VANGL planar cell polarity protein 1*	
**ANP32E**	*acidic (leucine-rich) nuclear phosphoprotein 32 family, member E*		**ERCC8**	*excision repair cross-complementing rodent repair deficiency, complementation group 8*	
**CCDC68**	*coiled-coil domain containing 68*		**SRFBP1**	*serum response factor binding protein 1*	
**CTD-3074O7.11**	*Bardet-Biedl syndrome 1 protein*		**VPS18**	*vacuolar protein sorting 18 homolog (S. cerevisiae)*	
**CRISPLD1**	*cysteine-rich secretory protein LCCL domain containing 1*		**LIFR**	*leukemia inhibitory factor receptor alpha*	
**KLHL13**	*kelch-like family member 13*		**C1orf21**	*chromosome 1 open reading frame 21*	
**EXOC8**	*exocyst complex component 8*		**ANKRD13C**	*ankyrin repeat domain 13C*	
**ENPP5**	*ectonucleotide pyrophosphatase/phosphodiesterase 5 (putative)*		**DNTTIP2**	*deoxynucleotidyltransferase, terminal, interacting protein 2*	✓
**GPALPP1**	*GPALPP motifs containing 1*		**WDR3**	*WD repeat domain 3*	
**PDHA1**	*pyruvate dehydrogenase (lipoamide) alpha 1*		**CSE1L**	*CSE1 chromosome segregation 1-like (yeast)*	

**TLDC2**	*TBC/LysM-associated domain containing 2*		**GPR133**	*G protein-coupled receptor 133*	
**PCP4L1**	*Purkinje cell protein 4 like 1*		**CENPI**	*centromere protein I*	
**PPP1R3G**	*protein phosphatase 1, regulatory subunit 3G*		**C9orf64**	*chromosome 9 open reading frame 64*	
**PDE8B**	*phosphodiesterase 8B*		**TMCO1**	*transmembrane and coiled-coil domains 1*	
**MANEAL**	*mannosidase, endo-alpha-like*		**IWS1**	*IWS1 homolog (S. cerevisiae)*	
**SLCO4C1**	*solute carrier organic anion transporter family, member 4C1*		**PTGS1**	*prostaglandin-endoperoxide synthase 1 (prostaglandin G/H synthase and cyclooxygenase)*	
**DHX36**	*DEAH (Asp-Glu-Ala-His) box polypeptide 36*		**ANKFY1**	*ankyrin repeat and FYVE domain containing 1*	
**ZNF556**	*zinc finger protein 556*		**GNB2L1**	*guanine nucleotide binding protein (G protein), beta polypeptide 2-like 1*	
**CLMP**	*CXADR-like membrane protein*		**HPX**	*hemopexin*	
**ZNF300**	*zinc finger protein 300*		**SCN9A**	*sodium channel, voltage-gated, type IX, alpha subunit*	
**SELL**	*selectin L*		**GINM1**	*glycoprotein integral membrane 1*	
**HOXC8**	*homeobox C8*		**KB-1507C5.2**	*HCG15011, isoform CRA_a; Protein LOC100996457*	
**C17orf96**	*chromosome 17 open reading frame 96*		**THPO**	*thrombopoietin*	
**RIMBP3B**	*RIMS binding protein 3B*		**C1orf27**	*chromosome 1 open reading frame 27*	
**CSNK1G1**	*casein kinase 1, gamma 1*		**DR1**	*down-regulator of transcription 1, TBP-binding (negative cofactor 2)*	
**RIMBP3**	*RIMS binding protein 3*		**PCDHB5**	*protocadherin beta 5*	
**CRK**	*v-crk avian sarcoma virus CT10 oncogene homolog*		**RNF111**	*ring finger protein 111*	
**PKN2**	*protein kinase N2*		**NFE2L3**	*nuclear factor, erythroid 2-like 3*	
**TRIM25**	*tripartite motif containing 25*		**UQCRFS1**	*ubiquinol-cytochrome c reductase, Rieske iron-sulfur polypeptide 1*	
**PATZ1**	*POZ (BTB) and AT hook containing zinc finger 1*		**ECHDC2**	*enoyl CoA hydratase domain containing 2*	
**ZDHHC3**	*zinc finger, DHHC-type containing 3*		**SERPINA4**	*serpin peptidase inhibitor, clade A (alpha-1 antiproteinase, antitrypsin), member 4*	
**DCBLD2**	*discoidin, CUB and LCCL domain containing 2*		**CLMN**	*calmin (calponin-like, transmembrane)*	
**HBP1**	*HMG-box transcription factor 1*		**CTSS**	*cathepsin S*	
**FMNL2**	*formin-like 2*		**VEZT**	*vezatin, adherens junctions transmembrane protein*	

**KANSL1L**	*KAT8 regulatory NSL complex subunit 1-like*		**FBXO9**	*F-box protein 9*	
**FAM86C1**	*family with sequence similarity 86, member C1*		**DHX34**	*DEAH (Asp-Glu-Ala-His) box polypeptide 34*	
**ANXA4**	*annexin A4*		**REPS2**	*RALBP1 associated Eps domain containing 2*	
**GNG11**	*guanine nucleotide binding protein (G protein), gamma 11*		**FAM134A**	*family with sequence similarity 134, member A*	
**WDR89**	*WD repeat domain 89*		**TOMM6**	*translocase of outer mitochondrial membrane 6 homolog (yeast)*	
**SDAD1**	*SDA1 domain containing 1*		**ABCF3**	*ATP-binding cassette, sub-family F (GCN20), member 3*	
**SRSF1**	*serine/arginine-rich splicing factor 1*		**STOML2**	*stomatin (EPB72)-like 2*	
**ARL6IP5**	*ADP-ribosylation-like factor 6 interacting protein 5*		**RBP4**	*retinol binding protein 4, plasma*	
**GNA13**	*guanine nucleotide binding protein (G protein), alpha 13*		**UCHL1**	*ubiquitin carboxyl-terminal esterase L1 (ubiquitin thiolesterase)*	
**BBS1**	*Bardet-Biedl syndrome 1*		**C2orf72**	*chromosome 2 open reading frame 72*	
**RP11-644F5.10**	*Uncharacterized protein*		**IPO9**	*importin 9*	
**TXNDC2**	*thioredoxin domain containing 2 (spermatozoa)*		**MRTO4**	*mRNA turnover 4 homolog (S. cerevisiae)*	
**ZNF333**	*zinc finger protein 333*		**SFXN2**	*sideroflexin 2*	
**TMEM182**	*transmembrane protein 182*		**UQCR10**	*ubiquinol-cytochrome c reductase, complex III subunit X*	
**SLC16A4**	*solute carrier family 16, member 4*		**SOCS2**	*suppressor of cytokine signaling 2*	
**ZNF224**	*zinc finger protein 224*		**C15orf61**	*chromosome 15 open reading frame 61*	
**P2RX1**	*purinergic receptor P2X, ligand-gated ion channel, 1*		**WDFY2**	*WD repeat and FYVE domain containing 2*	
**TRIM14**	*tripartite motif containing 14*		**GLUL**	*glutamate-ammonia ligase*	
**ZNF264**	*zinc finger protein 264*		**ATP5G3**	*ATP synthase, H+ transporting, mitochondrial Fo complex, subunit C3 (subunit 9)*	
**CCBE1**	*collagen and calcium binding EGF domains 1*		**BRMS1L**	*breast cancer metastasis-suppressor 1-like*	
**KIF27**	*kinesin family member 27*		**PAICS**	*phosphoribosylaminoimidazole carboxylase, phosphoribosylaminoimidazole succinocarboxamide synthetase*	
**ZBTB21**	*zinc finger and BTB domain containing 21*		**ZFC3H1**	*zinc finger, C3H1-type containing*	
**ZXDC**	*ZXD family zinc finger C*		**SLC35D2**	*solute carrier family 35 (UDP-GlcNAc/UDP-glucose transporter), member D2*	

**CD300C**	*CD300c molecule*		**SMC1A**	*structural maintenance of chromosomes 1A*	
**NCOA7**	*nuclear receptor coactivator 7*		**C1orf112**	*chromosome 1 open reading frame 112*	
**RDX**	*radixin*		**ZNF607**	*zinc finger protein 607*	
**CARD11**	*caspase recruitment domain family, member 11*		**HIF1A**	*hypoxia inducible factor 1, alpha subunit (basic helix-loop-helix transcription factor)*	
**LHFPL3**	*lipoma HMGIC fusion partner-like 3*		**NCKAP1**	*NCK-associated protein 1*	
**PAG1**	*phosphoprotein associated with glycosphingolipid microdomains 1*		**IGSF3**	*immunoglobulin superfamily, member 3*	
**COX10**	*cytochrome c oxidase assembly homolog 10 (yeast)*		**GDAP2**	*ganglioside induced differentiation associated protein 2*	
**RABIF**	*RAB interacting factor*		**STRIP2**	*striatin interacting protein 2*	
**FKRP**	*fukutin related protein*		**LIPC**	*lipase, hepatic*	
**DHX33**	*DEAH (Asp-Glu-Ala-His) box polypeptide 33*		**NTPCR**	*nucleoside-triphosphatase, cancer-related*	
**UEVLD**	*UEV and lactate/malate dehyrogenase domains*		**ANXA5**	*annexin A5*	
**CCDC41**	*coiled-coil domain containing 41*		**CNOT7**	*CCR4-NOT transcription complex, subunit 7*	
**TRPV3**	*transient receptor potential cation channel, subfamily V, member 3*		**COPS2**	*COP9 signalosome subunit 2*	
**UBA6**	*ubiquitin-like modifier activating enzyme 6*		**LCORL**	*ligand dependent nuclear receptor corepressor-like*	
**SEC62**	*SEC62 homolog (S. cerevisiae)*		**IAH1**	*isoamyl acetate-hydrolyzing esterase 1 homolog (S. cerevisiae)*	
**TMEM260**	*transmembrane protein 260*		**ABI2**	*abl-interactor 2*	
**FER**	*fer (fps/fes related) tyrosine kinase*		**PPIP5K2**	*diphosphoinositol pentakisphosphate kinase 2*	
**ELAVL2**	*ELAV like neuron-specific RNA binding protein 2*		**SRSF6**	*serine/arginine-rich splicing factor 6*	
**BICD1**	*bicaudal D homolog 1 (Drosophila)*		**APEX2**	*APEX nuclease (apurinic/apyrimidinic endonuclease) 2*	
**KIAA1024L**	*KIAA1024-like*		**F10**	*coagulation factor X*	
**MAP2K3**	*mitogen-activated protein kinase kinase 3*		**CDH2**	*cadherin 2, type 1, N-cadherin (neuronal)*	
**ESCO1**	*establishment of sister chromatid cohesion N-acetyltransferase 1*		**DSN1**	*DSN1, MIS12 kinetochore complex component*	
**CERS6**	*ceramide synthase 6*		**RPAP2**	*RNA polymerase II associated protein 2*	
**PCDH7**	*protocadherin 7*		**CYB5R4**	*cytochrome b5 reductase 4*	

**CST3**	*cystatin C*		**RAB18**	*RAB18, member RAS oncogene family*	
**B3GAT2**	*beta-1,3-glucuronyltransferase 2 (glucuronosyltransferase S)*		**GTDC1**	*glycosyltransferase-like domain containing 1*	
**KIF13B**	*kinesin family member 13B*		**AMACR**	*alpha-methylacyl-CoA racemase*	
**FAM86A**	*family with sequence similarity 86, member A*		**CCDC84**	*coiled-coil domain containing 84*	
**FDFT1**	*farnesyl-diphosphate farnesyltransferase 1*		**PITPNC1**	*phosphatidylinositol transfer protein, cytoplasmic 1*	
**STARD13**	*StAR-related lipid transfer (START) domain containing 13*		**KIAA1462**	*KIAA1462*	
**ZNF709**	*zinc finger protein 709*		**RNF8**	*ring finger protein 8, E3 ubiquitin protein ligase*	
**FLT1**	*fms-related tyrosine kinase 1*		**MLF1**	*myeloid leukemia factor 1*	
**FNDC3B**	*fibronectin type III domain containing 3B*		**GNB4**	*guanine nucleotide binding protein (G protein), beta polypeptide 4*	
**COL8A2**	*collagen, type VIII, alpha 2*		**RPP30**	*ribonuclease P/MRP 30kDa subunit*	
**MEI4**	*meiosis-specific 4 homolog (S. cerevisiae)*		**ARHGAP29**	*Rho GTPase activating protein 29*	
**EID2B**	*EP300 interacting inhibitor of differentiation 2B*		**PDS5B**	*PDS5, regulator of cohesion maintenance, homolog B (S. cerevisiae)*	
**BOD1L2**	*biorientation of chromosomes in cell division 1-like 2*		**PDE4D**	*phosphodiesterase 4D, cAMP-specific*	
**ZNF629**	*zinc finger protein 629*		**NUPL2**	*nucleoporin like 2*	
**SNX10**	*sorting nexin 10*		**ENAH**	*enabled homolog (Drosophila)*	
**ZNF350**	*zinc finger protein 350*		**ITGB5**	*integrin, beta 5*	
**HDX**	*highly divergent homeobox*		**BRD9**	*bromodomain containing 9*	
**TMC7**	*transmembrane channel-like 7*		**SOCS7**	*suppressor of cytokine signaling 7*	
**FUT2**	*fucosyltransferase 2 (secretor status included)*		**HNRNPR**	*heterogeneous nuclear ribonucleoprotein R*	
**STXBP5L**	*syntaxin binding protein 5-like*		**BDNF**	*brain-derived neurotrophic factor*	
**ZNF304**	*zinc finger protein 304*		**ZFP14**	*ZFP14 zinc finger protein*	
**STK32A**	*serine/threonine kinase 32A*		**CMC1**	*COX assembly mitochondrial protein 1 homolog (S. cerevisiae)*	
**ATXN3**	*ataxin 3*		**ALG14**	*ALG14, UDP-N-acetylglucosaminyltransferase subunit*	
**TAL2**	*T-cell acute lymphocytic leukemia 2*		**FXR1**	*fragile X mental retardation, autosomal homolog 1*	

**MDFIC**	*MyoD family inhibitor domain containing*		**ADRBK2**	*adrenergic, beta, receptor kinase 2*	
**INPP4B**	*inositol polyphosphate-4-phosphatase, type II, 105kDa*		**C1orf50**	*chromosome 1 open reading frame 50*	
**PRPF38A**	*pre-mRNA processing factor 38A*		**TSPAN14**	*tetraspanin 14*	
**TBR1**	*T-box, brain, 1*		**SIX1**	*SIX homeobox 1*	
**ARHGAP19-SLIT1**	*ARHGAP19-SLIT1 readthrough (NMD candidate)*		**RBM25**	*RNA binding motif protein 25*	
**CIR1**	*corepressor interacting with RBPJ, 1*		**GBX2**	*gastrulation brain homeobox 2*	
**KLRC3**	*killer cell lectin-like receptor subfamily C, member 3*		**SGIP1**	*SH3-domain GRB2-like (endophilin) interacting protein 1*	
**CYCS**	*cytochrome c, somatic*		**TRDMT1**	*tRNA aspartic acid methyltransferase 1*	
**BCLAF1**	*BCL2-associated transcription factor 1*		**YIPF4**	*Yip1 domain family, member 4*	
**PIK3AP1**	*phosphoinositide-3-kinase adaptor protein 1*		**AKR7A2**	*aldo-keto reductase family 7, member A2 (aflatoxin aldehyde reductase)*	
**BIVM**	*basic, immunoglobulin-like variable motif containing*		**STX3**	*syntaxin 3*	
**SAP130**	*Sin3A-associated protein, 130kDa*		**DDHD2**	*DDHD domain containing 2*	
**EEA1**	*early endosome antigen 1*		**RBM15B**	*RNA binding motif protein 15B*	
**STOX2**	*storkhead box 2*		**XPNPEP3**	*X-prolyl aminopeptidase (aminopeptidase P) 3, putative*	
**GPR126**	*G protein-coupled receptor 126*		**ZCCHC9**	*zinc finger, CCHC domain containing 9*	
**FAM98B**	*family with sequence similarity 98, member B*		**RMND5A**	*required for meiotic nuclear division 5 homolog A (S. cerevisiae)*	
**FNIP2**	*folliculin interacting protein 2*		**PPIF**	*peptidylprolyl isomerase F*	
**EVX2**	*even-skipped homeobox 2*		**PCSK9**	*proprotein convertase subtilisin/kexin type 9*	
**SHPRH**	*SNF2 histone linker PHD RING helicase, E3 ubiquitin protein ligase*		**ZC3H14**	*zinc finger CCCH-type containing 14*	
**NEDD9**	*neural precursor cell expressed, developmentally down-regulated 9*		**ATP2B2**	*ATPase, Ca++ transporting, plasma membrane 2*	
**PNN**	*pinin, desmosome associated protein*		**SNX2**	*sorting nexin 2*	
**GABRA2**	*gamma-aminobutyric acid (GABA) A receptor, alpha 2*		**MAPK14**	*mitogen-activated protein kinase 14*	
**WDR44**	*WD repeat domain 44*		**LTBP2**	*latent transforming growth factor beta binding protein 2*	
**ZNF596**	*zinc finger protein 596*		**ARL8B**	*ADP-ribosylation factor-like 8B*	

**ZNF704**	*zinc finger protein 704*		**MSRB2**	*methionine sulfoxide reductase B2*	
**GPR179**	*G protein-coupled receptor 179*		**ZNF211**	*zinc finger protein 211*	
**POU3F2**	*POU class 3 homeobox 2*		**RNASEH2C**	*ribonuclease H2, subunit C*	
**BMP2K**	*BMP2 inducible kinase*		**VEGFC**	*vascular endothelial growth factor C*	
**EPS8**	*epidermal growth factor receptor pathway substrate 8*		**ZNF747**	*zinc finger protein 747*	
**BRCA1**	*breast cancer 1, early onset*		**ECHDC3**	*enoyl CoA hydratase domain containing 3*	
**ATF7IP**	*activating transcription factor 7 interacting protein*		**TUBD1**	*tubulin, delta 1*	
**KIF20A**	*kinesin family member 20A*		**RASSF4**	*Ras association (RalGDS/AF-6) domain family member 4*	
**SNTB2**	*syntrophin, beta 2 (dystrophin-associated protein A1, 59kDa, basic component 2)*		**CYP4F12**	*cytochrome P450, family 4, subfamily F, polypeptide 12*	
**PAQR5**	*progestin and adipoQ receptor family member V*		**COLEC10**	*collectin sub-family member 10 (C-type lectin)*	
**IRGQ**	*immunity-related GTPase family, Q*		**EPB41L1**	*erythrocyte membrane protein band 4.1-like 1*	
**ZSCAN22**	*zinc finger and SCAN domain containing 22*		**BAG1**	*BCL2-associated athanogene*	
**LEP**	*leptin*		**VIL1**	*villin 1*	
**PRPF4B**	*pre-mRNA processing factor 4B*		**GATA6**	*GATA binding protein 6*	
**KCND3**	*potassium voltage-gated channel, Shal-related subfamily, member 3*		**RFFL**	*ring finger and FYVE-like domain containing E3 ubiquitin protein ligase*	
**WAC**	*WW domain containing adaptor with coiled-coil*		**SF3A1**	*splicing factor 3a, subunit 1, 120kDa*	
**SERPINB9**	*serpin peptidase inhibitor, clade B (ovalbumin), member 9*		**SLBP**	*stem-loop binding protein*	
**HS3ST3B1**	*heparan sulfate (glucosamine) 3-O-sulfotransferase 3B1*		**CABP4**	*calcium binding protein 4*	
**PGM2L1**	*phosphoglucomutase 2-like 1*		**FTO**	*fat mass and obesity associated*	
**DDX21**	*DEAD (Asp-Glu-Ala-Asp) box helicase 21*		**DEFB107A**	*defensin, beta 107A*	
**STXBP4**	*syntaxin binding protein 4*		**HAS2**	*hyaluronan synthase 2*	
**KLRC4**	*killer cell lectin-like receptor subfamily C, member 4*		**ANKRD32**	*ankyrin repeat domain 32*	
**ZNF730**	*zinc finger protein 730*		**NEK10**	*NIMA-related kinase 10*	
**TPH2**	*tryptophan hydroxylase 2*		**POTED**	*POTE ankyrin domain family, member D*	

**REXO1L1**	*REX1, RNA exonuclease 1 homolog (S. cerevisiae)-like 1*		**DEFB107B**	*defensin, beta 107B*	
**FAM178A**	*family with sequence similarity 178, member A*		**ODF2L**	*outer dense fiber of sperm tails 2-like*	
**MTRF1**	*mitochondrial translational release factor 1*		**ZNF281**	*zinc finger protein 281*	
**OSBPL6**	*oxysterol binding protein-like 6*		**FHL2**	*four and a half LIM domains 2*	
**FBXO45**	*F-box protein 45*		**TRPM7**	*transient receptor potential cation channel, subfamily M, member 7*	
**ASXL3**	*additional sex combs like 3 (Drosophila)*		**EGF**	*epidermal growth factor*	
**GRIP1**	*glutamate receptor interacting protein 1*		**GNB1L**	*guanine nucleotide binding protein (G protein), beta polypeptide 1-like*	
**SYNPR**	*synaptoporin*		**SLFN12L**	*schlafen family member 12-like*	
**IMPAD1**	*inositol monophosphatase domain containing 1*		**AKR1C2**	*aldo-keto reductase family 1, member C2*	
**FAM111B**	*family with sequence similarity 111, member B*		**FSTL1**	*follistatin-like 1*	
**FOXL2**	*forkhead box L2*		**CAPN2**	*calpain 2, (m/II) large subunit*	
**DENND4C**	*DENN/MADD domain containing 4C*		**WDR33**	*WD repeat domain 33*	
**METTL15**	*methyltransferase like 15*		**RARS2**	*arginyl-tRNA synthetase 2, mitochondrial*	
**PSG4**	*pregnancy specific beta-1-glycoprotein 4*		**AGTRAP**	*angiotensin II receptor-associated protein*	
**BCAS2**	*breast carcinoma amplified sequence 2*		**TTPA**	*tocopherol (alpha) transfer protein*	
**OTUD3**	*OTU domain containing 3*		**MORC1**	*MORC family CW-type zinc finger 1*	
**MYO5C**	*myosin VC*		**PLRG1**	*pleiotropic regulator 1*	
**ALDH5A1**	*aldehyde dehydrogenase 5 family, member A1*		**CPS1**	*carbamoyl-phosphate synthase 1, mitochondrial*	
**FBXO30**	*F-box protein 30*		**PPWD1**	*peptidylprolyl isomerase domain and WD repeat containing 1*	
**RNF114**	*ring finger protein 114*		**LETMD1**	*LETM1 domain containing 1*	
**C21orf62**	*chromosome 21 open reading frame 62*		**CYSLTR2**	*cysteinyl leukotriene receptor 2*	
**PRSS12**	*protease, serine, 12 (neurotrypsin, motopsin)*		**CASK**	*calcium/calmodulin-dependent serine protein kinase (MAGUK family)*	
**THAP11**	*THAP domain containing 11*		**COG5**	*component of oligomeric golgi complex 5*	
**PCLO**	*piccolo presynaptic cytomatrix protein*		**GPR113**	*G protein-coupled receptor 113*	

**BX255923.1**	*HDCMB45P; Uncharacterized protein*		**BTN3A2**	*butyrophilin, subfamily 3, member A2*	
**DACH1**	*dachshund homolog 1 (Drosophila)*		**CDR1**	*cerebellar degeneration-related protein 1, 34kDa*	
**SLC30A9**	*solute carrier family 30 (zinc transporter), member 9*		**HDDC2**	*HD domain containing 2*	
**CCNB1IP1**	*cyclin B1 interacting protein 1, E3 ubiquitin protein ligase*		**BRIX1**	*BRX1, biogenesis of ribosomes, homolog (S. cerevisiae)*	
**KLHL8**	*kelch-like family member 8*		**FAM162B**	*family with sequence similarity 162, member B*	
**PVR**	*poliovirus receptor*		**EMC7**	*ER membrane protein complex subunit 7*	
**APPL1**	*adaptor protein, phosphotyrosine interaction, PH domain and leucine zipper containing 1*		**PCDH11X**	*protocadherin 11 X-linked*	
**ZFP1**	*ZFP1 zinc finger protein*		**BTN3A1**	*butyrophilin, subfamily 3, member A1*	
**AL117190.3**	*Esophagus cancer-related gene-2 interaction susceptibility protein; Uncharacterized protein*		**BOD1**	*biorientation of chromosomes in cell division 1*	
**USP6NL**	*USP6 N-terminal like*	✓	**ALG13**	*ALG13, UDP-N-acetylglucosaminyltransferase subunit*	
**TNFRSF21**	*tumor necrosis factor receptor superfamily, member 21*	✓	**CCR7**	*chemokine (C-C motif) receptor 7*	
**PAPPA**	*pregnancy-associated plasma protein A, pappalysin 1*		**PLA2G16**	*phospholipase A2, group XVI*	
**FCRL3**	*Fc receptor-like 3*		**SHB**	*Src homology 2 domain containing adaptor protein B*	
**PTBP3**	*polypyrimidine tract binding protein 3*		**GPR174**	*G protein-coupled receptor 174*	
**CNP**	*2′,3′-cyclic nucleotide 3′ phosphodiesterase*		**ERP44**	*endoplasmic reticulum protein 44*	✓
**ITGA1**	*integrin, alpha 1*		**GATSL2**	*GATS protein-like 2*	
**FAM96A**	*family with sequence similarity 96, member A*		**RAD51L3-RFFL**	*Uncharacterized protein*	
**DPY19L4**	*dpy-19-like 4 (C. elegans)*		**B3GNT6**	*UDP-GlcNAc:betaGal beta-1,3-N-acetylglucosaminyltransferase 6 (core 3 synthase)*	
**ZC3H10**	*zinc finger CCCH-type containing 10*		**RP11-210M15.2**	*Uncharacterized protein*	
**ZKSCAN5**	*zinc finger with KRAB and SCAN domains 5*		**SYAP1**	*synapse associated protein 1*	
**PGRMC2**	*progesterone receptor membrane component 2*		**ABCA6**	*ATP-binding cassette, sub-family A (ABC1), member 6*	
**GABRA4**	*gamma-aminobutyric acid (GABA) A receptor, alpha 4*		**CLEC14A**	*C-type lectin domain family 14, member A*	
**TRIP11**	*thyroid hormone receptor interactor 11*		**GRM4**	*glutamate receptor, metabotropic 4*	
**EXOC6B**	*exocyst complex component 6B*		**SLC10A6**	*solute carrier family 10 (sodium/bile acid cotransporter), member 6*	

**ZNF442**	*zinc finger protein 442*		**C14orf164**	*chromosome 14 open reading frame 164*	
**GPRC5B**	*G protein-coupled receptor, family C, group 5, member B*		**SPN**	*sialophorin*	
**ARF6**	*ADP-ribosylation factor 6*		**DBNL**	*drebrin-like*	
**TOR1AIP2**	*torsin A interacting protein 2*		**TRIM55**	*tripartite motif containing 55*	
**C10orf118**	*chromosome 10 open reading frame 118*		**MAP7**	*microtubule-associated protein 7*	
**RTKN2**	*rhotekin 2*		**CENPF**	*centromere protein F, 350/400kDa*	
**CLDN12**	*claudin 12*		**CLGN**	*calmegin*	
**CDCA5**	*cell division cycle associated 5*		**TRAT1**	*T cell receptor associated transmembrane adaptor 1*	
**ITGAM**	*integrin, alpha M (complement component 3 receptor 3 subunit)*		**BTG3**	*BTG family, member 3*	
**APOL6**	*apolipoprotein L, 6*		**IGSF6**	*immunoglobulin superfamily, member 6*	
**ZDHHC22**	*zinc finger, DHHC-type containing 22*	✓	**GABRB1**	*gamma-aminobutyric acid (GABA) A receptor, beta 1*	
**C3orf72**	*chromosome 3 open reading frame 72*		**REPS1**	*RALBP1 associated Eps domain containing 1*	
**ARL10**	*ADP-ribosylation factor-like 10*		**FHL5**	*four and a half LIM domains 5*	
**P4HA2**	*prolyl 4-hydroxylase, alpha polypeptide II*		**FZD8**	*frizzled family receptor 8*	
**ZCCHC14**	*zinc finger, CCHC domain containing 14*		**ANKHD1**	*ankyrin repeat and KH domain containing 1*	
**INPP5B**	*inositol polyphosphate-5-phosphatase, 75kDa*		**RAD51**	*RAD51 recombinase*	
**TFEC**	*transcription factor EC*		**THOC5**	*THO complex 5*	
**LGSN**	*lengsin, lens protein with glutamine synthetase domain*		**MSH3**	*mutS homolog 3*	
**ARHGAP20**	*Rho GTPase activating protein 20*		**ELK1**	*ELK1, member of ETS oncogene family*	
**ITPRIPL1**	*inositol 1,4,5-trisphosphate receptor interacting protein-like 1*		**CDH18**	*cadherin 18, type 2*	
**THOC1**	*THO complex 1*		**LMAN2L**	*lectin, mannose-binding 2-like*	
**TBRG1**	*transforming growth factor beta regulator 1*		**F5**	*coagulation factor V (proaccelerin, labile factor)*	
**ZNF286A**	*Zinc finger protein 286A*		**METTL24**	*methyltransferase like 24*	
**RIMBP3C**	*RIMS binding protein 3C*		**GRIN2A**	*glutamate receptor, ionotropic, N-methyl D-aspartate 2A*	

**CCDC15**	*coiled-coil domain containing 15*		**PFN4**	*profilin family, member 4*	
**TREML4**	*triggering receptor expressed on myeloid cells-like 4*		**MTMR8**	*myotubularin related protein 8*	
**CHRDL1**	*chordin-like 1*		**CUL4A**	*cullin 4A*	
**KIAA0907**	*KIAA0907*		**KLRG1**	*killer cell lectin-like receptor subfamily G, member 1*	
**CEACAM7**	*carcinoembryonic antigen-related cell adhesion molecule 7*		**PSMB2**	*proteasome (prosome, macropain) subunit, beta type, 2*	
**ZNF627**	*zinc finger protein 627*		**ZBTB20**	*zinc finger and BTB domain containing 20*	
**TMED7**	*transmembrane emp24 protein transport domain containing 7*		**SELPLG**	*selectin P ligand*	
**RP11-111M22.2**	*Homo sapiens putative uncharacterized protein FLJ37770-like (LOC100506127), mRNA.*		**PRPF4**	*pre-mRNA processing factor 4*	
**ECM1**	*extracellular matrix protein 1*		**ABRACL**	*ABRA C-terminal like*	
**ROBO4**	*roundabout, axon guidance receptor, homolog 4 (Drosophila)*		**NRXN3**	*neurexin 3*	
**TAF2**	*TAF2 RNA polymerase II, TATA box binding protein (TBP)-associated factor, 150kDa*		**CYTIP**	*cytohesin 1 interacting protein*	
**RBPJ**	*recombination signal binding protein for immunoglobulin kappa J region*		**EMC1**	*ER membrane protein complex subunit 1*	
**BCAS3**	*breast carcinoma amplified sequence 3*		**SIGLEC14**	*sialic acid binding Ig-like lectin 14*	
**TADA2A**	*transcriptional adaptor 2A*		**MARCH6**	*membrane-associated ring finger (C3HC4) 6, E3 ubiquitin protein ligase*	
**THSD7A**	*thrombospondin, type I, domain containing 7A*		**MTHFD1L**	*methylenetetrahydrofolate dehydrogenase (NADP+ dependent) 1-like*	
**TRPM8**	*transient receptor potential cation channel, subfamily M, member 8*		**DNAH9**	*dynein, axonemal, heavy chain 9*	
**CS**	*citrate synthase*		**SOBP**	*sine oculis binding protein homolog (Drosophila)*	✓
**TPM4**	*tropomyosin 4*		**UGT2B10**	*UDP glucuronosyltransferase 2 family, polypeptide B10*	
**UBE2B**	*ubiquitin-conjugating enzyme E2B*		**SPTLC3**	*serine palmitoyltransferase, long chain base subunit 3*	
**FAM117B**	*family with sequence similarity 117, member B*		**MED16**	*mediator complex subunit 16*	
**AC068987.1**	*HCG1997999; cDNA FLJ33996 fis, clone DFNES2008881*		**EXT1**	*exostosin glycosyltransferase 1*	
**LRRC10**	*leucine rich repeat containing 10*		**GOLGA7B**	*golgin A7 family, member B*	
**IL6R**	*interleukin 6 receptor*		**DCAF12L2**	*DDB1 and CUL4 associated factor 12-like 2*	

**ANO3**	*anoctamin 3*		**TYRP1**	*tyrosinase-related protein 1*	
**ZNF286B**	*zinc finger protein 286B*		**DDX3X**	*DEAD (Asp-Glu-Ala-Asp) box helicase 3, X-linked*	
**C15orf56**	*chromosome 15 open reading frame 56*		**HDAC2**	*histone deacetylase 2*	
**PDPR**	*pyruvate dehydrogenase phosphatase regulatory subunit*		**IRF2BP2**	*interferon regulatory factor 2 binding protein 2*	
**CNST**	*consortin, connexin sorting protein*		**DKK3**	*dickkopf WNT signaling pathway inhibitor 3*	
**CCNT2**	*cyclin T2*		**NDUFB5**	*NADH dehydrogenase (ubiquinone) 1 beta subcomplex, 5, 16kDa*	
**SOS1**	*son of sevenless homolog 1 (Drosophila)*		**LRP8**	*low density lipoprotein receptor-related protein 8, apolipoprotein e receptor*	
**ZMYM1**	*zinc finger, MYM-type 1*		**TCF23**	*transcription factor 23*	
**ONECUT1**	*one cut homeobox 1*		**PRMT7**	*protein arginine methyltransferase 7*	
**RYBP**	*RING1 and YY1 binding protein*		**XRCC5**	*X-ray repair complementing defective repair in Chinese hamster cells 5 (double-strand-break rejoining)*	✓
**ZDBF2**	*zinc finger, DBF-type containing 2*		**SSTR2**	*somatostatin receptor 2*	
**ZDHHC20**	*zinc finger, DHHC-type containing 20*		**MGAT4C**	*mannosyl (alpha-1,3-)-glycoprotein beta-1,4-N-acetylglucosaminyltransferase, isozyme C (putative)*	
**C1orf226**	*chromosome 1 open reading frame 226*		**SHMT1**	*serine hydroxymethyltransferase 1 (soluble)*	
**ST6GAL2**	*ST6 beta-galactosamide alpha-2,6-sialyltranferase 2*		**ABCF2**	*ATP-binding cassette, sub-family F (GCN20), member 2*	✓
**C12orf60**	*chromosome 12 open reading frame 60*		**SEMA4F**	*sema domain, immunoglobulin domain (Ig), transmembrane domain (TM) and short cytoplasmic domain, (semaphorin) 4F*	
**HNRNPU**	*heterogeneous nuclear ribonucleoprotein U (scaffold attachment factor A)*		**SHC3**	*SHC (Src homology 2 domain containing) transforming protein 3*	
**KCNK1**	*potassium channel, subfamily K, member 1*		**THAP8**	*THAP domain containing 8*	
**RNF150**	*ring finger protein 150*		**CCDC127**	*coiled-coil domain containing 127*	
**ZNF10**	*zinc finger protein 10*		**CREB3L2**	*cAMP responsive element binding protein 3-like 2*	
**ZNF226**	*zinc finger protein 226*		**MAGEB10**	*melanoma antigen family B, 10*	
**TBC1D8B**	*TBC1 domain family, member 8B (with GRAM domain)*		**DCC**	*deleted in colorectal carcinoma*	
**RAD52**	*RAD52 homolog (S. cerevisiae)*		**UBE2D3**	*ubiquitin-conjugating enzyme E2D 3*	
**LARP4B**	*La ribonucleoprotein domain family, member 4B*		**CPPED1**	*calcineurin-like phosphoesterase domain containing 1*	

**ADAT1**	*adenosine deaminase, tRNA-specific 1*		**LAMA4**	*laminin, alpha 4*	
**TMEM189-UBE2V1**	*TMEM189-UBE2V1 readthrough*	✓	**BBS7**	*Bardet-Biedl syndrome 7*	
**CNRIP1**	*cannabinoid receptor interacting protein 1*		**INTS6**	*integrator complex subunit 6*	
**KIAA0825**	*KIAA0825*		**TRIM9**	*tripartite motif containing 9*	
**TMEM189**	*transmembrane protein 189*	✓	**FAM229B**	*family with sequence similarity 229, member B*	
**FAM20B**	*family with sequence similarity 20, member B*	✓	**POLR3H**	*polymerase (RNA) III (DNA directed) polypeptide H (22.9kD)*	
**CXorf56**	*chromosome X open reading frame 56*		**GPR26**	*G protein-coupled receptor 26*	
**RPS3**	*ribosomal protein S3*		**RNF187**	*ring finger protein 187*	
**NUP62CL**	*nucleoporin 62kDa C-terminal like*		**NQO2**	*NAD(P)H dehydrogenase, quinone 2*	
**SORT1**	*sortilin 1*		**LRRFIP1**	*leucine rich repeat (in FLII) interacting protein 1*	
**CLDN18**	*claudin 18*		**RYK**	*receptor-like tyrosine kinase*	
**C12orf61**	*chromosome 12 open reading frame 61*		**CBLN2**	*cerebellin 2 precursor*	
**GAN**	*gigaxonin*		**BST1**	*bone marrow stromal cell antigen 1*	
**ARHGEF37**	*Rho guanine nucleotide exchange factor (GEF) 37*		**GRIK3**	*glutamate receptor, ionotropic, kainate 3*	
**GCSAML**	*germinal center-associated, signaling and motility-like*		**FBXO47**	*F-box protein 47*	
**TXLNB**	*taxilin beta*		**CLIC4**	*chloride intracellular channel 4*	
**PDGFA**	*platelet-derived growth factor alpha polypeptide*		**IFIT1**	*interferon-induced protein with tetratricopeptide repeats 1*	
**EFCAB6**	*EF-hand calcium binding domain 6*		**MBNL1**	*muscleblind-like splicing regulator 1*	
**AGPS**	*alkylglycerone phosphate synthase*		**CRISPLD2**	*cysteine-rich secretory protein LCCL domain containing 2*	
**ATP11C**	*ATPase, class VI, type 11C*		**RBM23**	*RNA binding motif protein 23*	
**SLC22A15**	*solute carrier family 22, member 15*		**CCNB1**	*cyclin B1*	
**CERKL**	*ceramide kinase-like*		**METAP1**	*methionyl aminopeptidase 1*	
**ABLIM1**	*actin binding LIM protein 1*		**JDP2**	*Jun dimerization protein 2*	
**UBR3**	*ubiquitin protein ligase E3 component n-recognin 3 (putative)*		**DLGAP3**	*discs, large (Drosophila) homolog-associated protein 3*	

**NPLOC4**	*nuclear protein localization 4 homolog (S. cerevisiae)*		**KIAA1551**	*KIAA1551*	
**PCGF3**	*polycomb group ring finger 3*		**CANX**	*calnexin*	
**FRMD3**	*FERM domain containing 3*		**LOXL3**	*lysyl oxidase-like 3*	
**ARHGEF19**	*Rho guanine nucleotide exchange factor (GEF) 19*		**NCKIPSD**	*NCK interacting protein with SH3 domain*	
**OGN**	*osteoglycin*		**CLU**	*clusterin*	
**CELSR1**	*cadherin, EGF LAG seven-pass G-type receptor 1*		**PANK1**	*pantothenate kinase 1*	
**SS18L2**	*synovial sarcoma translocation gene on chromosome 18-like 2*		**MYO10**	*myosin X*	
**DDI2**	*DNA-damage inducible 1 homolog 2 (S. cerevisiae)*		**COPS8**	*COP9 signalosome subunit 8*	
**TMEM26**	*transmembrane protein 26*		**ANGPTL3**	*angiopoietin-like 3*	
**MCUR1**	*mitochondrial calcium uniporter regulator 1*		**SPATA6L**	*spermatogenesis associated 6-like*	
**RUNX1T1**	*runt-related transcription factor 1; translocated to, 1 (cyclin D-related)*		**CHIC2**	*cysteine-rich hydrophobic domain 2*	
**LPPR4**	*Lipid phosphate phosphatase-related protein type 4*		**ADCYAP1R1**	*adenylate cyclase activating polypeptide 1 (pituitary) receptor type I*	
**TMLHE**	*trimethyllysine hydroxylase, epsilon*		**WDR75**	*WD repeat domain 75*	
**TMEM65**	*transmembrane protein 65*		**FUCA2**	*fucosidase, alpha-L- 2, plasma*	
**GRIN3A**	*glutamate receptor, ionotropic, N-methyl-D-aspartate 3A*		**ANG**	*angiogenin, ribonuclease, RNase A family, 5*	
**SCN3B**	*sodium channel, voltage-gated, type III, beta subunit*		**HLA-DPB1**	*major histocompatibility complex, class II, DP beta 1*	
**PEX13**	*peroxisomal biogenesis factor 13*		**CIRH1A**	*cirrhosis, autosomal recessive 1A (cirhin)*	
**PLK4**	*polo-like kinase 4*		**GPR35**	*G protein-coupled receptor 35*	
**COL4A3**	*collagen, type IV, alpha 3 (Goodpasture antigen)*		**MBL2**	*mannose-binding lectin (protein C) 2, soluble*	
**PTGER3**	*prostaglandin E receptor 3 (subtype EP3)*		**DUSP3**	*dual specificity phosphatase 3*	
**TMEM196**	*transmembrane protein 196*		**PPL**	*periplakin*	
**CASD1**	*CAS1 domain containing 1*		**ICA1L**	*islet cell autoantigen 1,69kDa-like*	
**ATP8B1**	*ATPase, aminophospholipid transporter, class I, type 8B, member 1*		**KIAA0408**	*KIAA0408*	
**ALPK3**	*alpha-kinase 3*		**UQCR11**	*ubiquinol-cytochrome c reductase, complex III subunit XI*	

**ZNF236**	*zinc finger protein 236*		**SLFN12**	*schlafen family member 12*	
**GPR156**	*G protein-coupled receptor 156*		**LRRC34**	*leucine rich repeat containing 34*	
**PAK3**	*p21 protein (Cdc42/Rac)-activated kinase 3*		**RP11-204N11.1**	*Uncharacterized protein*	
**LDHA**	*lactate dehydrogenase A*		**EXOSC6**	*exosome component 6*	
**TRAF3IP2**	*TRAF3 interacting protein 2*		**N4BP2L1**	*NEDD4 binding protein 2-like 1*	
**TMEM132B**	*transmembrane protein 132B*		**ZNF189**	*zinc finger protein 189*	
**XK**	*X-linked Kx blood group (McLeod syndrome)*		**FECH**	*ferrochelatase*	
**RASGEF1A**	*RasGEF domain family, member 1A*		**TMEM106C**	*transmembrane protein 106C*	
**TNR**	*tenascin R*		**SOX17**	*SRY (sex determining region Y)-box 17*	
**MYLK**	*myosin light chain kinase*		**BAZ2B**	*bromodomain adjacent to zinc finger domain, 2B*	
**AL161915.1**	*Uncharacterized protein*		**DDRGK1**	*DDRGK domain containing 1*	
**TANC1**	*tetratricopeptide repeat, ankyrin repeat and coiled-coil containing 1*		**PCNXL2**	*pecanex-like 2 (Drosophila)*	
**CHRNA3**	*cholinergic receptor, nicotinic, alpha 3 (neuronal)*		**ZNF70**	*zinc finger protein 70*	
**MYL9**	*myosin, light chain 9, regulatory*		**EHD3**	*EH-domain containing 3*	
**RAB22A**	*RAB22A, member RAS oncogene family*		**ZNF140**	*zinc finger protein 140*	
**RFX3**	*regulatory factor X, 3 (influences HLA class II expression)*		**NDNF**	*neuron-derived neurotrophic factor*	
**MAOA**	*monoamine oxidase A*		**CLTC**	*clathrin, heavy chain (Hc)*	
**CLSPN**	*claspin*		**PNP**	*purine nucleoside phosphorylase*	
**RORB**	*RAR-related orphan receptor B*		**SFMBT1**	*Scm-like with four mbt domains 1*	
**CYTH1**	*cytohesin 1*		**ZFP36L2**	*ZFP36 ring finger protein-like 2*	
**LRRC40**	*leucine rich repeat containing 40*		**JAKMIP2**	*janus kinase and microtubule interacting protein 2*	
**CBX1**	*chromobox homolog 1*		**UQCRQ**	*ubiquinol-cytochrome c reductase, complex III subunit VII, 9.5kDa*	
**SCYL3**	*SCY1-like 3 (S. cerevisiae)*		**RPN2**	*ribophorin II*	
**SYT14**	*synaptotagmin XIV*		**PSMB9**	*proteasome (prosome, macropain) subunit, beta type, 9*	

**PPAT**	*phosphoribosyl pyrophosphate amidotransferase*		**PDLIM3**	*PDZ and LIM domain 3*	
**TMEM59**	*transmembrane protein 59*		**DLD**	*dihydrolipoamide dehydrogenase*	
**GPR183**	*G protein-coupled receptor 183*		**MAML3**	*mastermind-like 3 (Drosophila)*	
**TSHR**	*thyroid stimulating hormone receptor*				

Table S3. Predicted and validated mRNAs targeted by miR-15b-5p. A total of 1515 transcripts were found to potentially be targeted by miR-15b-5p, with a total of 1769 sites, between them 738 were experimentally validated (indicated in table by a check mark). Data were obtained by a combined bioinformatical approach using TargetScan Human v8.0 to identify mRNAs predicted to be bounded by miR-584-5p and miRTarBase release 9 to identify validated miRNA-mRNA interactions. Both databases were accessed on December 6th

**Target gene**	**Gene name**	**Validated**	**Target gene**	**Gene name**	**Validated**
**SIX6**	*SIX homeobox 6*		**LAMC1**	*laminin, gamma 1 (formerly LAMB2)*	✓
**ARL2**	*ADP-ribosylation factor-like 2*		**CIAPIN1**	*cytokine induced apoptosis inhibitor 1*	
**CCNE1**	*cyclin E1*	✓	**LPHN2**	*latrophilin 2*	
**SPRED1**	*sprouty-related, EVH1 domain containing 1*	✓	**MAP3K3**	*mitogen-activated protein kinase kinase kinase 3*	
**PLSCR4**	*phospholipid scramblase 4*	✓	**ISOC1**	*isochorismatase domain containing 1*	
**DNAJB4**	*DnaJ (Hsp40) homolog, subfamily B, member 4*		**GNA12**	*guanine nucleotide binding protein (G protein) alpha 12*	
**WNT3A**	*wingless-type MMTV integration site family, member 3A*		**SLC23A3**	*solute carrier family 23, member 3*	
**TNFSF13B**	*tumor necrosis factor (ligand) superfamily, member 13b*		**ABTB2**	*ankyrin repeat and BTB (POZ) domain containing 2*	
**ZBTB34**	*zinc finger and BTB domain containing 34*	✓	**CINP**	*cyclin-dependent kinase 2 interacting protein*	
**LSM11**	*LSM11, U7 small nuclear RNA associated*	✓	**ARL5B**	*ADP-ribosylation factor-like 5B*	
**CYB561D1**	*cytochrome b561 family, member D1*		**NBEAL1**	*neurobeachin-like 1*	
**ANO3**	*anoctamin 3*		**TTL**	*tubulin tyrosine ligase*	
**FP15737**			**ABCF3**	*ATP-binding cassette, sub-family F (GCN20), member 3*	
**TMEM74B**	*transmembrane protein 74B*		**UCP2**	*uncoupling protein 2 (mitochondrial, proton carrier)*	

**EDA**	*ectodysplasin A*		**ZBTB10**	*zinc finger and BTB domain containing 10*	✓
**PHF19**	*PHD finger protein 19*	✓	**PAX7**	*paired box 7*	
**WEE1**	*WEE1 homolog (S. pombe)*	✓	**ADAMTS5**	*ADAM metallopeptidase with thrombospondin type 1 motif, 5*	
**BCL2L2**	*BCL2-like 2*		**RAPGEF1**	*Rap guanine nucleotide exchange factor (GEF) 1*	
**WNT7A**	*wingless-type MMTV integration site family, member 7A*		**PBX3**	*pre-B-cell leukemia homeobox 3*	
**PTH**	*parathyroid hormone*		**LRP1B**	*low density lipoprotein receptor-related protein 1B*	
**AQP11**	*aquaporin 11*		**PHLDA3**	*pleckstrin homology-like domain, family A, member 3*	
**MAMSTR**	*MEF2 activating motif and SAP domain containing transcriptional regulator*		**SALL1**	*sal-like 1 (Drosophila)*	✓
**CDCA4**	*cell division cycle associated 4*	✓	**PSKH1**	*protein serine kinase H1*	✓
**HTR2A**	*5-hydroxytryptamine (serotonin) receptor 2A, G protein-coupled*		**EDAR**	*ectodysplasin A receptor*	
**CCDC19**	*coiled-coil domain containing 19*		**FURIN**	*furin (paired basic amino acid cleaving enzyme)*	✓
**APLN**	*apelin*		**NR2C2**	*nuclear receptor subfamily 2, group C, member 2*	✓
**SLC9A6**	*solute carrier family 9, subfamily A (NHE6, cation proton antiporter 6), member 6*	✓	**C9orf69**	*chromosome 9 open reading frame 69*	
**SLC9A8**	*solute carrier family 9, subfamily A (NHE8, cation proton antiporter 8), member 8*		**RGPD6**	*RANBP2-like and GRIP domain containing 6*	
**SNCG**	*synuclein, gamma (breast cancer-specific protein 1)*		**PELI2**	*pellino E3 ubiquitin protein ligase family member 2*	
**NEK10**	*NIMA-related kinase 10*		**SOS2**	*son of sevenless homolog 2 (Drosophila)*	
**MYB**	*v-myb avian myeloblastosis viral oncogene homolog*		**VAMP7**	*vesicle-associated membrane protein 7*	
**DESI1**	*desumoylating isopeptidase 1*		**TNFAIP1**	*tumor necrosis factor, alpha-induced protein 1 (endothelial)*	
**BTLA**	*B and T lymphocyte associated*		**UBL3**	*ubiquitin-like 3*	
**KRTAP4-4**	*keratin associated protein 4-4*		**ANKRD34A**	*ankyrin repeat domain 34A*	
**SPRYD3**	*SPRY domain containing 3*		**CDK6**	*cyclin-dependent kinase 6*	✓
**ZNF622**	*zinc finger protein 622*	✓	**SUCO**	*SUN domain containing ossification factor*	
**UBE2V1**	*ubiquitin-conjugating enzyme E2 variant 1*	✓	**EN2**	*engrailed homeobox 2*	✓

**RBM6**	*RNA binding motif protein 6*		**FRS2**	*fibroblast growth factor receptor substrate 2*	
**OMG**	*oligodendrocyte myelin glycoprotein*		**FZD4**	*frizzled family receptor 4*	
**VAPB**	*VAMP (vesicle-associated membrane protein)-associated protein B and C*		**OCRL**	*oculocerebrorenal syndrome of Lowe*	✓
**FGF7**	*fibroblast growth factor 7*		**ATG13**	*autophagy related 13*	
**TMEM100**	*transmembrane protein 100*	✓	**AHNAK2**	*AHNAK nucleoprotein 2*	✓
**EMC4**	*ER membrane protein complex subunit 4*	✓	**NOS1**	*nitric oxide synthase 1 (neuronal)*	
**CDC42SE2**	*CDC42 small effector 2*	✓	**KIF3B**	*kinesin family member 3B*	✓
**RAB9B**	*RAB9B, member RAS oncogene family*		**ZBTB9**	*zinc finger and BTB domain containing 9*	
**RASGEF1B**	*RasGEF domain family, member 1B*		**WAPAL**	*wings apart-like homolog (Drosophila)*	
**C2orf42**	*chromosome 2 open reading frame 42*	✓	**UBE2W**	*ubiquitin-conjugating enzyme E2W (putative)*	
**PURA**	*purine-rich element binding protein A*	✓	**ZNF367**	*zinc finger protein 367*	
**SIRT4**	*sirtuin 4*	✓	**PPP2R1A**	*protein phosphatase 2, regulatory subunit A, alpha*	
**KDSR**	*3-ketodihydrosphingosine reductase*		**PDAP1**	*PDGFA associated protein 1*	
**PTPN3**	*protein tyrosine phosphatase, non-receptor type 3*		**ADAMTS3**	*ADAM metallopeptidase with thrombospondin type 1 motif, 3*	
**CDC37L1**	*cell division cycle 37-like 1*	✓	**SALL3**	*sal-like 3 (Drosophila)*	
**GNAI3**	*guanine nucleotide binding protein (G protein), alpha inhibiting activity polypeptide 3*		**ADAMTSL3**	*ADAMTS-like 3*	
**FBXO21**	*F-box protein 21*		**KATNB1**	*katanin p80 (WD repeat containing) subunit B 1*	
**AKT3**	*v-akt murine thymoma viral oncogene homolog 3*	✓	**POLR3D**	*polymerase (RNA) III (DNA directed) polypeptide D, 44kDa*	
**C1QL3**	*complement component 1, q subcomponent-like 3*		**MT1H**	*metallothionein 1H*	
**CNOT6L**	*CCR4-NOT transcription complex, subunit 6-like*		**C14orf1**	*chromosome 14 open reading frame 1*	
**C1orf21**	*chromosome 1 open reading frame 21*	✓	**PIP4K2C**	*phosphatidylinositol-5-phosphate 4-kinase, type II, gamma*	
**ZNF697**	*zinc finger protein 697*		**AIFM1**	*apoptosis-inducing factor, mitochondrion-associated, 1*	
**PAPPA**	*pregnancy-associated plasma protein A, pappalysin 1*		**ARHGAP18**	*Rho GTPase activating protein 18*	
**ZBTB16**	*zinc finger and BTB domain containing 16*	✓	**AKAP12**	*A kinase (PRKA) anchor protein 12*	

**KCNJ2**	*potassium inwardly-rectifying channel, subfamily J, member 2*		**SMIM13**	*small integral membrane protein 13*	
**SMPD1**	*sphingomyelin phosphodiesterase 1, acid lysosomal*		**CACNB1**	*calcium channel, voltage-dependent, beta 1 subunit*	
**CCND1**	*cyclin D1*	✓	**ANKRD13B**	*ankyrin repeat domain 13B*	✓
**AC068987.1**	*HCG1997999; cDNA FLJ33996 fis, clone DFNES2008881*		**AMMECR1**	*Alport syndrome, mental retardation, midface hypoplasia and elliptocytosis chromosomal region gene 1*	
**BTG2**	*BTG family, member 2*	✓	**TASP1**	*taspase, threonine aspartase, 1*	✓
**TMEM55A**	*transmembrane protein 55A*		**MINOS1**	*mitochondrial inner membrane organizing system 1*	
**RSPO3**	*R-spondin 3*		**TMEM248**	*transmembrane protein 248*	
**AXIN2**	*axin 2*	✓	**PEX5**	*peroxisomal biogenesis factor 5*	
**SETD3**	*SET domain containing 3*		**LRRN3**	*leucine rich repeat neuronal 3*	
**RNF144B**	*ring finger protein 144B*		**GABPA**	*GA binding protein transcription factor, alpha subunit 60kDa*	✓
**GPR63**	*G protein-coupled receptor 63*		**SLC12A2**	*solute carrier family 12 (sodium/potassium/chloride transporter), member 2*	
**SMAD7**	*SMAD family member 7*	✓	**CSF1**	*colony stimulating factor 1 (macrophage)*	
**FAM133B**	*family with sequence similarity 133, member B*		**AGO3**	*argonaute RISC catalytic component 3*	
**N4BP1**	*NEDD4 binding protein 1*	✓	**ELMO2**	*engulfment and cell motility 2*	
**CACUL1**	*CDK2-associated, cullin domain 1*	✓	**ZBTB37**	*zinc finger and BTB domain containing 37*	
**CCND2**	*cyclin D2*	✓	**GATA4**	*GATA binding protein 4*	
**TFCP2L1**	*transcription factor CP2-like 1*		**A4GNT**	*alpha-1,4-N-acetylglucosaminyltransferase*	
**KCNN4**	*potassium intermediate/small conductance calcium-activated channel, subfamily N, member 4*		**PPM1A**	*protein phosphatase, Mg2+/Mn2+ dependent, 1A*	✓
**IPPK**	*inositol 1,3,4,5,6-pentakisphosphate 2-kinase*	✓	**GCC2**	*GRIP and coiled-coil domain containing 2*	
**WIPI2**	*WD repeat domain, phosphoinositide interacting 2*	✓	**PHF20**	*PHD finger protein 20*	
**PDK4**	*pyruvate dehydrogenase kinase, isozyme 4*	✓	**DZIP1**	*DAZ interacting zinc finger protein 1*	
**AK4**	*adenylate kinase 4*		**FAT3**	*FAT atypical cadherin 3*	

**TGIF2**	*TGFB-induced factor homeobox 2*		**AMIGO3**	*adhesion molecule with Ig-like domain 3*	
**MKNK1**	*MAP kinase interacting serine/threonine kinase 1*		**CDV3**	*CDV3 homolog (mouse)*	✓
**PPAP2A**	*phosphatidic acid phosphatase type 2A*		**NAV1**	*neuron navigator 1*	
**TLK1**	*tousled-like kinase 1*	✓	**AMIGO1**	*adhesion molecule with Ig-like domain 1*	
**PCDHA11**	*protocadherin alpha 11*		**EBI3**	*Epstein-Barr virus induced 3*	
**PTPN4**	*protein tyrosine phosphatase, non-receptor type 4 (megakaryocyte)*		**SCN4B**	*sodium channel, voltage-gated, type IV, beta subunit*	
**ATG9A**	*autophagy related 9A*	✓	**FRYL**	*FRY-like*	✓
**KIF23**	*kinesin family member 23*	✓	**SLC22A17**	*solute carrier family 22, member 17*	
**UNC80**	*unc-80 homolog (C. elegans)*		**RSF1**	*remodeling and spacing factor 1*	
**DGCR2**	*DiGeorge syndrome critical region gene 2*		**CAPN6**	*calpain 6*	
**HMGA2**	*high mobility group AT-hook 2*		**SAMD4A**	*sterile alpha motif domain containing 4A*	
**USP25**	*ubiquitin specific peptidase 25*		**CBX6**	*chromobox homolog 6*	✓
**PCDHAC2**	*protocadherin alpha subfamily C, 2*		**AGAP2-AS1**	*AGAP2 antisense RNA 1*	
**MOB4**	*MOB family member 4, phocein*	✓	**SRSF10**	*serine/arginine-rich splicing factor 10*	
**VEGFA**	*vascular endothelial growth factor A*	✓	**NOTCH2**	*notch 2*	✓
**SEPT2**	*septin 2*	✓	**ADAP1**	*ArfGAP with dual PH domains 1*	
**FAM73A**	*family with sequence similarity 73, member A*	✓	**PTPRJ**	*protein tyrosine phosphatase, receptor type, J*	✓
**HTR4**	*5-hydroxytryptamine (serotonin) receptor 4, G protein-coupled*		**SLC39A10**	*solute carrier family 39 (zinc transporter), member 10*	
**ARL3**	*ADP-ribosylation factor-like 3*		**FAM81A**	*family with sequence similarity 81, member A*	
**ELL**	*elongation factor RNA polymerase II*		**MTFR1L**	*mitochondrial fission regulator 1-like*	✓
**SYS1**	*SYS1 Golgi-localized integral membrane protein homolog (S. cerevisiae)*		**SLITRK6**	*SLIT and NTRK-like family, member 6*	
**CDK5R1**	*cyclin-dependent kinase 5, regulatory subunit 1 (p35)*		**RP11-73M18.2**	*Kinesin light chain 1*	
**MAFK**	*v-maf avian musculoaponeurotic fibrosarcoma oncogene homolog K*	✓	**ESRRG**	*estrogen-related receptor gamma*	
**KIF1B**	*kinesin family member 1B*		**FAM65B**	*family with sequence similarity 65, member B*	
**SLC35G1**	*solute carrier family 35, member G1*		**DDX54**	*DEAD (Asp-Glu-Ala-Asp) box polypeptide 54*	

**GSKIP**	*GSK3B interacting protein*		**FAM58A**	*family with sequence similarity 58, member A*	
**PEX13**	*peroxisomal biogenesis factor 13*	✓	**CD69**	*CD69 molecule*	
**SHOC2**	*soc-2 suppressor of clear homolog (C. elegans)*	✓	**SENP5**	*SUMO1/sentrin specific peptidase 5*	
**RNF138**	*ring finger protein 138, E3 ubiquitin protein ligase*	✓	**SORT1**	*sortilin 1*	
**CAPZA2**	*capping protein (actin filament) muscle Z-line, alpha 2*	✓	**WWC1**	*WW and C2 domain containing 1*	
**RUNX1T1**	*runt-related transcription factor 1; translocated to, 1 (cyclin D-related)*	✓	**GJA9**	*gap junction protein, alpha 9, 59kDa*	
**CBX4**	*chromobox homolog 4*	✓	**ADORA2A**	*adenosine A2a receptor*	
**SRPR**	*signal recognition particle receptor (docking protein)*	✓	**SLC35A4**	*solute carrier family 35, member A4*	
**RPL14**	*ribosomal protein L14*	✓	**RASSF5**	*Ras association (RalGDS/AF-6) domain family member 5*	✓
**KLHL18**	*kelch-like family member 18*		**VPS37C**	*vacuolar protein sorting 37 homolog C (S. cerevisiae)*	
**PLAG1**	*pleiomorphic adenoma gene 1*	✓	**ZNF654**	*zinc finger protein 654*	
**AMER1**	*APC membrane recruitment protein 1*	✓	**GDI2**	*GDP dissociation inhibitor 2*	
**ZMAT3**	*zinc finger, matrin-type 3*	✓	**HSPG2**	*heparan sulfate proteoglycan 2*	
**SLC7A2**	*solute carrier family 7 (cationic amino acid transporter, y+ system), member 2*		**PUF60**	*poly-U binding splicing factor 60KDa*	
**TEX19**	*testis expressed 19*		**CGNL1**	*cingulin-like 1*	
**RASSF8**	*Ras association (RalGDS/AF-6) domain family (N-terminal) member 8*		**CSDE1**	*cold shock domain containing E1, RNA-binding*	✓
**ANXA11**	*annexin A11*		**RGMA**	*RGM domain family, member A*	
**SH3GL2**	*SH3-domain GRB2-like 2*		**FAM189A1**	*family with sequence similarity 189, member A1*	
**BTAF1**	*BTAF1 RNA polymerase II, B-TFIID transcription factor-associated, 170kDa*	✓	**FKRP**	*fukutin related protein*	
**CRKL**	*v-crk avian sarcoma virus CT10 oncogene homolog-like*	✓	**SIK1**	*salt-inducible kinase 1*	✓
**SYNRG**	*synergin, gamma*	✓	**CCDC88C**	*coiled-coil domain containing 88C*	✓
**CLDN12**	*claudin 12*		**CX3CL1**	*chemokine (C-X3-C motif) ligand 1*	
**PTPRR**	*protein tyrosine phosphatase, receptor type, R*		**KIF1C**	*kinesin family member 1C*	

**IKBKB**	*inhibitor of kappa light polypeptide gene enhancer in B-cells, kinase beta*		**CHD6**	*chromodomain helicase DNA binding protein 6*	
**CHEK1**	*checkpoint kinase 1*	✓	**EIF3A**	*eukaryotic translation initiation factor 3, subunit A*	
**TMEM189-UBE2V1**	*TMEM189-UBE2V1 readthrough*	✓	**CD164**	*CD164 molecule, sialomucin*	
**HSPE1-MOB4**	*HSPE1-MOB4 readthrough*	✓	**POM121C**	*POM121 transmembrane nucleoporin C*	✓
**RPS6KA3**	*ribosomal protein S6 kinase, 90kDa, polypeptide 3*	✓	**AOC1**	*amine oxidase, copper containing 1*	
**TMEM189**	*transmembrane protein 189*	✓	**TMC7**	*transmembrane channel-like 7*	✓
**MYBL1**	*v-myb avian myeloblastosis viral oncogene homolog-like 1*		**GPC6**	*glypican 6*	
**SALL4**	*sal-like 4 (Drosophila)*		**CARD10**	*caspase recruitment domain family, member 10*	✓
**ARMC12**	*armadillo repeat containing 12*	✓	**TMPPE**	*transmembrane protein with metallophosphoesterase domain*	
**FGF2**	*fibroblast growth factor 2 (basic)*	✓	**SYTL2**	*synaptotagmin-like 2*	
**FASN**	*fatty acid synthase*	✓	**ERLIN2**	*ER lipid raft associated 2*	
**RAB30**	*RAB30, member RAS oncogene family*		**WDTC1**	*WD and tetratricopeptide repeats 1*	
**HSPA4L**	*heat shock 70kDa protein 4-like*	✓	**ATP10B**	*ATPase, class V, type 10B*	
**PLEKHA5**	*pleckstrin homology domain containing, family A member 5*		**MN1**	*meningioma (disrupted in balanced translocation) 1*	
**PLA2G15**	*phospholipase A2, group XV*		**ACOX1**	*acyl-CoA oxidase 1, palmitoyl*	✓
**MEOX2**	*mesenchyme homeobox 2*		**ITPRIPL2**	*inositol 1,4,5-trisphosphate receptor interacting protein-like 2*	
**CA8**	*carbonic anhydrase VIII*	✓	**GRB10**	*growth factor receptor-bound protein 10*	
**UBE2Q1**	*ubiquitin-conjugating enzyme E2Q family member 1*	✓	**CNNM2**	*cyclin M2*	
**UBE4B**	*ubiquitination factor E4B*		**CENPBD1**	*CENPB DNA-binding domains containing 1*	
**FAM179B**	*family with sequence similarity 179, member B*		**KLHDC10**	*kelch domain containing 10*	✓
**IST1**	*increased sodium tolerance 1 homolog (yeast)*		**LMO7**	*LIM domain 7*	
**ACTR2**	*ARP2 actin-related protein 2 homolog (yeast)*	✓	**MYADM**	*myeloid-associated differentiation marker*	
**DYRK1B**	*dual-specificity tyrosine-(Y)-phosphorylation regulated kinase 1B*		**SETD1B**	*SET domain containing 1B*	✓
**CYB561**	*cytochrome b561*		**ETV3**	*ets variant 3*	

**CCNT2**	*cyclin T2*	✓	**NCKAP1L**	*NCK-associated protein 1-like*	
**SYT3**	*synaptotagmin III*		**BEST4**	*bestrophin 4*	
**KIAA0247**	*KIAA0247*		**PPP1R11**	*protein phosphatase 1, regulatory (inhibitor) subunit 11*	✓
**GAREM**	*GRB2 associated, regulator of MAPK1*		**LRRK1**	*leucine-rich repeat kinase 1*	
**ZBTB46**	*zinc finger and BTB domain containing 46*		**ASB7**	*ankyrin repeat and SOCS box containing 7*	
**RBMS1**	*RNA binding motif, single stranded interacting protein 1*		**RORA**	*RAR-related orphan receptor A*	
**POLR3F**	*polymerase (RNA) III (DNA directed) polypeptide F, 39 kDa*		**MSL1**	*male-specific lethal 1 homolog (Drosophila)*	✓
**MED26**	*mediator complex subunit 26*		**SPOCD1**	*SPOC domain containing 1*	
**PPM1E**	*protein phosphatase, Mg2+/Mn2+ dependent, 1E*		**ZFHX4**	*zinc finger homeobox 4*	✓
**DLL1**	*delta-like 1 (Drosophila)*		**KPNA5**	*karyopherin alpha 5 (importin alpha 6)*	
**UBQLNL**	*ubiquilin-like*		**UBE4A**	*ubiquitination factor E4A*	✓
**SLC2A14**	*solute carrier family 2 (facilitated glucose transporter), member 14*		**FAM160B1**	*family with sequence similarity 160, member B1*	
**TBPL1**	*TBP-like 1*	✓	**IRF2BP1**	*interferon regulatory factor 2 binding protein 1*	
**NDP**	*Norrie disease (pseudoglioma)*		**GRAMD2**	*GRAM domain containing 2*	
**BTRC**	*beta-transducin repeat containing E3 ubiquitin protein ligase*	✓	**NRN1**	*neuritin 1*	
**BPIFA1**	*BPI fold containing family A, member 1*		**ELOVL6**	*ELOVL fatty acid elongase 6*	
**TMEM161B**	*transmembrane protein 161B*	✓	**MNT**	*MNT, MAX dimerization protein*	
**WDR82**	*WD repeat domain 82*		**CRISPLD2**	*cysteine-rich secretory protein LCCL domain containing 2*	
**NFS1**	*NFS1 cysteine desulfurase*		**SEMA5B**	*sema domain, seven thrombospondin repeats (type 1 and type 1-like), transmembrane domain (TM) and short cytoplasmic domain, (semaphorin) 5B*	
**ALOX12**	*arachidonate 12-lipoxygenase*		**FAM171A2**	*family with sequence similarity 171, member A2*	
**GADD45G**	*growth arrest and DNA-damage-inducible, gamma*		**RFWD3**	*ring finger and WD repeat domain 3*	
**FBXW7**	*F-box and WD repeat domain containing 7, E3 ubiquitin protein ligase*		**FSTL1**	*follistatin-like 1*	
**SEMA6D**	*sema domain, transmembrane domain (TM), and cytoplasmic domain, (semaphorin) 6D*		**INPP5J**	*inositol polyphosphate-5-phosphatase J*	

**EPT1**	*ethanolaminephosphotransferase 1 (CDP-ethanolamine-specific)*	✓	**USP14**	*ubiquitin specific peptidase 14 (tRNA-guanine transglycosylase)*	
**RNF24**	*ring finger protein 24*		**CLASP1**	*cytoplasmic linker associated protein 1*	
**SPAG7**	*sperm associated antigen 7*		**CRTC3**	*CREB regulated transcription coactivator 3*	
**VTI1B**	*vesicle transport through interaction with t-SNAREs 1B*		**SFXN2**	*sideroflexin 2*	
**YWHAH**	*tyrosine 3-monooxygenase/tryptophan 5-monooxygenase activation protein, eta polypeptide*	✓	**FBXL20**	*F-box and leucine-rich repeat protein 20*	✓
**E2F7**	*E2F transcription factor 7*	✓	**PCSK5**	*proprotein convertase subtilisin/kexin type 5*	
**WBP11**	*WW domain binding protein 11*		**GPR124**	*G protein-coupled receptor 124*	
**TMEM55B**	*transmembrane protein 55B*	✓	**SKOR1**	*SKI family transcriptional corepressor 1*	
**C3orf27**	*chromosome 3 open reading frame 27*		**SLC38A7**	*solute carrier family 38, member 7*	
**ENTPD7**	*ectonucleoside triphosphate diphosphohydrolase 7*	✓	**NUCKS1**	*nuclear casein kinase and cyclin-dependent kinase substrate 1*	✓
**GABARAPL1**	*GABA(A) receptor-associated protein like 1*	✓	**NFE2L1**	*nuclear factor, erythroid 2-like 1*	
**PAFAH1B1**	*platelet-activating factor acetylhydrolase 1b, regulatory subunit 1 (45kDa)*	✓	**SLC24A3**	*solute carrier family 24 (sodium/potassium/calcium exchanger), member 3*	
**TFAP2D**	*transcription factor AP-2 delta (activating enhancer binding protein 2 delta)*		**MAP3K4**	*mitogen-activated protein kinase kinase kinase 4*	
**KIF21A**	*kinesin family member 21A*		**STAG1**	*stromal antigen 1*	
**RNF125**	*ring finger protein 125, E3 ubiquitin protein ligase*		**KPNA1**	*karyopherin alpha 1 (importin alpha 5)*	✓
**FAM57B**	*family with sequence similarity 57, member B*		**NSF**	*N-ethylmaleimide-sensitive factor*	
**TFRC**	*transferrin receptor*		**UNG**	*uracil-DNA glycosylase*	
**INSR**	*insulin receptor*	✓	**WDFY4**	*WDFY family member 4*	
**PCMT1**	*protein-L-isoaspartate (D-aspartate) O-methyltransferase*	✓	**RAB35**	*RAB35, member RAS oncogene family*	
**TACC1**	*transforming, acidic coiled-coil containing protein 1*		**TRAF3**	*TNF receptor-associated factor 3*	
**WNT2B**	*wingless-type MMTV integration site family, member 2B*		**PRRC2C**	*proline-rich coiled-coil 2C*	✓
**HELZ**	*helicase with zinc finger*		**ADAMTS13**	*ADAM metallopeptidase with thrombospondin type 1 motif, 13*	

**RECK**	*reversion-inducing-cysteine-rich protein with kazal motifs*	✓	**AMOTL1**	*angiomotin like 1*	✓
**RAB9A**	*RAB9A, member RAS oncogene family*		**IDH3A**	*isocitrate dehydrogenase 3 (NAD+) alpha*	
**GABBR1**	*gamma-aminobutyric acid (GABA) B receptor, 1*		**IRF2BPL**	*interferon regulatory factor 2 binding protein-like*	
**TRMT112**	*tRNA methyltransferase 11-2 homolog (S. cerevisiae)*		**KIAA0226**	*KIAA0226*	✓
**KIAA1328**	*KIAA1328*		**PDLIM5**	*PDZ and LIM domain 5*	
**CIDEB**	*cell death-inducing DFFA-like effector b*		**HOXA3**	*homeobox A3*	✓
**DCP1A**	*decapping mRNA 1A*		**TMEM170B**	*transmembrane protein 170B*	
**MMS19**	*MMS19 nucleotide excision repair homolog (S. cerevisiae)*		**MBD1**	*methyl-CpG binding domain protein 1*	
**HIGD1A**	*HIG1 hypoxia inducible domain family, member 1A*	✓	**IL1RAPL1**	*interleukin 1 receptor accessory protein-like 1*	
**ALAD**	*aminolevulinate dehydratase*		**RHOBTB2**	*Rho-related BTB domain containing 2*	
**CPEB2**	*cytoplasmic polyadenylation element binding protein 2*	✓	**SNIP1**	*Smad nuclear interacting protein 1*	
**SGK1**	*serum/glucocorticoid regulated kinase 1*		**SEC24A**	*SEC24 family, member A (S. cerevisiae)*	✓
**VPS4A**	*vacuolar protein sorting 4 homolog A (S. cerevisiae)*	✓	**RBPJ**	*recombination signal binding protein for immunoglobulin kappa J region*	✓
**SLIT2**	*slit homolog 2 (Drosophila)*		**ARRDC4**	*arrestin domain containing 4*	
**PCDHA3**	*protocadherin alpha 3*		**TNIK**	*TRAF2 and NCK interacting kinase*	
**GALNT7**	*UDP-N-acetyl-alpha-D-galactosamine:polypeptide N-acetylgalactosaminyltransferase 7 (GalNAc-T7)*		**CNN1**	*calponin 1, basic, smooth muscle*	
**SNX16**	*sorting nexin 16*		**TSC22D3**	*TSC22 domain family, member 3*	
**CHAC1**	*ChaC, cation transport regulator homolog 1 (E. coli)*		**DYNC1LI2**	*dynein, cytoplasmic 1, light intermediate chain 2*	
**ZNF449**	*zinc finger protein 449*		**TBC1D20**	*TBC1 domain family, member 20*	✓
**CDK8**	*cyclin-dependent kinase 8*		**DIAPH2**	*diaphanous-related formin 2*	
**MIPOL1**	*mirror-image polydactyly 1*		**MACC1**	*metastasis associated in colon cancer 1*	
**PHACTR2**	*phosphatase and actin regulator 2*		**CLCN5**	*chloride channel, voltage-sensitive 5*	
**GORASP2**	*golgi reassembly stacking protein 2, 55kDa*		**NPEPPS**	*aminopeptidase puromycin sensitive*	✓

**PLRG1**	*pleiotropic regulator 1*	✓	**KPNA4**	*karyopherin alpha 4 (importin alpha 3)*	
**AGO4**	*argonaute RISC catalytic component 4*	✓	**LIX1L**	*Lix1 homolog (mouse)-like*	
**STRADB**	*STE20-related kinase adaptor beta*	✓	**PLXNA2**	*plexin A2*	
**CDK17**	*cyclin-dependent kinase 17*	✓	**ZKSCAN1**	*zinc finger with KRAB and SCAN domains 1*	
**BDNF**	*brain-derived neurotrophic factor*		**OTX1**	*orthodenticle homeobox 1*	
**RFX3**	*regulatory factor X, 3 (influences HLA class II expression)*		**TP53INP1**	*tumor protein p53 inducible nuclear protein 1*	
**CARM1**	*coactivator-associated arginine methyltransferase 1*		**TRAK1**	*trafficking protein, kinesin binding 1*	✓
**SCN8A**	*sodium channel, voltage gated, type VIII, alpha subunit*		**CNKSR2**	*connector enhancer of kinase suppressor of Ras 2*	
**PLD1**	*phospholipase D1, phosphatidylcholine-specific*		**SPRY4**	*sprouty homolog 4 (Drosophila)*	
**CDC14A**	*cell division cycle 14A*		**DLL4**	*delta-like 4 (Drosophila)*	
**ATXN7L3**	*ataxin 7-like 3*		**PRKCD**	*protein kinase C, delta*	✓
**PPT2**	*palmitoyl-protein thioesterase 2*		**HOXC11**	*homeobox C11*	
**KIF5A**	*kinesin family member 5A*		**KSR1**	*kinase suppressor of ras 1*	
**FGF18**	*fibroblast growth factor 18*		**PTPRM**	*protein tyrosine phosphatase, receptor type, M*	
**TENM2**	*teneurin transmembrane protein 2*		**PURB**	*purine-rich element binding protein B*	
**ZNF609**	*zinc finger protein 609*		**SEL1L3**	*sel-1 suppressor of lin-12-like 3 (C. elegans)*	
**LUZP1**	*leucine zipper protein 1*	✓	**RSPO2**	*R-spondin 2*	
**MYLK**	*myosin light chain kinase*		**OTOGL**	*otogelin-like*	
**TMCC1**	*transmembrane and coiled-coil domain family 1*		**FOXP2**	*forkhead box P2*	
**CDC27**	*cell division cycle 27*	✓	**NDOR1**	*NADPH dependent diflavin oxidoreductase 1*	
**KIAA1432**	*KIAA1432*		**PAPPA2**	*pappalysin 2*	
**DOLPP1**	*dolichyldiphosphatase 1*		**MIF4GD**	*MIF4G domain containing*	
**GSTCD**	*glutathione S-transferase, C-terminal domain containing*		**PAPD4**	*PAP associated domain containing 4*	
**RAB40AL**	*RAB40A, member RAS oncogene family-like*		**BHLHE41**	*basic helix-loop-helix family, member e41*	
**ZMYM2**	*zinc finger, MYM-type 2*		**GALNT13**	*UDP-N-acetyl-alpha-D-galactosamine:polypeptide N-*	

				acetylgalactosaminyltransferase 13 (GalNAc-T13)	
**IHH**	*indian hedgehog*		**PRKG1**	*protein kinase, cGMP-dependent, type I*	
**HMGA1**	*high mobility group AT-hook 1*	✓	**VAT1**	*vesicle amine transport 1*	
**GYLTL1B**	*glycosyltransferase-like 1B*		**GPRIN3**	*GPRIN family member 3*	
**PDCD11**	*programmed cell death 11*		**LHX4**	*LIM homeobox 4*	
**CCNYL1**	*cyclin Y-like 1*		**HS3ST3B1**	*heparan sulfate (glucosamine) 3-O-sulfotransferase 3B1*	
**CD28**	*CD28 molecule*		**DEDD**	*death effector domain containing*	
**VWC2L**	*von Willebrand factor C domain containing protein 2-like*		**ATP2B2**	*ATPase, Ca++ transporting, plasma membrane 2*	✓
**LITAF**	*lipopolysaccharide-induced TNF factor*	✓	**XPO4**	*exportin 4*	
**USP3**	*ubiquitin specific peptidase 3*	✓	**ZNF532**	*zinc finger protein 532*	
**PPM1D**	*protein phosphatase, Mg2+/Mn2+ dependent, 1D*	✓	**LTBP4**	*latent transforming growth factor beta binding protein 4*	
**CCND3**	*cyclin D3*		**CHD9**	*chromodomain helicase DNA binding protein 9*	
**MTMR11**	*myotubularin related protein 11*		**MLLT6**	*myeloid/lymphoid or mixed-lineage leukemia (trithorax homolog, Drosophila); translocated to, 6*	✓
**ACVR2B**	*activin A receptor, type IIB*		**RIC8A**	*RIC8 guanine nucleotide exchange factor A*	
**LMAN2L**	*lectin, mannose-binding 2-like*		**TRIM29**	*tripartite motif containing 29*	✓
**SCOC**	*short coiled-coil protein*		**KMT2D**	*lysine (K)-specific methyltransferase 2D*	✓
**SLC2A3**	*solute carrier family 2 (facilitated glucose transporter), member 3*	✓	**DEPTOR**	*DEP domain containing MTOR-interacting protein*	
**MAP2K1**	*mitogen-activated protein kinase kinase 1*		**PAX2**	*paired box 2*	
**PSMD7**	*proteasome (prosome, macropain) 26S subunit, non-ATPase, 7*	✓	**KLHL26**	*kelch-like family member 26*	
**DENND6A**	*DENN/MADD domain containing 6A*		**KDR**	*kinase insert domain receptor (a type III receptor tyrosine kinase)*	✓
**AREL1**	*apoptosis resistant E3 ubiquitin protein ligase 1*		**NF1**	*neurofibromin 1*	
**RYBP**	*RING1 and YY1 binding protein*		**DDR1**	*discoidin domain receptor tyrosine kinase 1*	
**SLC41A2**	*solute carrier family 41 (magnesium transporter), member 2*		**BACH2**	*BTB and CNC homology 1, basic leucine zipper transcription factor 2*	

**ISLR**	*immunoglobulin superfamily containing leucine-rich repeat*		**CTDSPL**	*CTD (carboxy-terminal domain, RNA polymerase II, polypeptide A) small phosphatase-like*	✓
**CPEB3**	*cytoplasmic polyadenylation element binding protein 3*	✓	**TLE4**	*transducin-like enhancer of split 4 (E(sp1) homolog, Drosophila)*	✓
**TPD52L3**	*tumor protein D52-like 3*		**IGF2R**	*insulin-like growth factor 2 receptor*	
**TRANK1**	*tetratricopeptide repeat and ankyrin repeat containing 1*		**MTMR4**	*myotubularin related protein 4*	✓
**G0S2**	*G0/G1switch 2*		**C16orf52**	*chromosome 16 open reading frame 52*	
**SMURF2**	*SMAD specific E3 ubiquitin protein ligase 2*		**RPP14**	*ribonuclease P/MRP 14kDa subunit*	
**SCN2A**	*sodium channel, voltage-gated, type II, alpha subunit*		**RIMKLB**	*ribosomal modification protein rimK-like family member B*	
**SYNJ1**	*synaptojanin 1*	✓	**MYLK4**	*myosin light chain kinase family, member 4*	
**MAP7**	*microtubule-associated protein 7*		**SMURF1**	*SMAD specific E3 ubiquitin protein ligase 1*	✓
**SYT4**	*synaptotagmin IV*		**SMYD5**	*SMYD family member 5*	
**DYNLL2**	*dynein, light chain, LC8-type 2*	✓	**LIN7A**	*lin-7 homolog A (C. elegans)*	
**ATXN2**	*ataxin 2*		**AMOT**	*angiomotin*	✓
**SYDE2**	*synapse defective 1, Rho GTPase, homolog 2 (C. elegans)*		**WNK3**	*WNK lysine deficient protein kinase 3*	✓
**RFWD2**	*ring finger and WD repeat domain 2, E3 ubiquitin protein ligase*	✓	**RICTOR**	*RPTOR independent companion of MTOR, complex 2*	
**ELL2**	*elongation factor, RNA polymerase II, 2*		**NCOR2**	*nuclear receptor corepressor 2*	✓
**FAM91A1**	*family with sequence similarity 91, member A1*		**GDPD5**	*glycerophosphodiester phosphodiesterase domain containing 5*	
**DYNC1I1**	*dynein, cytoplasmic 1, intermediate chain 1*		**ETV1**	*ets variant 1*	
**STK33**	*serine/threonine kinase 33*		**SKI**	*v-ski avian sarcoma viral oncogene homolog*	✓
**SOCS6**	*suppressor of cytokine signaling 6*		**CASKIN1**	*CASK interacting protein 1*	✓
**PDCD4**	*programmed cell death 4 (neoplastic transformation inhibitor)*	✓	**RBM12**	*RNA binding motif protein 12*	
**PAM**	*peptidylglycine alpha-amidating monooxygenase*		**ATP13A3**	*ATPase type 13A3*	✓
**C2orf72**	*chromosome 2 open reading frame 72*		**CDC25B**	*cell division cycle 25B*	
**MYT1L**	*myelin transcription factor 1-like*		**UQCC1**	*ubiquinol-cytochrome c reductase complex assembly factor 1*	

**FRY**	*furry homolog (Drosophila)*		**KPNA3**	*karyopherin alpha 3 (importin alpha 4)*	✓
**PLEKHA1**	*pleckstrin homology domain containing, family A (phosphoinositide binding specific) member 1*	✓	**B4GALNT3**	*beta-1,4-N-acetyl-galactosaminyl transferase 3*	
**ARIH1**	*ariadne RBR E3 ubiquitin protein ligase 1*	✓	**DLST**	*dihydrolipoamide S-succinyltransferase (E2 component of 2-oxo-glutarate complex)*	
**CYP26B1**	*cytochrome P450, family 26, subfamily B, polypeptide 1*	✓	**PPFIA3**	*protein tyrosine phosphatase, receptor type, f polypeptide (PTPRF), interacting protein (liprin), alpha 3*	
**ELMSAN1**	*ELM2 and Myb/SANT-like domain containing 1*		**TEAD1**	*TEA domain family member 1 (SV40 transcriptional enhancer factor)*	
**PID1**	*phosphotyrosine interaction domain containing 1*		**GABRE**	*gamma-aminobutyric acid (GABA) A receptor, epsilon*	
**RELN**	*reelin*		**ATF7IP2**	*activating transcription factor 7 interacting protein 2*	
**PARD6B**	*par-6 family cell polarity regulator beta*		**CCNJL**	*cyclin J-like*	
**CACNA2D1**	*calcium channel, voltage-dependent, alpha 2/delta subunit 1*		**FAM134A**	*family with sequence similarity 134, member A*	
**NSG1**	*Neuron-specific protein family member 1*		**ARHGAP17**	*Rho GTPase activating protein 17*	
**MOB3B**	*MOB kinase activator 3B*		**EXOC5**	*exocyst complex component 5*	
**CDC42EP2**	*CDC42 effector protein (Rho GTPase binding) 2*		**ZADH2**	*zinc binding alcohol dehydrogenase domain containing 2*	
**CDC25A**	*cell division cycle 25A*	✓	**CREBL2**	*cAMP responsive element binding protein-like 2*	✓
**IPO7**	*importin 7*		**OS9**	*osteosarcoma amplified 9, endoplasmic reticulum lectin*	
**BZW1**	*basic leucine zipper and W2 domains 1*	✓	**FNDC3B**	*fibronectin type III domain containing 3B*	
**PAQR3**	*progestin and adipoQ receptor family member III*		**RNF152**	*ring finger protein 152*	
**GHR**	*growth hormone receptor*		**KCNAB1**	*potassium voltage-gated channel, shaker-related subfamily, beta member 1*	
**KIAA0226L**	*KIAA0226-like*		**FGF9**	*fibroblast growth factor 9*	
**FOSL2**	*FOS-like antigen 2*		**NFATC3**	*nuclear factor of activated T-cells, cytoplasmic, calcineurin-dependent 3*	
**C16orf72**	*chromosome 16 open reading frame 72*	✓	**BAG4**	*BCL2-associated athanogene 4*	✓
**PNPLA6**	*patatin-like phospholipase domain containing 6*	✓	**BCL11B**	*B-cell CLL/lymphoma 11B (zinc finger protein)*	
**SUMO3**	*small ubiquitin-like modifier 3*		**KIAA0513**	*KIAA0513*	

**CASR**	*calcium-sensing receptor*		**CDK4**	*cyclin-dependent kinase 4*	✓
**BOLA3**	*bolA family member 3*		**STXBP3**	*syntaxin binding protein 3*	✓
**PIM1**	*pim-1 oncogene*	✓	**MYO1C**	*myosin IC*	
**MAPK8**	*mitogen-activated protein kinase 8*		**FBXO27**	*F-box protein 27*	
**GRM7**	*glutamate receptor, metabotropic 7*		**RABAC1**	*Rab acceptor 1 (prenylated)*	
**TMEM255A**	*transmembrane protein 255A*		**ADCY5**	*adenylate cyclase 5*	
**XPO7**	*exportin 7*		**RCOR3**	*REST corepressor 3*	
**TBRG1**	*transforming growth factor beta regulator 1*	✓	**PHF15**	*PHD finger protein 15*	
**FAM122A**	*family with sequence similarity 122A*		**DACH1**	*dachshund homolog 1 (Drosophila)*	
**USP42**	*ubiquitin specific peptidase 42*	✓	**PCGF5**	*polycomb group ring finger 5*	
**RARB**	*retinoic acid receptor, beta*	✓	**WASL**	*Wiskott-Aldrich syndrome-like*	
**DDX3Y**	*DEAD (Asp-Glu-Ala-Asp) box helicase 3, Y-linked*	✓	**RNF14**	*ring finger protein 14*	
**EPHA7**	*EPH receptor A7*		**PPP6R3**	*protein phosphatase 6, regulatory subunit 3*	
**FERMT2**	*fermitin family member 2*		**LMAN2**	*lectin, mannose-binding 2*	
**SEH1L**	*SEH1-like (S. cerevisiae)*		**CACNA1I**	*calcium channel, voltage-dependent, T type, alpha 1I subunit*	
**BFAR**	*bifunctional apoptosis regulator*		**ACSL4**	*acyl-CoA synthetase long-chain family member 4*	
**TUBA1A**	*tubulin, alpha 1a*		**EVI5**	*ecotropic viral integration site 5*	
**SOBP**	*sine oculis binding protein homolog (Drosophila)*		**XPR1**	*xenotropic and polytropic retrovirus receptor 1*	
**QKI**	*QKI, KH domain containing, RNA binding*		**TAOK1**	*TAO kinase 1*	✓
**TTC14**	*tetratricopeptide repeat domain 14*		**GBA2**	*glucosidase, beta (bile acid) 2*	
**ZCCHC3**	*zinc finger, CCHC domain containing 3*	✓	**ZFHX3**	*zinc finger homeobox 3*	
**EYA1**	*eyes absent homolog 1 (Drosophila)*		**STOX2**	*storkhead box 2*	
**TLL1**	*tolloid-like 1*	✓	**ANGEL1**	*angel homolog 1 (Drosophila)*	
**PIEZO1**	*piezo-type mechanosensitive ion channel component 1*		**ATP1B4**	*ATPase, Na+/K+ transporting, beta 4 polypeptide*	
**NXPH1**	*neurexophilin 1*		**C12orf49**	*chromosome 12 open reading frame 49*	

**C20orf112**	*chromosome 20 open reading frame 112*		**ERC2**	*ELKS/RAB6-interacting/CAST family member 2*	
**MAP3K9**	*mitogen-activated protein kinase kinase kinase 9*		**KRAS**	*Kirsten rat sarcoma viral oncogene homolog*	✓
**BTF3**	*basic transcription factor 3*		**MDN1**	*MDN1, midasin homolog (yeast)*	
**VAMP1**	*vesicle-associated membrane protein 1 (synaptobrevin 1)*	✓	**PIK3C2A**	*phosphatidylinositol-4-phosphate 3-kinase, catalytic subunit type 2 alpha*	
**SPRY3**	*sprouty homolog 3 (Drosophila)*		**MON1B**	*MON1 secretory trafficking family member B*	
**IARS**	*isoleucyl-tRNA synthetase*		**ZNF518B**	*zinc finger protein 518B*	
**SNAP25**	*synaptosomal-associated protein, 25kDa*		**LIN28B**	*lin-28 homolog B (C. elegans)*	✓
**FLT3**	*fms-related tyrosine kinase 3*		**RBM23**	*RNA binding motif protein 23*	
**ARPP19**	*cAMP-regulated phosphoprotein, 19kDa*		**ROR1**	*receptor tyrosine kinase-like orphan receptor 1*	
**ACSBG1**	*acyl-CoA synthetase bubblegum family member 1*		**ZNRF3**	*zinc and ring finger 3*	✓
**DMTF1**	*cyclin D binding myb-like transcription factor 1*	✓	**ADAMTS18**	*ADAM metallopeptidase with thrombospondin type 1 motif, 18*	
**KIF5C**	*kinesin family member 5C*		**LPHN1**	*latrophilin 1*	
**TNRC6B**	*trinucleotide repeat containing 6B*	✓	**PTPRD**	*protein tyrosine phosphatase, receptor type, D*	✓
**C11orf24**	*chromosome 11 open reading frame 24*	✓	**ZNF436**	*zinc finger protein 436*	
**FGFR1**	*fibroblast growth factor receptor 1*		**TBL1XR1**	*transducin (beta)-like 1 X-linked receptor 1*	✓
**RAB11FIP2**	*RAB11 family interacting protein 2 (class I)*	✓	**SNX33**	*sorting nexin 33*	
**RAD23B**	*RAD23 homolog B (S. cerevisiae)*	✓	**SYDE1**	*synapse defective 1, Rho GTPase, homolog 1 (C. elegans)*	
**ATXN7L1**	*ataxin 7-like 1*		**MED20**	*mediator complex subunit 20*	
**PLXNC1**	*plexin C1*		**GIT1**	*G protein-coupled receptor kinase interacting ArfGAP 1*	
**SESN1**	*sestrin 1*		**NLRP1**	*NLR family, pyrin domain containing 1*	
**RTN4**	*reticulon 4*	✓	**FOXO1**	*forkhead box O1*	✓
**MIB1**	*mindbomb E3 ubiquitin protein ligase 1*	✓	**UBE2QL1**	*ubiquitin-conjugating enzyme E2Q family-like 1*	
**RNF217**	*ring finger protein 217*		**RP11-192H23.4**	*Uncharacterized protein*	
**CNTNAP1**	*contactin associated protein 1*		**BACE2**	*beta-site APP-cleaving enzyme 2*	
**AP2B1**	*adaptor-related protein complex 2, beta 1 subunit*		**FLJ45079**		

**ASH1L**	*ash1 (absent, small, or homeotic)-like (Drosophila)*	✓	**NUP210**	*nucleoporin 210kDa*	✓
**TSPYL2**	*TSPY-like 2*		**UBR3**	*ubiquitin protein ligase E3 component n-recognin 3 (putative)*	✓
**C17orf59**	*chromosome 17 open reading frame 59*		**TRIM66**	*tripartite motif containing 66*	
**ABL2**	*c-abl oncogene 2, non-receptor tyrosine kinase*	✓	**LRRC58**	*leucine rich repeat containing 58*	
**ZNF362**	*zinc finger protein 362*		**DHTKD1**	*dehydrogenase E1 and transketolase domain containing 1*	
**PTHLH**	*parathyroid hormone-like hormone*		**NFATC2**	*nuclear factor of activated T-cells, cytoplasmic, calcineurin-dependent 2*	
**SLC10A7**	*solute carrier family 10, member 7*		**PLXNB1**	*plexin B1*	
**GPATCH8**	*G patch domain containing 8*	✓	**DSEL**	*dermatan sulfate epimerase-like*	
**GOLGA1**	*golgin A1*		**USP12**	*ubiquitin specific peptidase 12*	
**GLS2**	*glutaminase 2 (liver, mitochondrial)*		**XKR4**	*XK, Kell blood group complex subunit-related family, member 4*	
**FKBP1A**	*FK506 binding protein 1A, 12kDa*	✓	**PLEKHH1**	*pleckstrin homology domain containing, family H (with MyTH4 domain) member 1*	
**PLAGL1**	*pleiomorphic adenoma gene-like 1*		**STXBP1**	*syntaxin binding protein 1*	✓
**FSD1**	*fibronectin type III and SPRY domain containing 1*		**MBTPS2**	*membrane-bound transcription factor peptidase, site 2*	
**CHP1**	*calcineurin-like EF-hand protein 1*		**AATK**	*apoptosis-associated tyrosine kinase*	
**FKBP1C**	*FK506 binding protein 1C*		**ACTR1A**	*ARP1 actin-related protein 1 homolog A, centractin alpha (yeast)*	
**FAM110C**	*family with sequence similarity 110, member C*		**BCR**	*breakpoint cluster region*	
**AC013269.5**	*Uncharacterized protein*		**SKIL**	*SKI-like oncogene*	
**PDCD1**	*programmed cell death 1*	✓	**CLUH**	*clustered mitochondria (cluA/CLU1) homolog*	
**CLDN2**	*claudin 2*		**SEMA3D**	*sema domain, immunoglobulin domain (Ig), short basic domain, secreted, (semaphorin) 3D*	
**HPCAL4**	*hippocalcin like 4*		**DAB1**	*Dab, reelin signal transducer, homolog 1 (Drosophila)*	
**CXCL10**	*chemokine (C-X-C motif) ligand 10*		**FAM199X**	*family with sequence similarity 199, X-linked*	
**RBBP6**	*retinoblastoma binding protein 6*	✓	**SLC6A11**	*solute carrier family 6 (neurotransmitter transporter), member 11*	
**STX17**	*syntaxin 17*	✓	**SLC43A1**	*solute carrier family 43 (amino acid system L transporter), member 1*	

**KANK1**	*KN motif and ankyrin repeat domains 1*	✓	**FHDC1**	*FH2 domain containing 1*	
**NAA15**	*N(alpha)-acetyltransferase 15, NatA auxiliary subunit*		**FAM122B**	*family with sequence similarity 122B*	✓
**NRP2**	*neuropilin 2*		**UBAP1**	*ubiquitin associated protein 1*	
**ZBTB44**	*zinc finger and BTB domain containing 44*		**ENTPD5**	*ectonucleoside triphosphate diphosphohydrolase 5*	
**PPAP2B**	*phosphatidic acid phosphatase type 2B*	✓	**BSDC1**	*BSD domain containing 1*	
**RSBN1**	*round spermatid basic protein 1*		**UNC5B**	*unc-5 homolog B (C. elegans)*	
**RNF183**	*ring finger protein 183*		**TMEM259**	*transmembrane protein 259*	
**E2F3**	*E2F transcription factor 3*	✓	**AHCYL2**	*adenosylhomocysteinase-like 2*	
**CCDC178**	*coiled-coil domain containing 178*		**CSRNP2**	*cysteine-serine-rich nuclear protein 2*	
**PPIF**	*peptidylprolyl isomerase F*		**RLIM**	*ring finger protein, LIM domain interacting*	
**ACVR2A**	*activin A receptor, type IIA*	✓	**KIAA1549**	*KIAA1549*	
**TMED8**	*transmembrane emp24 protein transport domain containing 8*		**CHIC1**	*cysteine-rich hydrophobic domain 1*	
**LURAP1L**	*leucine rich adaptor protein 1-like*	✓	**FNIP2**	*folliculin interacting protein 2*	
**EXOC3L2**	*exocyst complex component 3-like 2*		**OXA1L**	*oxidase (cytochrome c) assembly 1-like*	
**FAM189B**	*family with sequence similarity 189, member B*		**LRRC15**	*leucine rich repeat containing 15*	
**KLHDC8B**	*kelch domain containing 8B*		**ADNP2**	*ADNP homeobox 2*	
**ARMCX2**	*armadillo repeat containing, X-linked 2*		**EYA4**	*eyes absent homolog 4 (Drosophila)*	
**ANLN**	*anillin, actin binding protein*		**CHRM3**	*cholinergic receptor, muscarinic 3*	
**DCUN1D1**	*DCN1, defective in cullin neddylation 1, domain containing 1*		**ELK4**	*ELK4, ETS-domain protein (SRF accessory protein 1)*	✓
**CLCN4**	*chloride channel, voltage-sensitive 4*		**RIMS3**	*regulating synaptic membrane exocytosis 3*	✓
**FAM135A**	*family with sequence similarity 135, member A*		**PIM3**	*pim-3 oncogene*	
**GNAQ**	*guanine nucleotide binding protein (G protein), q polypeptide*		**PHLPP2**	*PH domain and leucine rich repeat protein phosphatase 2*	✓
**PDIA6**	*protein disulfide isomerase family A, member 6*	✓	**AC174470.1**		
**AVL9**	*AVL9 homolog (S. cerevisiase)*	✓	**LRRFIP2**	*leucine rich repeat (in FLII) interacting protein 2*	✓
**ACACB**	*acetyl-CoA carboxylase beta*		**KCTD1**	*potassium channel tetramerization domain containing 1*	

**RORB**	*RAR-related orphan receptor B*		**RAB11FIP5**	*RAB11 family interacting protein 5 (class I)*	
**RAP2C**	*RAP2C, member of RAS oncogene family*	✓	**ROCK1**	*Rho-associated, coiled-coil containing protein kinase 1*	
**CAPRIN1**	*cell cycle associated protein 1*		**SLC4A7**	*solute carrier family 4, sodium bicarbonate cotransporter, member 7*	
**HOXA10**	*homeobox A10*	✓	**GPR153**	*G protein-coupled receptor 153*	
**BCL7A**	*B-cell CLL/lymphoma 7A*	✓	**STRN**	*striatin, calmodulin binding protein*	
**SVEP1**	*sushi, von Willebrand factor type A, EGF and pentraxin domain containing 1*		**OTUD4**	*OTU domain containing 4*	
**PHACTR4**	*phosphatase and actin regulator 4*		**SCUBE3**	*signal peptide, CUB domain, EGF-like 3*	
**CEP55**	*centrosomal protein 55kDa*	✓	**MKX**	*mohawk homeobox*	✓
**TAB3**	*TGF-beta activated kinase 1/MAP3K7 binding protein 3*		**CRAMP1L**	*Crm, cramped-like (Drosophila)*	
**ARL10**	*ADP-ribosylation factor-like 10*		**VTI1A**	*vesicle transport through interaction with t-SNAREs 1A*	
**IRAK2**	*interleukin-1 receptor-associated kinase 2*		**NFRKB**	*nuclear factor related to kappaB binding protein*	
**ELMOD1**	*ELMO/CED-12 domain containing 1*		**ZFYVE27**	*zinc finger, FYVE domain containing 27*	
**SLC36A1**	*solute carrier family 36 (proton/amino acid symporter), member 1*		**MYO5A**	*myosin VA (heavy chain 12, myoxin)*	✓
**USP19**	*ubiquitin specific peptidase 19*		**YIPF6**	*Yip1 domain family, member 6*	✓
**SCN3A**	*sodium channel, voltage-gated, type III, alpha subunit*		**LHPP**	*phospholysine phosphohistidine inorganic pyrophosphate phosphatase*	
**KLF7**	*Kruppel-like factor 7 (ubiquitous)*		**KCNK7**	*potassium channel, subfamily K, member 7*	
**FNTA**	*farnesyltransferase, CAAX box, alpha*		**ZNF704**	*zinc finger protein 704*	✓
**CLSPN**	*claspin*	✓	**ONECUT2**	*one cut homeobox 2*	
**NISCH**	*nischarin*		**CHD5**	*chromodomain helicase DNA binding protein 5*	
**DPY19L4**	*dpy-19-like 4 (C. elegans)*		**PPM1L**	*protein phosphatase, Mg2+/Mn2+ dependent, 1L*	
**ZBTB43**	*zinc finger and BTB domain containing 43*		**ZC3H12B**	*zinc finger CCCH-type containing 12B*	
**ARPP21**	*cAMP-regulated phosphoprotein, 21kDa*		**KLC2**	*kinesin light chain 2*	✓
**C8orf58**	*chromosome 8 open reading frame 58*		**FAM134C**	*family with sequence similarity 134, member C*	
**TMEM87B**	*transmembrane protein 87B*		**UBXN10**	*UBX domain protein 10*	

**APP**	*amyloid beta (A4) precursor protein*	✓	**ZNF397**	*zinc finger protein 397*	
**RNF43**	*ring finger protein 43*		**GNA13**	*guanine nucleotide binding protein (G protein), alpha 13*	
**TCAIM**	*T cell activation inhibitor, mitochondrial*		**PODXL**	*podocalyxin-like*	
**LARP1B**	*La ribonucleoprotein domain family, member 1B*		**FAF1**	*Fas (TNFRSF6) associated factor 1*	
**BCL2**	*B-cell CLL/lymphoma 2*	✓	**PPIG**	*peptidylprolyl isomerase G (cyclophilin G)*	✓
**CC2D1B**	*coiled-coil and C2 domain containing 1B*		**AVEN**	*apoptosis, caspase activation inhibitor*	
**ZBTB39**	*zinc finger and BTB domain containing 39*		**DIAPH1**	*diaphanous-related formin 1*	
**GALNT1**	*UDP-N-acetyl-alpha-D-galactosamine:polypeptide N-acetylgalactosaminyltransferase 1 (GalNAc-T1)*	✓	**RBM24**	*RNA binding motif protein 24*	
**ZC3H11A**	*zinc finger CCCH-type containing 11A*		**HMGXB4**	*HMG box domain containing 4*	
**PDIK1L**	*PDLIM1 interacting kinase 1 like*	✓	**MINK1**	*misshapen-like kinase 1*	✓
**BMPR1A**	*bone morphogenetic protein receptor, type IA*		**GSE1**	*Gse1 coiled-coil protein*	
**ZNF275**	*zinc finger protein 275*	✓	**NYNRIN**	*NYN domain and retroviral integrase containing*	
**FAM73B**	*family with sequence similarity 73, member B*		**SHROOM3**	*shroom family member 3*	
**PVRL1**	*poliovirus receptor-related 1 (herpesvirus entry mediator C)*		**PKDCC**	*protein kinase domain containing, cytoplasmic*	
**TMEM178B**	*transmembrane protein 178B*		**UNC13A**	*unc-13 homolog A (C. elegans)*	
**PRR15L**	*proline rich 15-like*		**RAB10**	*RAB10, member RAS oncogene family*	
**YAP1**	*Yes-associated protein 1*		**ABI2**	*abl-interactor 2*	
**BICD1**	*bicaudal D homolog 1 (Drosophila)*		**GAS2L3**	*growth arrest-specific 2 like 3*	
**PRKAB2**	*protein kinase, AMP-activated, beta 2 non-catalytic subunit*		**NAPEPLD**	*N-acyl phosphatidylethanolamine phospholipase D*	
**CNIH3**	*cornichon family AMPA receptor auxiliary protein 3*		**SEC31B**	*SEC31 homolog B (S. cerevisiae)*	
**COPS2**	*COP9 signalosome subunit 2*		**DPP8**	*dipeptidyl-peptidase 8*	✓
**AP2A1**	*adaptor-related protein complex 2, alpha 1 subunit*		**PAK7**	*p21 protein (Cdc42/Rac)-activated kinase 7*	
**NEBL**	*nebulette*		**STK32A**	*serine/threonine kinase 32A*	
**LATS2**	*large tumor suppressor kinase 2*		**TRAM1**	*translocation associated membrane protein 1*	✓

**ARFGAP2**	*ADP-ribosylation factor GTPase activating protein 2*		**TNPO1**	*transportin 1*	
**PMM1**	*phosphomannomutase 1*		**ZNF629**	*zinc finger protein 629*	
**NCS1**	*neuronal calcium sensor 1*		**CACNA1E**	*calcium channel, voltage-dependent, R type, alpha 1E subunit*	
**AEBP2**	*AE binding protein 2*		**PI4KB**	*phosphatidylinositol 4-kinase, catalytic, beta*	
**RASEF**	*RAS and EF-hand domain containing*	✓	**POU3F2**	*POU class 3 homeobox 2*	
**ARHGDIA**	*Rho GDP dissociation inhibitor (GDI) alpha*	✓	**WDR47**	*WD repeat domain 47*	
**CUL2**	*cullin 2*	✓	**GLCE**	*glucuronic acid epimerase*	
**ST8SIA3**	*ST8 alpha-N-acetyl-neuraminide alpha-2,8-sialyltransferase 3*		**USP31**	*ubiquitin specific peptidase 31*	✓
**GRAMD3**	*GRAM domain containing 3*	✓	**NAA25**	*N(alpha)-acetyltransferase 25, NatB auxiliary subunit*	✓
**HPSE2**	*heparanase 2*		**RAD51L3-RFFL**	*Uncharacterized protein*	
**SLC15A4**	*solute carrier family 15 (oligopeptide transporter), member 4*		**HCN1**	*hyperpolarization activated cyclic nucleotide-gated potassium channel 1*	
**LIPE**	*lipase, hormone-sensitive*		**LRP2**	*low density lipoprotein receptor-related protein 2*	
**CSRNP1**	*cysteine-serine-rich nuclear protein 1*		**VTA1**	*vesicle (multivesicular body) trafficking 1*	
**PAG1**	*phosphoprotein associated with glycosphingolipid microdomains 1*	✓	**LIN28A**	*lin-28 homolog A (C. elegans)*	
**OIP5**	*Opa interacting protein 5*		**PHKA1**	*phosphorylase kinase, alpha 1 (muscle)*	✓
**POU2F1**	*POU class 2 homeobox 1*	✓	**ZNF469**	*zinc finger protein 469*	
**BTN1A1**	*butyrophilin, subfamily 1, member A1*		**RAB37**	*RAB37, member RAS oncogene family*	
**ABCG4**	*ATP-binding cassette, sub-family G (WHITE), member 4*		**EDNRA**	*endothelin receptor type A*	
**MAP4**	*microtubule-associated protein 4*		**ARHGAP20**	*Rho GTPase activating protein 20*	
**ATXN7L3B**	*ataxin 7-like 3B*	✓	**GLUD1**	*glutamate dehydrogenase 1*	
**RAF1**	*v-raf-1 murine leukemia viral oncogene homolog 1*		**PI4K2A**	*Phosphatidylinositol 4-kinase type 2-alpha; Uncharacterized protein*	
**WNT4**	*wingless-type MMTV integration site family, member 4*		**TRIP10**	*thyroid hormone receptor interactor 10*	
**AMMECR1L**	*AMMECR1-like*	✓	**DCAF8**	*DDB1- and CUL4-associated factor 8*	

**SYT10**	*synaptotagmin X*		**SLC2A13**	*solute carrier family 2 (facilitated glucose transporter), member 13*	
**CTDSP2**	*CTD (carboxy-terminal domain, RNA polymerase II, polypeptide A) small phosphatase 2*		**FKBP5**	*FK506 binding protein 5*	
**UHMK1**	*U2AF homology motif (UHM) kinase 1*		**TSPAN9**	*tetraspanin 9*	
**PCDHA7**	*protocadherin alpha 7*		**JMJD1C**	*jumonji domain containing 1C*	
**PCDHA5**	*protocadherin alpha 5*		**CD2AP**	*CD2-associated protein*	✓
**PCDHA12**	*protocadherin alpha 12*		**TBC1D9**	*TBC1 domain family, member 9 (with GRAM domain)*	
**G2E3**	*G2/M-phase specific E3 ubiquitin protein ligase*		**FCHSD2**	*FCH and double SH3 domains 2*	
**ANKS1A**	*ankyrin repeat and sterile alpha motif domain containing 1A*		**JOSD1**	*Josephin domain containing 1*	
**EIF4B**	*eukaryotic translation initiation factor 4B*		**MAPK1IP1L**	*mitogen-activated protein kinase 1 interacting protein 1-like*	
**PPM1H**	*protein phosphatase, Mg2+/Mn2+ dependent, 1H*		**CBX5**	*chromobox homolog 5*	
**PRDM4**	*PR domain containing 4*	✓	**KATNAL1**	*katanin p60 subunit A-like 1*	✓
**PCDHA8**	*protocadherin alpha 8*		**SEMA3A**	*sema domain, immunoglobulin domain (Ig), short basic domain, secreted, (semaphorin) 3A*	
**PCDHA2**	*protocadherin alpha 2*		**OGT**	*O-linked N-acetylglucosamine (GlcNAc) transferase*	✓
**PCDHA13**	*protocadherin alpha 13*		**FANCB**	*Fanconi anemia, complementation group B*	
**PCDHA4**	*protocadherin alpha 4*		**PPM1K**	*protein phosphatase, Mg2+/Mn2+ dependent, 1K*	
**PCDHA9**	*protocadherin alpha 9*		**RFFL**	*ring finger and FYVE-like domain containing E3 ubiquitin protein ligase*	
**PCDHA6**	*protocadherin alpha 6*		**PSME3**	*proteasome (prosome, macropain) activator subunit 3 (PA28 gamma; Ki)*	
**PCDHA1**	*protocadherin alpha 1*		**DHDDS**	*dehydrodolichyl diphosphate synthase*	
**PCDHA10**	*protocadherin alpha 10*		**TERT**	*telomerase reverse transcriptase*	
**TAF15**	*TAF15 RNA polymerase II, TATA box binding protein (TBP)-associated factor, 68kDa*		**EIF5A2**	*eukaryotic translation initiation factor 5A2*	
**FOSL1**	*FOS-like antigen 1*		**STXBP5**	*syntaxin binding protein 5 (tomosyn)*	
**ATXN1L**	*ataxin 1-like*		**SPEN**	*spen homolog, transcriptional regulator (Drosophila)*	
**PCDHAC1**	*protocadherin alpha subfamily C, 1*		**CERS1**	*ceramide synthase 1*	

**CCDC42B**	*coiled-coil domain containing 42B*		**ZER1**	*zyg-11 related, cell cycle regulator*	
**CPD**	*carboxypeptidase D*		**DDX3X**	*DEAD (Asp-Glu-Ala-Asp) box helicase 3, X-linked*	✓
**GATAD2A**	*GATA zinc finger domain containing 2A*		**GOLT1B**	*golgi transport 1B*	
**TSPAN5**	*tetraspanin 5*		**PCDH1**	*protocadherin 1*	
**PPP6C**	*protein phosphatase 6, catalytic subunit*	✓	**FBXO10**	*F-box protein 10*	
**CD163**	*CD163 molecule*		**KCNC4**	*potassium voltage-gated channel, Shaw-related subfamily, member 4*	
**ZC2HC1A**	*zinc finger, C2HC-type containing 1A*		**FUBP1**	*far upstream element (FUSE) binding protein 1*	
**CASK**	*calcium/calmodulin-dependent serine protein kinase (MAGUK family)*	✓	**PITPNM3**	*PITPNM family member 3*	
**YTHDC1**	*YTH domain containing 1*	✓	**DLGAP2**	*discs, large (Drosophila) homolog-associated protein 2*	
**VMA21**	*VMA21 vacuolar H+-ATPase homolog (S. cerevisiae)*		**CDCA2**	*cell division cycle associated 2*	
**PPP2R5C**	*protein phosphatase 2, regulatory subunit B′, gamma*	✓	**ATG14**	*autophagy related 14*	✓
**LCOR**	*ligand dependent nuclear receptor corepressor*		**KCNE4**	*potassium voltage-gated channel, Isk-related family, member 4*	
**SPSB4**	*splA/ryanodine receptor domain and SOCS box containing 4*		**DENND2C**	*DENN/MADD domain containing 2C*	
**CAMKV**	*CaM kinase-like vesicle-associated*		**TMEM38A**	*transmembrane protein 38A*	
**CREBRF**	*CREB3 regulatory factor*	✓	**SGSM2**	*small G protein signaling modulator 2*	
**PISD**	*phosphatidylserine decarboxylase*	✓	**MARCH9**	*membrane-associated ring finger (C3HC4) 9*	
**DCAF7**	*DDB1 and CUL4 associated factor 7*		**TXNIP**	*thioredoxin interacting protein*	
**GPN1**	*GPN-loop GTPase 1*		**LONRF3**	*LON peptidase N-terminal domain and ring finger 3*	
**CBLC**	*Cbl proto-oncogene C, E3 ubiquitin protein ligase*		**ARHGAP35**	*Rho GTPase activating protein 35*	
**JARID2**	*jumonji, AT rich interactive domain 2*	✓	**CDK12**	*cyclin-dependent kinase 12*	
**RAPGEF2**	*Rap guanine nucleotide exchange factor (GEF) 2*		**MCF2L**	*MCF.2 cell line derived transforming sequence-like*	
**EFNB2**	*ephrin-B2*	✓	**TADA2B**	*transcriptional adaptor 2B*	✓
**VAMP8**	*vesicle-associated membrane protein 8*		**MMP24**	*matrix metallopeptidase 24 (membrane-inserted)*	

**NUP50**	*nucleoporin 50kDa*	✓	**ZNF831**	*zinc finger protein 831*	
**UBFD1**	*ubiquitin family domain containing 1*		**ESR2**	*estrogen receptor 2 (ER beta)*	
**YWHAQ**	*tyrosine 3-monooxygenase/tryptophan 5-monooxygenase activation protein, theta polypeptide*	✓	**NPR3**	*natriuretic peptide receptor C/guanylate cyclase C (atrionatriuretic peptide receptor C)*	
**IGF1R**	*insulin-like growth factor 1 receptor*		**CDX2**	*caudal type homeobox 2*	
**SPTLC1**	*serine palmitoyltransferase, long chain base subunit 1*	✓	**MED1**	*mediator complex subunit 1*	
**ZSWIM3**	*zinc finger, SWIM-type containing 3*		**PPP2R1B**	*protein phosphatase 2, regulatory subunit A, beta*	
**C20orf194**	*chromosome 20 open reading frame 194*		**ALX4**	*ALX homeobox 4*	
**PRKAR2A**	*protein kinase, cAMP-dependent, regulatory, type II, alpha*	✓	**VWA7**	*von Willebrand factor A domain containing 7*	
**EIF3M**	*eukaryotic translation initiation factor 3, subunit M*		**TUBA4A**	*tubulin, alpha 4a*	
**CBFA2T3**	*core-binding factor, runt domain, alpha subunit 2; translocated to, 3*	✓	**VPS13B**	*vacuolar protein sorting 13 homolog B (yeast)*	
**SSR1**	*signal sequence receptor, alpha*		**WDFY2**	*WD repeat and FYVE domain containing 2*	
**RASSF4**	*Ras association (RalGDS/AF-6) domain family member 4*		**TET3**	*tet methylcytosine dioxygenase 3*	✓
**FAM101B**	*family with sequence similarity 101, member B*		**SOX6**	*SRY (sex determining region Y)-box 6*	
**CTNNBIP1**	*catenin, beta interacting protein 1*		**GIGYF1**	*GRB10 interacting GYF protein 1*	
**SLC4A4**	*solute carrier family 4 (sodium bicarbonate cotransporter), member 4*		**MGAT4A**	*mannosyl (alpha-1,3-)-glycoprotein beta-1,4-N-acetylglucosaminyltransferase, isozyme A*	
**SCN1A**	*sodium channel, voltage-gated, type I, alpha subunit*		**KREMEN2**	*kringle containing transmembrane protein 2*	
**CMPK1**	*cytidine monophosphate (UMP-CMP) kinase 1, cytosolic*		**SUPT16H**	*suppressor of Ty 16 homolog (S. cerevisiae)*	✓
**EGLN2**	*egl-9 family hypoxia-inducible factor 2*		**CAB39**	*calcium binding protein 39*	
**DNAJC5**	*DnaJ (Hsp40) homolog, subfamily C, member 5*	✓	**TBX18**	*T-box 18*	
**MPHOSPH9**	*M-phase phosphoprotein 9*	✓	**TCF3**	*transcription factor 3*	✓
**ZHX1**	*zinc fingers and homeoboxes 1*		**TM7SF3**	*transmembrane 7 superfamily member 3*	✓
**NRBP1**	*nuclear receptor binding protein 1*		**B3GALT1**	*UDP-Gal:betaGlcNAc beta 1,3-galactosyltransferase, polypeptide 1*	

**DNAJA2**	*DnaJ (Hsp40) homolog, subfamily A, member 2*		**IL17RE**	*interleukin 17 receptor E*	
**PI4K2B**	*phosphatidylinositol 4-kinase type 2 beta*	✓	**PABPC1L**	*poly(A) binding protein, cytoplasmic 1-like*	
**CHD2**	*chromodomain helicase DNA binding protein 2*		**SREK1**	*splicing regulatory glutamine/lysine-rich protein 1*	✓
**ENAH**	*enabled homolog (Drosophila)*		**STYX**	*serine/threonine/tyrosine interacting protein*	
**KCNQ5**	*potassium voltage-gated channel, KQT-like subfamily, member 5*		**ARHGAP26**	*Rho GTPase activating protein 26*	
**EGLN1**	*egl-9 family hypoxia-inducible factor 1*		**PSMA5**	*proteasome (prosome, macropain) subunit, alpha type, 5*	
**CHPT1**	*choline phosphotransferase 1*		**C10orf76**	*chromosome 10 open reading frame 76*	
**SH2D2A**	*SH2 domain containing 2A*		**SNX1**	*sorting nexin 1*	
**ADRB2**	*adrenoceptor beta 2, surface*		**SPATC1**	*spermatogenesis and centriole associated 1*	
**FICD**	*FIC domain containing*		**HECTD4**	*HECT domain containing E3 ubiquitin protein ligase 4*	
**ZBTB33**	*zinc finger and BTB domain containing 33*	✓	**WDR37**	*WD repeat domain 37*	
**FZD6**	*frizzled family receptor 6*	✓	**DCAF5**	*DDB1 and CUL4 associated factor 5*	
**LRIG2**	*leucine-rich repeats and immunoglobulin-like domains 2*	✓	**SPTBN2**	*spectrin, beta, non-erythrocytic 2*	
**B4GALT1**	*UDP-Gal:betaGlcNAc beta 1,4-galactosyltransferase, polypeptide 1*	✓	**SGMS2**	*sphingomyelin synthase 2*	
**CLOCK**	*clock circadian regulator*		**CHRNE**	*cholinergic receptor, nicotinic, epsilon (muscle)*	
**CALU**	*calumenin*	✓	**CDS2**	*CDP-diacylglycerol synthase (phosphatidate cytidylyltransferase) 2*	✓
**PNISR**	*PNN-interacting serine/arginine-rich protein*	✓	**BCL9L**	*B-cell CLL/lymphoma 9-like*	
**TSC22D2**	*TSC22 domain family, member 2*	✓	**TRPM3**	*transient receptor potential cation channel, subfamily M, member 3*	
**LRIG1**	*leucine-rich repeats and immunoglobulin-like domains 1*		**SLC4A8**	*solute carrier family 4, sodium bicarbonate cotransporter, member 8*	
**NAT8L**	*N-acetyltransferase 8-like (GCN5-related, putative)*		**PDZD8**	*PDZ domain containing 8*	
**TXN2**	*thioredoxin 2*		**TYRO3**	*TYRO3 protein tyrosine kinase*	
**SLC39A9**	*solute carrier family 39, member 9*	✓	**SLC5A3**	*sodium/myo-inositol cotransporter*	
**SOWAHC**	*sosondowah ankyrin repeat domain family member C*	✓	**ANKRD33B**	*ankyrin repeat domain 33B*	

**CCDC85C**	*coiled-coil domain containing 85C*		**PPAN-P2RY11**	*PPAN-P2RY11 readthrough*	
**ZC3H6**	*zinc finger CCCH-type containing 6*		**EPB41L1**	*erythrocyte membrane protein band 4.1-like 1*	
**SOCS5**	*suppressor of cytokine signaling 5*	✓	**A1CF**	*APOBEC1 complementation factor*	
**SIAH1**	*siah E3 ubiquitin protein ligase 1*		**NLGN1**	*neuroligin 1*	
**NPAS3**	*neuronal PAS domain protein 3*		**AKAP11**	*A kinase (PRKA) anchor protein 11*	✓
**SSTR3**	*somatostatin receptor 3*		**COG3**	*component of oligomeric golgi complex 3*	
**USP6NL**	*USP6 N-terminal like*		**GPN2**	*GPN-loop GTPase 2*	
**RREB1**	*ras responsive element binding protein 1*		**VAV2**	*vav 2 guanine nucleotide exchange factor*	✓
**BAG5**	*BCL2-associated athanogene 5*		**CAST**	*calpastatin*	
**C11orf68**	*chromosome 11 open reading frame 68*		**ZNRF2**	*zinc and ring finger 2*	✓
**MAP2K3**	*mitogen-activated protein kinase kinase 3*		**IGF1**	*insulin-like growth factor 1 (somatomedin C)*	
**LAMTOR3**	*late endosomal/lysosomal adaptor, MAPK and MTOR activator 3*		**SIX5**	*SIX homeobox 5*	
**WIF1**	*WNT inhibitory factor 1*		**IPO9**	*importin 9*	
**TGFBR3**	*transforming growth factor, beta receptor III*	✓	**MYEF2**	*myelin expression factor 2*	
**PANK1**	*pantothenate kinase 1*	✓	**BRI3BP**	*BRI3 binding protein*	
**IL2RB**	*interleukin 2 receptor, beta*		**LDLRAD4**	*low density lipoprotein receptor class A domain containing 4*	
**ITGA2**	*integrin, alpha 2 (CD49B, alpha 2 subunit of VLA-2 receptor)*	✓	**AGPAT3**	*1-acylglycerol-3-phosphate O-acyltransferase 3*	
**SYNDIG1**	*synapse differentiation inducing 1*		**DRP2**	*dystrophin related protein 2*	
**BACE1**	*beta-site APP-cleaving enzyme 1*		**CD180**	*CD180 molecule*	✓
**KLHL15**	*kelch-like family member 15*	✓	**PLXNA4**	*plexin A4*	
**LRP6**	*low density lipoprotein receptor-related protein 6*		**SLC9A3R2**	*solute carrier family 9, subfamily A (NHE3, cation proton antiporter 3), member 3 regulator 2*	
**NAPG**	*N-ethylmaleimide-sensitive factor attachment protein, gamma*	✓	**TXLNG**	*taxilin gamma*	
**CRIM1**	*cysteine rich transmembrane BMP regulator 1 (chordin-like)*	✓	**FADS6**	*fatty acid desaturase 6*	
**SNX11**	*sorting nexin 11*	✓	**KMT2C**	*lysine (K)-specific methyltransferase 2C*	✓

**DCUN1D4**	*DCN1, defective in cullin neddylation 1, domain containing 4*		**TSC1**	*tuberous sclerosis 1*	
**PNRC2**	*proline-rich nuclear receptor coactivator 2*	✓	**PITPNA**	*phosphatidylinositol transfer protein, alpha*	
**ITPRIPL1**	*inositol 1,4,5-trisphosphate receptor interacting protein-like 1*		**NFASC**	*neurofascin*	
**ATP7A**	*ATPase, Cu++ transporting, alpha polypeptide*		**NUAK1**	*NUAK family, SNF1-like kinase, 1*	
**COBLL1**	*cordon-bleu WH2 repeat protein-like 1*		**ATXN7L2**	*ataxin 7-like 2*	
**LHX1**	*LIM homeobox 1*		**TMEM87A**	*transmembrane protein 87A*	
**SLC25A22**	*solute carrier family 25 (mitochondrial carrier: glutamate), member 22*	✓	**AC005003.1**	*CDNA FLJ20464 fis, clone KAT06158; HCG1777549; Uncharacterized protein*	
**PDPR**	*pyruvate dehydrogenase phosphatase regulatory subunit*		**MAP1A**	*microtubule-associated protein 1A*	
**COL24A1**	*collagen, type XXIV, alpha 1*		**ARMC5**	*armadillo repeat containing 5*	
**DCTN5**	*dynactin 5 (p25)*	✓	**NFIC**	*nuclear factor I/C (CCAAT-binding transcription factor)*	✓
**RAB3D**	*RAB3D, member RAS oncogene family*		**KIAA0586**	*KIAA0586*	
**POLE4**	*polymerase (DNA-directed), epsilon 4, accessory subunit*	✓	**SPRTN**	*SprT-like N-terminal domain*	
**SMAD3**	*SMAD family member 3*	✓	**SETD6**	*SET domain containing 6*	
**SHROOM4**	*shroom family member 4*		**TRIM45**	*tripartite motif containing 45*	
**TCTE1**	*t-complex-associated-testis-expressed 1*		**TOMM20**	*translocase of outer mitochondrial membrane 20 homolog (yeast)*	
**MFN2**	*mitofusin 2*		**PLXNA1**	*plexin A1*	
**SEPT11**	*septin 11*		**UBE2B**	*ubiquitin-conjugating enzyme E2B*	
**PPFIA2**	*protein tyrosine phosphatase, receptor type, f polypeptide (PTPRF), interacting protein (liprin), alpha 2*		**USP15**	*ubiquitin specific peptidase 15*	✓
**NBR1**	*neighbor of BRCA1 gene 1*		**MT1A**	*metallothionein 1A*	
**ATF6**	*activating transcription factor 6*		**METTL21B**	*methyltransferase like 21B*	
**NKD1**	*naked cuticle homolog 1 (Drosophila)*		**ETNK1**	*ethanolamine kinase 1*	✓
**EED**	*embryonic ectoderm development*		**SRPK1**	*SRSF protein kinase 1*	✓
**FOXK1**	*forkhead box K1*	✓	**PHC3**	*polyhomeotic homolog 3 (Drosophila)*	✓

**MOCS1**	*molybdenum cofactor synthesis 1*		**YOD1**	*YOD1 deubiquitinase*	
**MARCH5**	*membrane-associated ring finger (C3HC4) 5*		**EPB41L4B**	*erythrocyte membrane protein band 4.1 like 4B*	✓
**CRK**	*v-crk avian sarcoma virus CT10 oncogene homolog*	✓	**CYLD**	*cylindromatosis (turban tumor syndrome)*	✓
**HOXC8**	*homeobox C8*	✓	**PPIL4**	*peptidylprolyl isomerase (cyclophilin)-like 4*	
**MT2A**	*metallothionein 2A*		**MFRP**	*membrane frizzled-related protein*	
**DNAJC16**	*DnaJ (Hsp40) homolog, subfamily C, member 16*		**PGM2L1**	*phosphoglucomutase 2-like 1*	
**PAFAH1B2**	*platelet-activating factor acetylhydrolase 1b, catalytic subunit 2 (30kDa)*	✓	**SESTD1**	*SEC14 and spectrin domains 1*	✓
**ZDHHC14**	*zinc finger, DHHC-type containing 14*		**HNRNPA2B1**	*heterogeneous nuclear ribonucleoprotein A2/B1*	✓
**RAB40A**	*RAB40A, member RAS oncogene family*		**ZNF652**	*zinc finger protein 652*	
**ESRRA**	*estrogen-related receptor alpha*		**NMNAT2**	*nicotinamide nucleotide adenylyltransferase 2*	
**MT1G**	*metallothionein 1G*		**RPRD2**	*regulation of nuclear pre-mRNA domain containing 2*	✓
**TRIP11**	*thyroid hormone receptor interactor 11*		**CDKN2B**	*cyclin-dependent kinase inhibitor 2B (p15, inhibits CDK4)*	
**SMAD5**	*SMAD family member 5*		**NUFIP2**	*nuclear fragile X mental retardation protein interacting protein 2*	✓
**DIXDC1**	*DIX domain containing 1*		**SLC20A2**	*solute carrier family 20 (phosphate transporter), member 2*	
**MBNL2**	*muscleblind-like splicing regulator 2*		**SYPL1**	*synaptophysin-like 1*	✓
**EMX1**	*empty spiracles homeobox 1*		**CTIF**	*CBP80/20-dependent translation initiation factor*	
**FAM60A**	*family with sequence similarity 60, member A*		**COLGALT2**	*collagen beta(1-O)galactosyltransferase 2*	
**PWWP2B**	*PWWP domain containing 2B*		**AP3M1**	*adaptor-related protein complex 3, mu 1 subunit*	✓
**PRPF38A**	*pre-mRNA processing factor 38A*		**ATP6V1B2**	*ATPase, H+ transporting, lysosomal 56/58kDa, V1 subunit B2*	
**CDC23**	*cell division cycle 23*		**P2RY2**	*purinergic receptor P2Y, G-protein coupled, 2*	
**SRSF11**	*serine/arginine-rich splicing factor 11*		**ING2**	*inhibitor of growth family, member 2*	
**SH3BGRL2**	*SH3 domain binding glutamic acid-rich protein like 2*		**M6PR**	*mannose-6-phosphate receptor (cation dependent)*	
**PLCD1**	*phospholipase C, delta 1*		**SUSD1**	*sushi domain containing 1*	
**TBP**	*TATA box binding protein*		**APC**	*adenomatous polyposis coli*	

**CHD7**	*chromodomain helicase DNA binding protein 7*		**EPC1**	*enhancer of polycomb homolog 1 (Drosophila)*	
**IRS1**	*insulin receptor substrate 1*		**DYRK2**	*dual-specificity tyrosine-(Y)-phosphorylation regulated kinase 2*	
**SNRK**	*SNF related kinase*		**SCAI**	*suppressor of cancer cell invasion*	
**SOX5**	*SRY (sex determining region Y)-box 5*		**CALM1**	*calmodulin 1 (phosphorylase kinase, delta)*	
**RAB4B**	*RAB4B, member RAS oncogene family*		**TRIM72**	*tripartite motif containing 72*	
**LPP**	*LIM domain containing preferred translocation partner in lipoma*		**CNKSR3**	*CNKSR family member 3*	✓
**HECTD1**	*HECT domain containing E3 ubiquitin protein ligase 1*		**SUZ12**	*SUZ12 polycomb repressive complex 2 subunit*	
**HAS2**	*hyaluronan synthase 2*		**DICER1**	*dicer 1, ribonuclease type III*	✓
**EZH1**	*enhancer of zeste homolog 1 (Drosophila)*	✓	**RNF213**	*ring finger protein 213*	
**FAM63B**	*family with sequence similarity 63, member B*		**KMT2A**	*lysine (K)-specific methyltransferase 2A*	
**SIDT2**	*SID1 transmembrane family, member 2*	✓	**ROCK2**	*Rho-associated, coiled-coil containing protein kinase 2*	
**EIF2B5**	*eukaryotic translation initiation factor 2B, subunit 5 epsilon, 82kDa*		**ARMC8**	*armadillo repeat containing 8*	
**TVP23B**	*trans-golgi network vesicle protein 23 homolog B (S. cerevisiae)*		**ZHX3**	*zinc fingers and homeoboxes 3*	
**GPSM3**	*G-protein signaling modulator 3*		**ARHGAP42**	*Rho GTPase activating protein 42*	
**GAN**	*gigaxonin*		**NRARP**	*NOTCH-regulated ankyrin repeat protein*	
**PTGFR**	*prostaglandin F receptor (FP)*		**SMS**	*spermine synthase*	
**JPH1**	*junctophilin 1*		**ANKRD40**	*ankyrin repeat domain 40*	
**KBTBD2**	*kelch repeat and BTB (POZ) domain containing 2*		**DDX19B**	*DEAD (Asp-Glu-Ala-Asp) box polypeptide 19B*	
**CEP85L**	*centrosomal protein 85kDa-like*		**SIT1**	*signaling threshold regulating transmembrane adaptor 1*	
**ESRP1**	*epithelial splicing regulatory protein 1*		**PCM1**	*pericentriolar material 1*	
**HIPK2**	*homeodomain interacting protein kinase 2*		**ARHGEF39**	*Rho guanine nucleotide exchange factor (GEF) 39*	
**COL12A1**	*collagen, type XII, alpha 1*	✓	**BAZ2A**	*bromodomain adjacent to zinc finger domain, 2A*	✓
**SIPA1L2**	*signal-induced proliferation-associated 1 like 2*		**SRGAP1**	*SLIT-ROBO Rho GTPase activating protein 1*	
**SERBP1**	*SERPINE1 mRNA binding protein 1*	✓	**FGF1**	*fibroblast growth factor 1 (acidic)*	

**MLK4**	*Mitogen-activated protein kinase kinase kinase MLK4*		**LHFPL4**	*lipoma HMGIC fusion partner-like 4*	
**CCDC28A**	*coiled-coil domain containing 28A*		**NPY4R**	*neuropeptide Y receptor Y4*	
**TRABD2B**	*TraB domain containing 2B*		**PIAS1**	*protein inhibitor of activated STAT, 1*	
**TBC1D19**	*TBC1 domain family, member 19*		**PELP1**	*proline, glutamate and leucine rich protein 1*	
**KCTD8**	*potassium channel tetramerization domain containing 8*		**MRAS**	*muscle RAS oncogene homolog*	
**KIAA0319L**	*KIAA0319-like*		**SESN2**	*sestrin 2*	
**RET**	*ret proto-oncogene*		**PHIP**	*pleckstrin homology domain interacting protein*	
**KHNYN**	*KH and NYN domain containing*		**GRIN1**	*glutamate receptor, ionotropic, N-methyl D-aspartate 1*	
**RRAGA**	*Ras-related GTP binding A*		**PRELP**	*proline/arginine-rich end leucine-rich repeat protein*	
**GTPBP1**	*GTP binding protein 1*		**STARD9**	*StAR-related lipid transfer (START) domain containing 9*	
**TMEM154**	*transmembrane protein 154*		**AL391421.1**	*Uncharacterized protein; cDNA FLJ43696 fis, clone TBAES2007964*	
**MARCH4**	*membrane-associated ring finger (C3HC4) 4, E3 ubiquitin protein ligase*		**LTB**	*lymphotoxin beta (TNF superfamily, member 3)*	
**USP44**	*ubiquitin specific peptidase 44*		**DKFZP761J1410**	*Lipid phosphate phosphatase-related protein type 2*	
**PIAS2**	*protein inhibitor of activated STAT, 2*		**AC137932.1**		
**MTMR3**	*myotubularin related protein 3*	✓	**FASTK**	*Fas-activated serine/threonine kinase*	
**ITGA10**	*integrin, alpha 10*		**RUNX1**	*runt-related transcription factor 1*	
**HDGF**	*hepatoma-derived growth factor*	✓	**NXPE3**	*neurexophilin and PC-esterase domain family, member 3*	
**PDCD6IP**	*programmed cell death 6 interacting protein*		**TIPIN**	*TIMELESS interacting protein*	
**RELT**	*RELT tumor necrosis factor receptor*	✓	**SBNO1**	*strawberry notch homolog 1 (Drosophila)*	✓
**CYGB**	*cytoglobin*		**ESF1**	*ESF1, nucleolar pre-rRNA processing protein, homolog (S. cerevisiae)*	
**AL163636.6**	*Homo sapiens ribonuclease, RNase A family, 4 (RNASE4), transcript variant 4, mRNA.*		**SRCAP**	*Snf2-related CREBBP activator protein*	
**AKIRIN1**	*akirin 1*		**CDC6**	*cell division cycle 6*	
**ZCCHC2**	*zinc finger, CCHC domain containing 2*		**CBX2**	*chromobox homolog 2*	✓

**SWAP70**	*SWAP switching B-cell complex 70kDa subunit*		**PPP1R3F**	*protein phosphatase 1, regulatory subunit 3F*	
**ZCCHC5**	*zinc finger, CCHC domain containing 5*		**GMPPB**	*GDP-mannose pyrophosphorylase B*	
**PDE3B**	*phosphodiesterase 3B, cGMP-inhibited*		**PHF21A**	*PHD finger protein 21A*	
**PRSS12**	*protease, serine, 12 (neurotrypsin, motopsin)*		**LIMS1**	*LIM and senescent cell antigen-like domains 1*	
**STK38**	*serine/threonine kinase 38*	✓	**TXNDC17**	*thioredoxin domain containing 17*	
**WNT10B**	*wingless-type MMTV integration site family, member 10B*		**UBR4**	*ubiquitin protein ligase E3 component n-recognin 4*	
**UBN2**	*ubinuclein 2*	✓	**RAB11FIP1**	*RAB11 family interacting protein 1 (class I)*	
**HNRNPA1**	*heterogeneous nuclear ribonucleoprotein A1*	✓	**POP4**	*processing of precursor 4, ribonuclease P/MRP subunit (S. cerevisiae)*	
**AGO1**	*argonaute RISC catalytic component 1*		**PPIP5K2**	*diphosphoinositol pentakisphosphate kinase 2*	✓
**NHLRC2**	*NHL repeat containing 2*		**C17orf85**	*chromosome 17 open reading frame 85*	
**AFF4**	*AF4/FMR2 family, member 4*	✓	**PAK2**	*p21 protein (Cdc42/Rac)-activated kinase 2*	✓
**PDK3**	*pyruvate dehydrogenase kinase, isozyme 3*		**STRBP**	*spermatid perinuclear RNA binding protein*	
**SLITRK1**	*SLIT and NTRK-like family, member 1*		**CCDC6**	*coiled-coil domain containing 6*	
**FAT4**	*FAT atypical cadherin 4*		**CLPB**	*ClpB caseinolytic peptidase B homolog (E. coli)*	
**CHORDC1**	*cysteine and histidine-rich domain (CHORD) containing 1*		**NEK9**	*NIMA-related kinase 9*	
**FAM63A**	*family with sequence similarity 63, member A*		**MMAB**	*methylmalonic aciduria (cobalamin deficiency) cblB type*	
**ISM2**	*isthmin 2*		**CYB5R4**	*cytochrome b5 reductase 4*	
**HMBOX1**	*homeobox containing 1*	✓	**DPH5**	*diphthamide biosynthesis 5*	
**TMEM135**	*transmembrane protein 135*	✓	**UBE2J1**	*ubiquitin-conjugating enzyme E2, J1*	
**RNF111**	*ring finger protein 111*		**FMNL3**	*formin-like 3*	
**ZNF423**	*zinc finger protein 423*		**MAP2K6**	*mitogen-activated protein kinase kinase 6*	
**RGP1**	*RGP1 retrograde golgi transport homolog (S. cerevisiae)*		**DENND4A**	*DENN/MADD domain containing 4A*	
**EIF4E**	*eukaryotic translation initiation factor 4E*		**IP6K1**	*inositol hexakisphosphate kinase 1*	
**TAF7L**	*TAF7-like RNA polymerase II, TATA box binding protein (TBP)-associated factor, 50kDa*		**U2SURP**	*U2 snRNP-associated SURP domain containing*	✓

**PIK3R1**	*phosphoinositide-3-kinase, regulatory subunit 1 (alpha)*	✓	**NUDT5**	*nudix (nucleoside diphosphate linked moiety X)-type motif 5*	
**EXTL3**	*exostosin-like glycosyltransferase 3*		**PHKG2**	*phosphorylase kinase, gamma 2 (testis)*	
**HEPHL1**	*hephaestin-like 1*		**DCAF10**	*DDB1 and CUL4 associated factor 10*	
**SLC7A1**	*solute carrier family 7 (cationic amino acid transporter, y+ system), member 1*		**C6orf57**	*chromosome 6 open reading frame 57*	
**LURAP1**	*leucine rich adaptor protein 1*		**NUP37**	*nucleoporin 37kDa*	
**SLC6A4**	*solute carrier family 6 (neurotransmitter transporter), member 4*		**IKBKAP**	*inhibitor of kappa light polypeptide gene enhancer in B-cells, kinase complex-associated protein*	
**PTCH1**	*patched 1*		**TPT1**	*tumor protein, translationally-controlled 1*	
**WWP1**	*WW domain containing E3 ubiquitin protein ligase 1*		**NCKAP1**	*NCK-associated protein 1*	✓
**TLCD2**	*TLC domain containing 2*		**CTNNBL1**	*catenin, beta like 1*	
**SRP72**	*signal recognition particle 72kDa*		**CADM1**	*cell adhesion molecule 1*	
**PSAT1**	*phosphoserine aminotransferase 1*	✓	**GSK3B**	*glycogen synthase kinase 3 beta*	✓
**ABCF1**	*ATP-binding cassette, sub-family F (GCN20), member 1*		**CDK1**	*cyclin-dependent kinase 1*	✓
**ARHGAP5**	*Rho GTPase activating protein 5*		**RPS6KA6**	*ribosomal protein S6 kinase, 90kDa, polypeptide 6*	
**PRPH2**	*peripherin 2 (retinal degeneration, slow)*		**RMND5B**	*required for meiotic nuclear division 5 homolog B (S. cerevisiae)*	
**ZYX**	*zyxin*		**UBE2K**	*ubiquitin-conjugating enzyme E2K*	
**YRDC**	*yrdC N(6)-threonylcarbamoyltransferase domain containing*	✓	**C15orf61**	*chromosome 15 open reading frame 61*	
**SLC25A37**	*solute carrier family 25 (mitochondrial iron transporter), member 37*		**RS1**	*retinoschisin 1*	
**HIPK3**	*homeodomain interacting protein kinase 3*		**VEZT**	*vezatin, adherens junctions transmembrane protein*	
**FBXO33**	*F-box protein 33*		**MSH5**	*mutS homolog 5*	
**BPTF**	*bromodomain PHD finger transcription factor*		**HMGCR**	*3-hydroxy-3-methylglutaryl-CoA reductase*	
**COL4A3BP**	*collagen, type IV, alpha 3 (Goodpasture antigen) binding protein*		**CMC1**	*COX assembly mitochondrial protein 1 homolog (S. cerevisiae)*	
**CLCN3**	*chloride channel, voltage-sensitive 3*		**ZBTB20**	*zinc finger and BTB domain containing 20*	
**EFCAB4A**	*EF-hand calcium binding domain 4A*		**KCNK10**	*potassium channel, subfamily K, member 10*	

**CECR6**	*cat eye syndrome chromosome region, candidate 6*		**DCUN1D5**	*DCN1, defective in cullin neddylation 1, domain containing 5*	
**FBXO17**	*F-box protein 17*		**GPR174**	*G protein-coupled receptor 174*	
**THUMPD1**	*THUMP domain containing 1*		**KCNC2**	*potassium voltage-gated channel, Shaw-related subfamily, member 2*	
**RANBP3**	*RAN binding protein 3*		**TNMD**	*tenomodulin*	
**LRCH3**	*leucine-rich repeats and calponin homology (CH) domain containing 3*		**PKIA**	*protein kinase (cAMP-dependent, catalytic) inhibitor alpha*	
**GCNT4**	*glucosaminyl (N-acetyl) transferase 4, core 2*		**MALSU1**	*mitochondrial assembly of ribosomal large subunit 1*	
**PCDH17**	*protocadherin 17*		**RGPD4**	*RANBP2-like and GRIP domain containing 4*	
**SPOCK3**	*sparc/osteonectin, cwcv and kazal-like domains proteoglycan (testican) 3*		**WISP1**	*WNT1 inducible signaling pathway protein 1*	
**IL10RA**	*interleukin 10 receptor, alpha*		**TIMELESS**	*timeless circadian clock*	
**COPS7B**	*COP9 signalosome subunit 7B*		**RGPD8**	*RANBP2-like and GRIP domain containing 8*	
**CNIH2**	*cornichon family AMPA receptor auxiliary protein 2*		**NFAM1**	*NFAT activating protein with ITAM motif 1*	
**ANKIB1**	*ankyrin repeat and IBR domain containing 1*		**CSMD1**	*CUB and Sushi multiple domains 1*	
**EPHA1**	*EPH receptor A1*		**PDHB**	*pyruvate dehydrogenase (lipoamide) beta*	
**EML6**	*echinoderm microtubule associated protein like 6*		**PRR11**	*proline rich 11*	
**EIF5A**	*eukaryotic translation initiation factor 5A*		**KCNK15**	*potassium channel, subfamily K, member 15*	
**CAMSAP1**	*calmodulin regulated spectrin-associated protein 1*	✓	**HCFC2**	*host cell factor C2*	✓
**LAMC1**	*laminin, gamma 1 (formerly LAMB2)*	✓	**SGIP1**	*SH3-domain GRB2-like (endophilin) interacting protein 1*	
**KATNBL1**	*katanin p80 subunit B-like 1*		**ESRP2**	*epithelial splicing regulatory protein 2*	

Table S4. Combination Indexes (CI) in key experiments. Combinations Indexes (CI) were calculated using Chou and Talalay method. CI indexes were calculated using data presented in Figure 4 and Figure 5 of the main text.

Combined treatment	Combination Index (CI)
R8-PNA-a15b+R8-PNA-a425	0.52
R8-PNA-a15b+R8-PNA-a584	0.73

R8-PNA-a15b+R8-PNA-a425+R8-PNA-a584	0.69
R8-PNA-a425+sulforaphane (SFN)	0.40
R8-PNA-a584+sulforaphane (SFN)	0.44
